# *Cdh5-*mediated *Fpn1* deletion exerts neuroprotective effects during the acute phase and inhibitory effects during the recovery phase of ischemic stroke

**DOI:** 10.1038/s41419-023-05688-1

**Published:** 2023-02-25

**Authors:** Huiwen Zheng, Xin Guo, Shaomeng Kang, Zhongda Li, Tian Tian, Jianhua Li, Fudi Wang, Peng Yu, Shiyang Chang, Yan-zhong Chang

**Affiliations:** 1 Laboratory of Molecular Iron Metabolism, Ministry of Education Key Laboratory of Molecular and Cellular Biology, Hebei Key Laboratory of Animal Physiology, Biochemistry and Molecular Biology, College of Life Sciences, Hebei Normal University, Shijiazhuang, 050024 Hebei Province China; 2 Neuromedical Technology Innovation Center of Hebei Province, Brain Aging and Cognitive Neuroscience Laboratory of Hebei Province, Department of Neurology, The First Hospital of Hebei Medical University, Shijiazhuang, 050000 Hebei Province China; 3 Department of Neurology, Hebei Hospital, Xuanwu Hospital of Capital Medical University, Shijiazhuang, 050000 Hebei Province China; 4 The Second Affiliated Hospital, School of Public Health, State Key Laboratory of Experimental Hematology, Zhejiang University School of Medicine, Hangzhou, 310058 Zhejiang Province China; 5 The First Affiliated Hospital, Basic Medical Sciences, School of Public Health, Hengyang Medical School, University of South China, Hengyang, 421001 Hunan Province China; 6 Department of Histology and Embryology, Hebei Medical University, Shijiazhuang, 050017 Hebei Province China

**Keywords:** Stroke, Stroke

## Abstract

Ischemic stroke is associated with high mortality and morbidity rates worldwide. However, the molecular mechanisms underlying the neuronal damage incurred by stroke victims remain unclear. It has previously been reported that ischemic stroke can induce an increase in the levels of brain iron, which is an important factor of in the associated brain damage. Ferroportin 1 (FPN1), the only known cellular iron export protein, is found in brain microvascular endothelial cells (BMVECs) at the blood-brain barrier, and is considered the gateway for entry of plasma iron into the central nervous system. Despite the connection of brain iron to neuronal damage, the role of BMVECs FPN1 in ischemic stroke remains unexplored. Herein, we conditionally deleted *Fpn1* in mouse endothelial cells (ECs), using VE-cadherin-Cre transgenic mice, and explored the impact on brain iron homeostasis after stroke. Our data demonstrated that *Fpn1* knockout in ECs decreased the brain iron levels in mice, attenuated the oxidative stress and inflammatory responses after stroke, and inhibited both ferroptosis and apoptosis, ultimately alleviating neurological impairment and decreasing cerebral infarct volume during the acute phase of ischemic stroke. By contrast, we found that *Fpn1* knockout in ECs delayed the recovery of neurological function in mice following ischemic stroke. We also found that ECs *Fpn1* knockout decreased the brain iron levels after stroke, exacerbated glial cell proliferation, and inhibited neuronal development, indicating that the diminished brain iron levels hindered the repair of neural injury in mice. In conclusion, our findings reveal a dual consequence of FPN1 deficiency in ECs in the development of ischemic stroke. More specifically, iron deficiency initially exerts a neuroprotective effect during the acute phase of ischemic stroke but inhibits recovery during the later stages. Our findings are important to the development of iron- or FPN1-targeting therapeutics for the treatment of ischemic stroke.

## Introduction

Due to the associated high morbidity, mortality, and disability rates, ischemic stroke is a major health concern worldwide [1, 2]. In the context of the clinic, the treatment measures available for ischemic stroke are limited and primarily comprise intravenous and arterial thrombolysis [3], which tend to have both short therapeutic time windows and many associated complications [4]. Although drug treatments have been found to improve the neurological deficits in animal models of cerebral ischemia, the therapeutics are rarely found safe and effective in the clinic [5, 6]. Thus, there remains a severe lack of an effective treatment for ischemic stroke.

The process of nerve injury following cerebral ischemia involves dysregulated iron metabolism, excitatory neurotoxicity, oxidative stress, the release of inflammatory factors, and cell death [7, 8]. Prior studies have also demonstrated changes in brain morphology, liquefaction and necrosis of nerve cells, and iron deposition in the ischemic area after cerebral ischemia injury [9–12].This increase in iron deposition leads to the aggravation of oxidative stress [13], increases the inflammatory response [14, 15], ultimately causing cell death by apoptosis and ferroptosis [16–18]. Microglia, as central nervous system immune cells, are activated within a few minutes of ischemic injury [19–21], initiating an inflammatory response and secreting IL-6, IL-1, TNF-α, and other proinflammatory factors [22], all of which are neurotoxic. Meanwhile, the hyperactivated microglia, together with astrocytes, promote gliosis and inhibit both neuron regeneration and migration, thereby aggravating the nerve injury [23, 24].

As an essential element within the human body, iron participates in various physiological and biochemical reactions [25]. Iron is the main cofactor in numerous proteases, in addition to being involved in the synthesis of DNA, RNA, and protein [26–28]. In the central nervous system, iron is required for myelination and neurotransmitter synthesis [29]. When brain iron levels are low, neuron development is impaired and demyelination may occur [30]. By contrast, brain iron overload can lead to neurodegenerative diseases [31, 32]. Thus, the precise control of brain iron homeostasis is essential to the maintenance of the normal function of the central nervous system [33–35].

The blood–brain barrier (BBB) comprises the brain capillary walls and glial cells [36–38]. It is composed of tightly connected capillary endothelial cells, pericytes, and astrocytes [39]. Brain microvascular endothelial cells (BMVECs) limit the intercellular and extracellular transport pathways [7, 40]. As the only known iron-exporting protein, ferroportin 1 (FPN1), present in BMVECs, is an important molecule with regard to the entry of iron into the brain from the blood [41]. Previous reports have shown that when FPN1 is absent from small intestinal epithelial cells [42], iron obtained through dietary means cannot enter the circulation and instead accumulates in the enterocytes. In our previous investigations, we found that mice with selective ECs *Fpn1* knockout led to a significant decrease in brain iron levels, with concomitant impairment in memory and cognitive functions, environmental perception, and motor balance [43]. Considering these findings, it becomes important to determine the role of BMVECs FPN1 in regulating brain iron levels following ischemic stroke injury. It is also necessary to identify the mechanisms controlling iron balance in the recovery process following stroke.

The aim of our present study was to further understand the mechanism of cerebral ischemia and reveal novel therapeutic targets in relation to both injury and recovery. To this end, we generated 3-month-old VE-cadherin-Cre (*Cdh5*-Cre) transgenic mice to explore the impact of BMVECs FPN1 in brain iron homeostasis after stroke. We used a right focal cortical infarction model to investigate the acute and recovery stages of cerebral ischemia in the mice. In one set of experiments, we assessed the neurological injury during the acute phase of cerebral ischemia in the mice to examine whether decrease in the brain iron levels associated with FPN1 deficiency decreases the ischemia-associated oxidative stress, inflammatory response, and cell death, thereby alleviating nerve injury. In subsequent experiments, we evaluated the neurological function of the mice following cerebral ischemia during the recovery period to determine whether the decreases in the brain iron levels may aggravate gliosis and inhibit nerve regeneration, resulting in delayed recovery of neurological function. Our study is the first to demonstrate the effects of conditional, endothelial cell knockout of the ferroportin 1 gene in a model of ischemic stroke. We demonstrate that in the acute phase of ischemic stroke, blockage of iron uptake is neuroprotective, whereas the longer-term effects of FPN1 deficiency interfere with stroke recovery. Our data both identify iron uptake into the brain, and FPN1 in particular, as a target for the treatment of ischemic stroke at the acute phase, while demonstrating that long-term limitation of brain iron import will impair stroke recovery.

## Results

### *Fpn1* knockout in ECs decreases cerebral infarct volume and attenuates the neurological function impairments in the acute stage of ischemic stroke

**Fig. 1:**
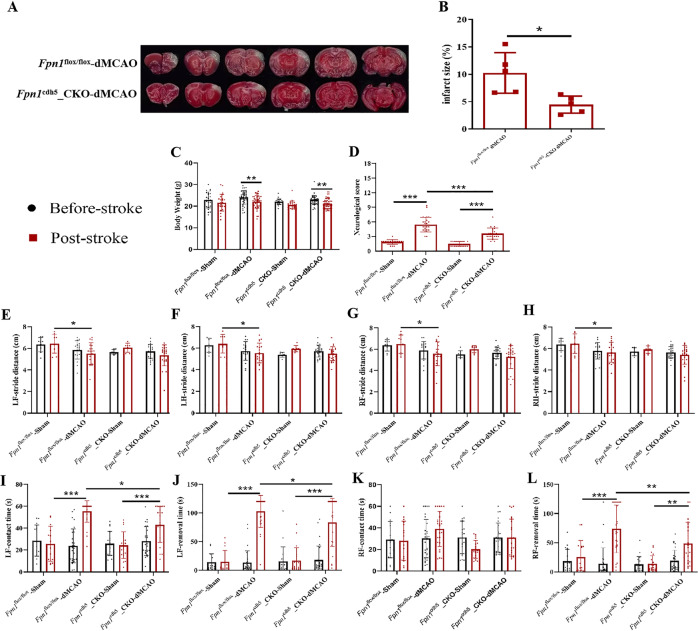
*Fpn1* knockout in ECs decreases cerebral infarct volume and attenuates the neurological function impairments in the acute stage of ischemic stroke. **A** Representative TTC-stained sections on Day 1 after surgery. **B** Quantification of infarction volume ratio (*n* = 5, unpaired *t*-test). The body weight (**C**), mNSS (**D**), gait analysis (**E**–**H**), adhesive removal test (**I**–**L**) were measured in the sham group and dMCAO group at Day 1 after dMCAO (The number of animals used in each experiment, two-way ANOVA, and Tukey’s *post hoc* comparisons are provided in Supplemental Table 2).The data are presented as the mean ± SD. *p < 0.05, **p < 0.01, ***p < 0.001.

To study the effect of *Fpn1* knockout in ECs on ischemic stroke, TTC staining was carried out on Day 1 after surgery. The infarct brain tissues appeared white by TTC staining in mice after surgery, demonstrating successful model construction (Fig. 1A). Compared with the *Fpn1*^flox/flox^-dMCAO group, the cerebral infarct volume decreased in the *Fpn1*^cdh5^-CKO-dMCAO group, and the difference was statistically significant (Fig. 1B, *p* < 0.05). The mNSS, gait analysis, and adhesive removal tests were used to evaluate the neurological function of mice before the operation and on Day 1 after the operation. Compared with the pre-operation data, the body weight of the mice in the *Fpn1*^flox/flox^-dMCAO group and *Fpn1*^cdh5^-CKO-dMCAO group decreased on Day 1 after the operation (Fig. 1C, *p* < 0.01), there was no significant difference between the two groups. When compared with the *Fpn1*^flox/flox^-dMCAO group, the mNSS score of the *Fpn1*^cdh5^-CKO-dMCAO group was significantly lower (Fig. 1D, *p* < 0.001). The gait analysis revealed that, when compared with the *Fpn1*^flox/flox^-sham group, the step amplitude of the left forelimbs (Fig. 1E, *p* < 0.05), left hindlimbs (Fig. 1F, *p* < 0.05), right forelimbs (Fig. 1G, *p* < 0.05) and right hindlimbs (Fig. 1H, *p* < 0.05) decreased significantly in the *Fpn1*^flox/flox^-dMCAO group, while there was no significant change observed in the *Fpn1*^cdh5^-CKO-dMCAO group. The adhesive removal test revealed that, when compared with the *Fpn1*^flox/flox^-dMCAO group, the left limb contact timer (Fig. 1I, *p* < 0.05), left limb removal time (Fig. 1J, *p* < 0.05), and right limb removal time (Fig. 1L, *p* < 0.01) of the *Fpn1*^cdh5^-CKO-dMCAO group were significantly shorter. Taken together the data indicate that ECs *Fpn1* deficiency significantly decreases cerebral infarct volume and attenuates the neurological impairments seen in the mice in the acute stage of ischemic stroke.

### *Fpn1* knockout in ECs decreases brain iron deposition during the acute stage of ischemic stroke

**Fig. 2:**
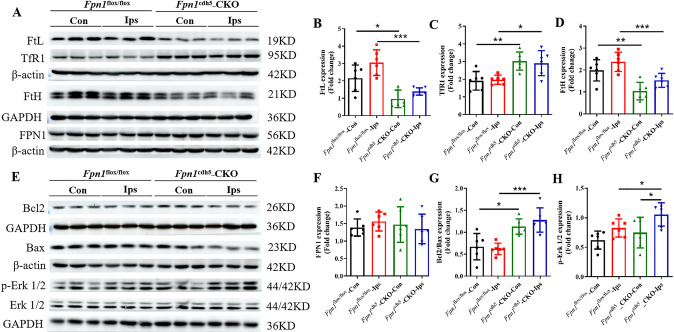
*Fpn1* knockout in ECs decreases brain iron accumulation and apoptosis associated with the acute stage of ischemic stroke. Representative western blot images of the levels of FtL, TfR1, FtH and FPN1 (**A**) in the contralateral (Con) and ipsilateral (Ips) cerebral cortex on Day 1 after dMCAO. Quantification of the western blot signals of FtL (**B**), TfR1 (**C**), FtH (**D**), and FPN1 (**F**). **E** Representative western blot images of the levels of Bcl2, Bax, and p-Erk1/2 in the contralateral (Con) and ipsilateral (Ips) cerebral cortex at Day 1 after dMCAO. Quantification of the western blot signals of Bcl2/Bax ratio (**G**), and p-Erk1/2 (**H**). β-actin or GAPDH was used as an internal control. The data are presented as the mean ± SD. *n* = 6, two-way ANOVA, Tukey’s *post hoc* comparisons, *p < 0.05, **p < 0.01, ***p < 0.001.

Ischemic stroke alters iron metabolism in the brain, and increased iron level is associated with an early worsening of neurological function. To explore how *Fpn1* knockout in ECs affects neuronal damage, we initially examined the expression of iron metabolism-related proteins by WB analysis on Day 1 after the dMCAO operation. When compared with the *Fpn1*^flox/flox^-dMCAO group, the expression of iron storage protein ferritins FtL and FtH on the lesion side in the *Fpn1*^cdh5^-CKO-dMCAO group decreased significantly one day after the operation (Fig. 2A, B, D, *p* < 0.001), while the expression of the iron uptake protein, TfR1, increased significantly (Fig. 2A, C, *p* < 0.05), and the expression of the only cellular iron exporter, FPN1, did not change significantly (Fig. 2A, F). These results suggest that *Fpn1* knockout in ECs decreases the brain iron levels in the mice in the acute stage of the stroke model.

### *Fpn1* knockout in ECs attenuates both apoptosis and ferroptosis during the acute phase of ischemic stroke

**Fig. 3:**
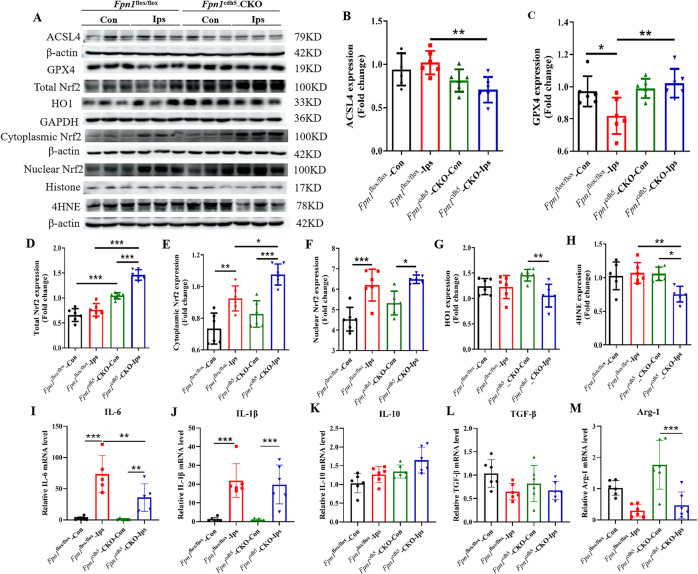
*Fpn1* knockout in ECs attenuates ferroptosis, and alleviates the oxidative stress and inflammatory response that occur in the acute phase of ischemic stroke. **A**. Representative western blot images of the levels of ACSL4 and GPX4, total Nrf2, cytoplasmic Nrf2, nuclear Nrf2, HO1 and 4HNE in the contralateral (Con) and ipsilateral (Ips) cerebral cortex at Day 1 after dMCAO. Quantification of the western blot signals of ACSL4 (**B**), GPX4(**C**), total Nrf2 (**D**), Cytoplasmic Nrf2 (**E**), Nuclear Nrf2 (**F**), HO1 (**G**), and 4HNE (**H**). β-actin or GAPDH or histone was used as an internal control. Quantification by qRT-PCR of IL-6 (**I**), IL-1β (**J**), IL-10 (**K**) TGF-β (**L**), and Arg-1 (**M**) mRNA levels. β-actin was used as an internal control. The data are presented as the mean ± SD. *n* = 6, two-way ANOVA, Tukey’s *post hoc* comparisons. *p < 0.05, **p < 0.01, ***p < 0.001.

To further clarify the mechanisms that affect brain iron homeostasis in ECs following conditional *Fpn1* knockout after ischemic stroke, We assessed the expression of apoptosis-related proteins by WB analysis. When compared with the *Fpn1*^flox/flox^-dMCAO group, both the ratio of Bcl-2/Bax (Fig. 2E, G, *p* < 0.001) and the expression of p-ERK1/2 (Fig. 2E, H, *p* < 0.05) on the lesion side was increased significantly in the *Fpn1*^cdh5^-CKO-dMCAO group. Ferroptosis, a form of cell death that entails iron-catalyzed lipid peroxidation, is closely linked to the occurrence and development of stroke, and a common pathway of cell death in ischemic stroke. we also examined the expression of the marker molecules of ferroptosis, ACSL4 and GPX4, by WB analysis. When compared with the *Fpn1*^flox/flox^-dMCAO group, the expression of ACSL4 on the lesion side in the *Fpn1*^cdh5^-CKO-dMCAO group was significantly decreased (Fig. 3A, B, *p* < 0.01), while the expression of GPX4 was significantly increased (Fig. 3A, C, *p* < 0.01). These results suggest that *Fpn1* knockout in ECs attenuates apoptosis and ferroptosis, and activates the ERK1/2 pathway in the acute phase of ischemic stroke.

### *Fpn1* knockout in ECs alleviates the oxidative stress and inflammation response during the acute stage of ischemic stroke

Iron metabolism is closely related to both oxidative stress and inflammation. We hypothesized that *Fpn1* knockout in ECs may alter cerebral ischemia-induced oxidative stress and inflammation through affecting free iron accumulation. We performed WB analysis to assess oxidative stress-related proteins on Day 1 after the operation. As shown in Fig. 3, when compared with the *Fpn1*^flox/flox^-dMCAO group, the expression of total Nrf2 (Fig. 3A, D, *p* < 0.001) and cytoplasmic Nrf2 (Fig. 3A, E, *p* < 0.05) on the lesion side in the *Fpn1*^cdh5^-CKO-dMCAO group was significantly greater, while the expression of 4HNE decreased significantly (Fig. 3A, H, *p* < 0.01) and the expression of both nuclear Nrf2 (Fig. 3A, F) and total HO-1 (Fig. 3A, G) increased, albeit not statistically significantly. The levels of inflammatory factors, as detected by qRT-PCR analysis, revealed that, when compared with the contralateral side, the mRNA expression of the pro-inflammatory factors, IL-6 (Fig. 3I, *p* < 0.01) and IL-1β (Fig. 3J, *p* < 0.001) on the lesion side in the *Fpn1*^flox/flox^-dMCAO and *Fpn1*^cdh5^-CKO-dMCAO groups was significantly increased. Moreover, when compared with the *Fpn1*^flox/flox^-dMCAO group, the mRNA expression of IL-6 on the lesion side in the *Fpn1*^cdh5^-CKO-dMCAO group was significantly lower (Fig. 3I, *p* < 0.01), and there was no significant change in the mRNA expression of IL-1β, IL-10, TGF-β, or Arg-1. These results demonstrate that *Fpn1* knockout in ECs alleviates both the oxidative stress and the inflammatory response in the mice on Day 1 following ischemic stroke.

### *Fpn1* knockout in ECs delays the early recovery of neurological function in mice with ischemic stroke

**Fig. 4:**
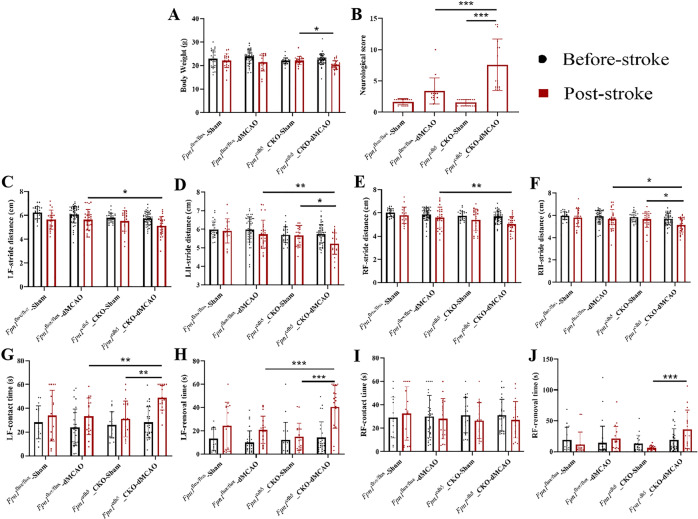
*Fpn1* knockout in ECs delays the early recovery of neurological function in mice with ischemic stroke. Quantification of body weight (**A**), mNSS (**B**), gait analysis (**C**–**F**), and adhesive removal test (**G**–**J**) in the sham and dMCAO groups at Day 7 after dMCAO. The data are presented as the mean ± SD. The number of animals used in each experiment, two-way ANOVA, and Tukey’s *post hoc* comparisons are provided in Supplemental Table 2. *p < 0.05, **p < 0.01, ***p < 0.001.

To explore the effects of a decrease in brain iron on the recovery of neurological function in mice during the later stages of stroke, we conducted mNSS, gait analysis, and adhesive removal tests on Day 7 after the operation. Compared with the *Fpn1*^flox/flox^-dMCAO group, the body weight in the *Fpn1*^cdh5^-CKO-dMCAO group was no significantly reduced (Fig. 4A), the mNSS score was significantly higher (Fig. 4B, *p* < 0.001), the step amplitudes of the left and right forelimbs and hindlimbs were significantly decreased (Fig. 4C–F, *p* < 0.05), the left limb contact time (Fig. 4G, *p* < 0.01) and removal time (Fig. 4H, *p* < 0.001) were significantly increased, and right limb contact time and removal time were no significantly increased (Fig. 4I, J). The neuroethological data indicate that EC *Fpn1* deficiency delays the rescue of neurological function in the early recovery stage after ischemic stroke.

### *Fpn1* knockout in ECs decreases cerebral iron levels during the early recovery stage of ischemic stroke

**Fig. 5:**
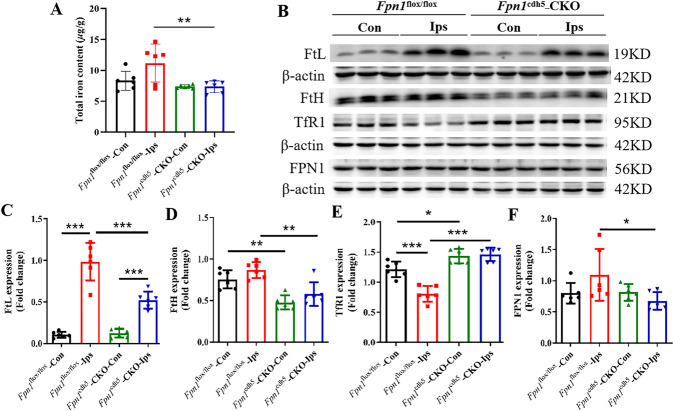
*Fpn1* knockout in ECs decreases cerebral iron levels during the early recovery stage of ischemic stroke. **A** The total iron content in the cortex detected by ICP-MS at Day 7 after dMCAO. **B** Representative western blot images of the levels of FtL, FtH, TfR1, and FPN1 in the contralateral (Con) and ipsilateral (Ips) cerebral cortex at Day 7 after dMCAO. Quantification of the western blot signals of FtL (**C**), FtH (**D**), TfR1 (**E**), and FPN1 (**F**). β-actin or GAPDH was used as an internal control. The data are presented as the mean ± SD. *n* = 6, two-way ANOVA, Tukey’s *post hoc* comparisons. *p < 0.05, **p < 0.01, ***p < 0.001.

**Fig. 6:**
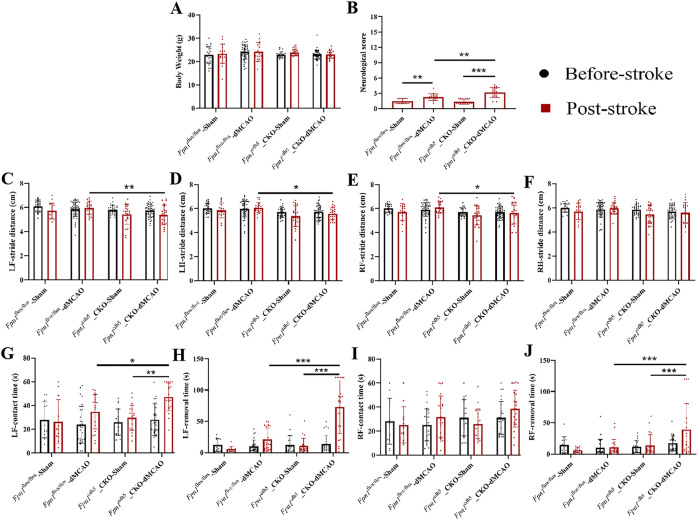
*Fpn1* knockout in ECs does not completely restore the neurological status of mice following ischemic stroke. Quantification of body weight (**A**), mNSS (**B**), gait analysis (**C**–**F**), and adhesive removal test (**G**–**J**) in the sham and dMCAO groups at Day 28 after dMCAO. The data are presented as the mean ± SD. The number of animals used in each experiment, two-way ANOVA, and Tukey’s *post hoc* comparisons are presented in Supplemental Table 2. *p < 0.05, **p < 0.01, ***p < 0.001.

Iron is essential for cell growth and differentiation, and is also critical to cell survival and maturation. We expected that iron would be unable to efficiently enter the brain through the BBB after *Fpn1* knockout in ECs. Thus, we examined the iron content in the cortex by ICP-MS on Day 7 after ischemic stroke. As shown in Fig. 6A, when compared with the *Fpn1*^flox/flox^-dMCAO group, *Fpn1* knockout in ECs effectively decreased the brain iron content on the lesion side in the *Fpn1*^cdh5^-CKO-dMCAO groupn (Fig. 5A, *p* < 0.01). In addition, we evaluated the expression of iron metabolism-related proteins by WB analysis. Compared with the *Fpn1*^flox/flox^-dMCAO group, the expression of FtL (Fig. 5B, C, *p* < 0.001), FtH (Fig. 5B, D, *p* < 0.01) and FPN1 (Fig. 5B, F, *p* < 0.05) on the lesion side in the *Fpn1*^cdh5^-CKO-dMCAO group was significantly decreased, while the TfR1 expression showed a significant increase (Fig. 5B, E, *p* < 0.001). Our data confirm that ECs *Fpn1* deficiency leads to diminished cerebral iron levels in the early recovery stage after ischemic stroke.

### *Fpn1* knockout in ECs is insufficient to restore the neurological status of mice following ischemic stroke

**Fig. 7:**
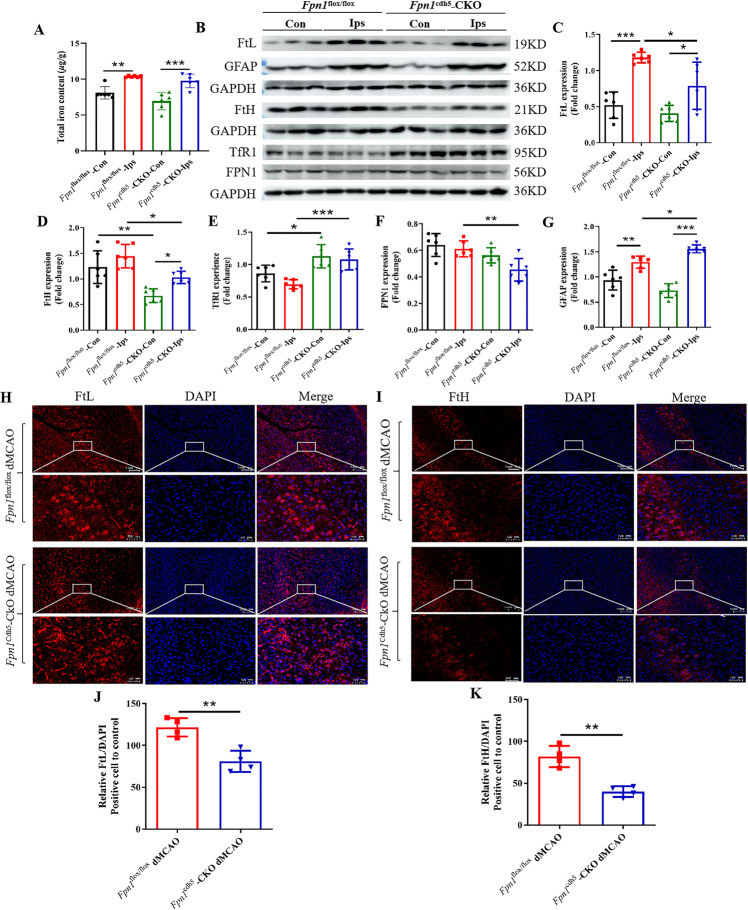
*Fpn1* knockout in ECs diminishes brain iron accumulation during the long-term recovery period following ischemic stroke. **A** The levels of total iron content in the cortex detected by ICP-MS at Day 28 after dMCAO (*n* = 6, two-way ANOVA, Tukey’s *post hoc* comparisons). **B** Representative western blot images of the levels of FtL, FtH, TfR1, FPN1 and GFAP in the contralateral (Con) and ipsilateral (Ips) cerebral cortex at Day 28 after dMCAO. Quantification of the western blot signals of FtL (**C**), FtH (**D**), TfR1 (**E**), FPN1 (**F**) and GFAP (**G**) (*n* = 6, two-way ANOVA, Tukey’s *post hoc* comparisons). GAPDH was used as an internal control. The distribution of FtL (**H**) and FtH (**I**) in the cortex detected by immunofluorescence staining (scale bar = 50 μm). Quantification of immunofluorescence staining of FtL (**J**) and FtH (**K**) (*n* = 4, unpaired *t*-test). The data are presented as the mean ± SD. *p < 0.05, **p < 0.01, ***p < 0.001.

The above-mentioned experiments demonstrate that *Fpn1* knockout in ECs decreases the cerebral iron levels and delays the recovery of neurological function in the mice on Day 7 after ischemic stroke. However, the consequences to neuron regeneration remains to be elucidated. Therefore, we continued to observe the neurological function recovery on Day 28 after the operation. As shown in Fig. 7, when compared with the *Fpn1*^flox/flox^-dMCAO group, while there was no significant difference in body weight of the *Fpn1*^cdh5^-CKO-dMCAO group (Fig. 6A), the mNSS score in the *Fpn1*^cdh5^-CKO-dMCAO group was still significantly increased (Fig. 6B, *p* < 0.01). Moreover, the gait analysis showed that, compared with the *Fpn1*^flox/flox^-dMCAO group, the step amplitudes of the left forelimbs (Fig. 6C, *p* < 0.01) and left hindlimbs (Fig. 6D, *p* < 0.05), and the right forelimbs (Fig. 6E, *p* < 0.05) were significantly reduced, while the step amplitudes of the right hindlimbs were not significantly changed in the *Fpn1*^cdh5^-CKO-dMCAO group (Fig. 6F). The adhesive removal test indicated that, compared with the *Fpn1*^flox/flox^-dMCAO group, the contact time of the left limb (Fig. 6G, *p* < 0.05) and the removal times of the left limb (Fig. 6H, *p* < 0.001) and right limb (Fig. 6J, *p* < 0.001) were significantly prolonged in the *Fpn1*^cdh5^-CKO-dMCAO group. Collectively, these results suggest that *Fpn1* knockout in ECs is not conducive to the long-term recovery of neurological function in mice after a neuronal regeneration cycle following ischemic stroke.

### *Fpn1* knockout in ECs leads to decreased brain iron levels during the long-term recovery period following ischemic stroke

To further explore the alterations in cerebral iron levels after *Fpn1* knockout in ECs during the long-term recovery stage of stroke, we measured both the total iron content and the expression of iron metabolism-related proteins on Day 28 after stroke by ICP-MS, WB analysis, and immunofluorescence staining. The ICP-MS analysis indicated that the iron content was increased in the damaged areas of brain (Fig. 7A, *p* < 0.01), but lower in the *Fpn1*^cdh5^-CKO-dMCAO group than in the *Fpn1*^flox/flox^-dMCAO group, although there was no significant difference between the two groups (Fig. 7A). In addition, in line with the decrease in the brain iron content, a notable decrease in FtL (Fig. 7B, C, *p* < 0.05) and FtH (Fig. 7B, D, *p* < 0.05) expression was also observed in peri-infarct of the *Fpn1*^cdh5^-CKO-dMCAO group. Compared with the *Fpn1*^flox/flox^-dMCAO group, the expression of FPN1 (Fig. 7B, F, *p* < 0.01) on the lesion side in the *Fpn1*^cdh5^-CKO-dMCAO group decreased significantly, while the expression of TfR1 increased significantly (Fig. 7B, E, *p* < 0.001). In addition, we also examined the distribution of FtL and FtH by immunofluorescence staining. Similar to the reduction in brain iron content, a notable decrease of FtL and FtH plaques was observed in the *Fpn1*^cdh5^-CKO-dMCAO group as well (Fig. 7H–K, *p* < 0.01). These results are congruent with a decrease in brain iron levels in *Fpn1*-deficient ECs in the stroke model mice on Day 28 after ischemic stroke.

### *Fpn1* knockout in ECs exacerbates astrocyte hyperplasia and inhibits neuronal development during the long-term recovery period following ischemic stroke

**Fig. 8:**
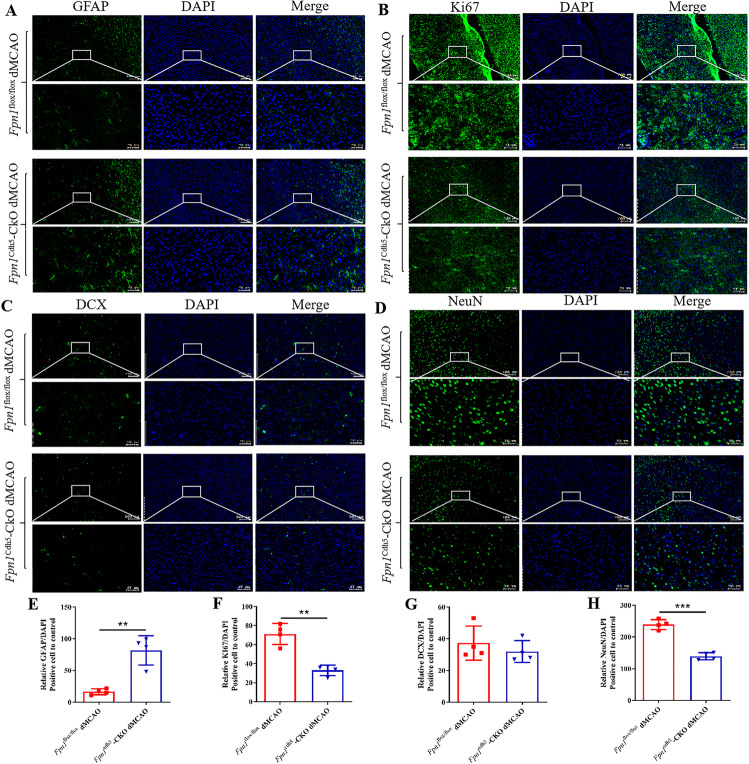
*Fpn1* knockout in ECs exacerbates glial hyperplasia and inhibits neuronal development during the long-term recovery period following ischemic stroke. The distribution of GFAP (**A**), Ki67(**B**), DCX (**C**), and NeuN (**D**) in the cortex detected by immunofluorescence staining (scale bar = 50 μm). Quantification of immunofluorescence staining of GFAP (**E**), Ki67(**F**), DCX (**G**), and NeuN (**H**) (*n* = 4, unpaired *t*-test). The data are presented as the mean ± SD. *p < 0.05, **p < 0.01, ***p < 0.001.

As mentioned above, *Fpn1* knockout in ECs served to diminish the brain iron levels in the mice. The effects and mechanisms of iron levels on the recovery period of stroke are poorly understood. Prior studies have found that endogenous repair mechanisms, such as gliosis and neuronal development, have perceptible effect on the recovery of nerve function after stroke. Thus, we observed the effects of *Fpn1* knockout in ECs on astrocytes and neurons during the long-term recovery period following ischemic stroke. First, we examined the expression of the astrocyte activation marker, GFAP, on Day 28 after ischemic stroke. The WB results showed that, compared with the *Fpn1*^flox/flox^-dMCAO group, GFAP expression significantly increased on the lesion side in the *Fpn1*^cdh5^-CKO-dMCAO group (Fig. 7B, G, *p* < 0.05). Similar results were observed by immunohistochemical staining (Fig. 8A, E, *p* < 0.01). Stroke can induce subventricular zone cell proliferation. More specifically, the migration of immature and proliferating neurons from the subventricular zone to the tissues surrounding the infarct and then the differentiation of these cells into neurons was involved in the recovery process following ischemic stroke. Ki67, DCX, and NeuN are markers of the proliferation, differentiation, and maturation at different stages of neuronal development, respectively. Therefore, we measured the distribution of these markers by immunofluorescence staining. We found that, compared with the *Fpn1*^flox/flox^-dMCAO group, the Ki67- (Fig. 8B, F, *p* < 0.01) and NeuN-positive cells (Fig. 8D, H, *p* < 0.001) decreased significantly, while the expression of DCX-positive cells did not change significantly (Fig. 8C, G). Taken together, these results suggest that *Fpn1* knockout in ECs exacerbates astrocyte hyperplasia and inhibits neuronal proliferation and development during the long-term recovery period after ischemic stroke.

## Discussion

Ischemic stroke represents a major health concern worldwide. Importantly, due to shifting living and dietary habits, the average age of occurrence of stroke has gradually become younger [44, 45]. In terms of the treatment of stroke, intravenous thrombolysis is the most commonly used method, although the effective time window is limited [3]. In recent years, intravascular therapy has attracted the attention of researchers and clinicians, although this strategy is also known to cause secondary injury (i.e., reperfusion injury) to at least a certain extent [4].

Ischemic stroke can cause serious neurological damage, mainly through oxidative stress [46], inflammation [23], and excitatory neurotoxicity [19, 24]. While iron is essential to neurological function and CNS maintenance, the metal can be viewed as a double-edged sword, as iron overload can also lead to neurodegenerative disease, as observed in the elderly, whereas iron deficiency can lead to neurodevelopmental deficiencies in infants [47–49]. Thus, brain iron homeostasis is vital to human health. Previous studies have shown that, following ischemic stroke, the brain iron content increases significantly [50] to directly stimulate oxidative stress, inflammation, and neurotoxicity. Low brain iron content affects myelination, neurotransmission, and cell division [34]. However, there still remain significant gaps in our understanding of the molecules and mechanisms governing brain iron homeostasis. We hypothesized that *Fpn1* significantly affects brain iron levels *via* ECs in ischemic stroke injury and recovery.

In the present study, we established a dMCAO model in 3-month-old *Cdh5-Cre* transgenic mice to generate brain tissue infarction and the consequent reduced blood flow, which is commonly used in animal models of cerebral ischemia. To investigate the function of FPN1 in ECs on neurological injury in mice with acute cerebral ischemia, we first examined the cerebral infarct volume and evaluated the effects of neurological injury, in terms of body weight, mNSS, adhesive removal, and gait analysise, in the cerebral ischemia mice with or without conditional disruption of *Fpn1*. One day after the operation, i.e., the acute stage of ischemic stroke, *Fpn1* knockout in ECs resulted in reduced cerebral infarct volume, decreased mNSS, shortened contact and removal times of adhesion, and lessened neurological function impairments. Our team has been demonstrated that specific *Fpn1* knockout in ECs will lead to a decrease in brain iron levels. To explore whether there are alterations in iron metabolism after cerebral ischemia, we examined the expression of iron metabolism-related proteins that are regulated by iron. As expected, *Fpn1* knockout in ECs decreased brain iron accumulation mice during the acute stage of ischemic stroke.

Uncommitted iron can catalyze the production of toxic hydroxyl radicals from superoxide and hydrogen peroxide through Fenton chemistry, resulting in cell death via apoptosis and/or ferroptosis. We found that *Fpn1* knockout in ECs decreased both apoptosis and ferroptosis, activating the ERK1/2 pathway, on Day 1 after ischemic stroke. It has previously been reported that, in a rat model of stroke, the activation of the ERK1/2 pathway can effectively protect against nerve injury caused by cerebral ischemia [51]. This is consistent with our finding that *Fpn1* knockout in ECs attenuates the impairments in neurological function during the acute stage of ischemic stroke, likely as a result of decreased catalytic metal in the brain.

Oxidative stress and inflammation can lead to cell death [52–54], we hypothesized that *Fpn1* knockout in ECs may protect against cell death by alleviate cerebral ischemia-induced oxidative stress and inflammation. We found that, on the ischemic side of the brain, disruption of *Fpn1* significantly decreased the expression of 4HNE (marker of oxidative stress), while antioxidant-related protein expression increased, suggesting that the limitation of iron transport via ECs FPN1 can mitigate the deleterious effects of free iron during the acute phase of ischemia. These findings were strengthened by the results of our qRT-PCR experiments, in which the mRNA expression of IL-6 (pro-inflammatory) on the lesion side was significantly lower, while the mRNA expression of IL-10 (anti-inflammatory) was significantly higher, in the mice deficient in ECs FPN1.

While free iron can lead to the production of highly toxic radical species, the metal is essential for cell growth, differentiation, survival, and function. Since, iron cannot enter the brain through the BBB after *Fpn1* knockout in ECs, we further explored if the limitation of brain iron under these circumstances affected the recovery of neurological function in mice following ischemia stroke. We found that the advantage by FPN1 deficiency on Day 1 was reversed in the mice on Day 7 after the operation; the mNSS, adhesion removal, and gait tests revealed delayed neurological function recovery on Day 7 after ischemic stroke. We confirmed by ICP-MS that the iron content in the cerebral cortex of the ischemic mice was significantly lower when *Fpn1* was disrupted in ECs. In addition, levels of the iron storage proteins, FtL and FtH, exhibited a decreasing trend after ECs *Fpn1* knockout, which was congruent with the ICP-MS data, since the ferritin proteins are regulated by cellular iron content. Collectively, these results suggest that knockout of *Fpn1* in ECs delays the recovery of neurological function recovery on Day 7 after ischemic stroke by limiting cerebral iron availability.

Since we observed a delayed recovery of neurological function on Day 7 after stroke when cerebral iron levels were compromised by the disruption of ECs *Fpn1*, we further investigated the consequences of ECs FPN1 deficiency after a neuron regeneration cycle. On Day 28 after the operation, there was an increase in mNSS, prolonged contact and removal times of adhesion, and decreased step amplitudes of the left forelimbs and hindlimbs in the *Fpn1*^cdh5^-CKO-dMCAO mice, indicating that the recovery of neurological function remained inhibited in mice lacking ECs FPN1. At the same time, we found that the expression of iron metabolism-related proteins in the *Fpn1*^cdh5^-CKO-dMCAO group was basically consistent with the results recorded on Day 7 after the operation, demonstrating that ECs *Fpn1* knockout continued to result in diminished brain iron during the long-term recovery period after ischemic stroke.

Previous studies have reported that endogenous repair mechanisms, such as gliosis and nerve regeneration, play important roles in the recovery of nerve function after stroke. Our WB results suggested an increase in glial cells on the lesion side of the ECs FPN1-deficient mice in the long-term recovery phase after stroke. We also found that neural stem cell migration was blocked in this phase in the mice lacking ECs FPN1. Taken together, these results suggest that *Fpn1* knockout in ECs exacerbated glial hyperplasia and inhibited nerve regeneration. We previously demonstrated that treatment with the iron chelator, deferoxamine, decreases brain iron levels with concomitant decreases in the expression of synapse-related proteins, myelin-related proteins, and the proliferation and differentiation of neural stem cells, which ultimately delayed the recovery of neural function [5]. Those findings are consistent with the results of the present study, which together identify the essential role of brain iron in the recovery of cerebral function in mice following ischemia.

To summarize, we found that the specific knockout of *Fpn1* in ECs produced different effects during the acute and recovery phases of ischemic stroke. More specifically, during the acute stage of ischemic stroke, *Fpn1* knockout in ECs led to decreasing the brain iron accumulation, alleviating oxidative stress and the inflammatory response, thereby resulting in a decrease in both ferroptosis and apoptosis, which decreased cerebral infarct volume and attenuated the impairments in neurological function. Conversely, *Fpn1* knockout in ECs delayed the recovery of neurological function in mice following ischemic stroke by diminishing the brain iron accumulation, promoting glial hyperplasia, and inhibiting neural stem cell migration and differentiation, markedly delaying the recovery of neurological function after ischemic stroke. Iron limitation exerts a neuroprotective effect during the acute phase of ischemic stroke and an inhibitory effect during the recovery phase. Thus, future therapeutic strategies for ischemic stroke should examine sequestering free iron in the acute stage of stroke, while ensuring that brain iron is replete in the later stages of stroke recovery.

## Materials and methods

### Animals

In the present study, 3-month-old male *Cdh5*-Cre transgenic mice were selected as experimental animals. We generated *Fpn1*^cdh5^ CKO mice by mating *Cdh5*-Cre recombinase mice on a C57BL/6 background with *Fpn1*^flox/flox^ mice on a 129/SvEvTac background (a gift from Prof. Fudi Wang, Zhejiang University). Standard rodent dry feed was provided, while sterile water was freely available. The mice were kept at 21 ± 1 °C and a relative humidity of 60% ± 5%, and a 12 h light and 12 h dark cycle was maintained. All the experiments in the study were approved by the Animal Care and Use Committee of Hebei Normal University (Shijiazhuang, China). The animal groups comprised *Fpn1*^flox/flox^-Sham mice, *Fpn1*^flox/flox^-dMCAO mice, *Fpn1*^Cdh5^-CKO-Sham mice, and *Fpn1*^cdh5^-CKO-dMCAO mice.

### Mouse model of distal middle cerebral artery occlusion (dMCAO)

The mouse model of dMCAO used in this study was prepared by electrocoagulation [55]. The operating steps for the sham groups were generally the same as those for the dMCAO groups, with the right common carotid artery being isolated without ligation before the skull was exposed and drilled, while the skin layer of the middle cerebral artery was not cauterized. The specific steps are provided below.

The mice were weighed after fasting 12 h and then anesthetized via intraperitoneal injection. After anesthetization, the mice were fixed in a prone position. A midline incision of <1 cm was made at the neck. The subcutaneous tissue was passively separated and then the right common carotid artery (CCA) was carefully dissected, exposed, and permanently ligated. Next, the skin incision was sutured. Subsequently, the mice were fixed in a left lateral recumbent position and then treated with a conventional skin preparation and disinfection at the top of the skull. An incision of around 1 cm was made between the lateral canthus of the right eye and the external auditory canal in order to expose the temporal muscle. At this point, the right cortical branches of the middle cerebral artery were accessible. A small hole with a diameter of around 2 mm was then drilled to additionally expose the right brain. An electrocoagulator was used to cauterize the cortical branches of the middle cerebral artery, with care being taken to avoid damaging the surrounding brain tissues to the greatest extent possible. The skin was sutured after the operation. The mice were kept at a constant temperature of around 37 °C in a humidity-controlled environment until they awoke and regained consciousness.

The mice in the different groups were then graded and euthanized, and materials were collected at the corresponding time points according to the subsequent experimental requirements.

### Neurological function score

The modified neurological severity score (mNSS) was used to evaluate the ability of the mice in sensation, movement, balance, reflex, and other activities [56]. The total score ranged from 0 to 18. The higher the score, the more serious the neurological deficit.

### Adhesion removal test

The adhesion removal test was used to evaluate the sensory motor ability of the mice [57]. Prior to the experiment, the mice were placed in a transparent box for 60 s. During the experiment, the two forelimbs of the mice were pasted to two stickers of the same size (0.3 × 0.4 cm) but different colors. The mice were then placed in a transparent box and observed for 120 s, with the contact time and removal time for the left and right forelimb stickers being recorded. Before modeling, the mice were trained three times a day for 5 days, and any unqualified mice were eliminated. The experiment was performed three times, and the average value was taken as the final result.

### Gait experiment

A gait experiment was conducted to evaluate the motor coordination of the mice [58]. The forelimbs of the mice were coated with red ink, while the hind limbs were coated with black ink. The mice were then placed on white paper (7 × 50 cm) and induced to move forward. The distances between the paw prints of the left and right forelimbs and hind limbs (as indicated by the marks left on the paper) were measured in three consecutive attempts, and the average value was taken as the final value.

### 2,3,5-triphenyltetrazolium chloride (TTC) staining

One day after surgery, the cerebral infarct volume of mice was measured by TTC staining as described previously [5]. After successful anesthesia, the brains were then rapidly extracted. Each brain was sliced into seven coronal brain sections (1 mm). Following that, the sections were placed in 2% TTC (#1.08380, Sigma-Aldrich, USA) for 10 min at 37 °C. After that, they were fixed with 4% paraformaldehyde for 24 h. The slices were photographed by the ultra-depth-of-field microscope and measured by the Image-Pro Plus 5.1 software.

### Western blot (WB) analysis

Protein was extracted from tissues from the mice using RIPA lysis buffer (50.0 mM Tris-HCl pH 7.4, 150.0 mM NaCl, 1% NP40, 0.1% SDS) (68298, Roche Applied Science, Mannheim, Germany) at 15 times the volume of the sample weight (mg). The proteins were resolved by sodium dodecyl sulfate polyacrylamide gel electrophoresis (SDS-PAGE; 10% acrylamide) and then transferred to a nitrocellulose membrane, which was treated at room temperature for 1.5 h with 5% nonfat milk. The membranes were incubated with different primary antibodies: rabbit anti-mouse L-ferritin (FtL) (1:5,000; ab109373, Abcam, USA), rabbit anti-mouse H-ferritin (FtH) (1:10,000; ab183781, Abcam, USA), rabbit anti-mouse FPN1 (1:5,000; MTP11-S, ADI, USA), mouse anti-mouse TfR1 (1:3,000; 13–6800, Invitrogen, USA), rabbit anti-mouse Nrf2 (1:5,000; 16396-1-AP, Proteintech, USA), rabbit anti-mouse HO-1 (1:5,000; 10701-1-AP, Proteintech, China), rabbit anti-4-HNE (1:5,000; HNE11-S, ADI, USA), rabbit anti-mouse GPX4 (1:5,000; ab125066, Abcam, USA), rabbit anti-mouse ACSL4 (1:5,000; ab155282, Abcam, USA), rabbit anti-mouse Bcl-2 (1:5,000; GTX100064, GeneTex, USA), rabbit anti-mouse Bax (1:5,000; #2772, Cell Signaling Technology, USA), rabbit anti-mouse Phospho-Erk and Erk (1:5,000; #4370 and #4695, Cell Signaling Technology, USA); mouse anti-mouse GFAP (1:10,000; MAB360, Sigma-Aldrich, USA), mouse anti-mouse β-actin (1:10,000; CW0096, Cwbio Biotechnology limited company, China) and mouse anti-mouse GAPDH (1:10000; 60004-1-lg, Proteintech, USA) at 4 °C overnight. The next day, the membranes were washed with TBST and then incubated with the corresponding secondary antibody (anti-rabbit secondary antibody- (1:10,000; SA00001-2, Proteintech, China), or anti-mouse secondary antibody-conjugated horse radish peroxide (1:10,000; SA00001-1, Proteintech, China) at room temperature for 1.5 h). The membranes were again washed with TBST and then incubated with luminescence reagents (ECL Western Blot Kit) before the signal was detected with a chemiluminescence instrument (Bio-Rad ChemiDoc Touch). For cases where multiple antibody assays were performed on the same membrane, the exposed membrane was immersed in an appropriate volume of primary and secondary antibody eluent (Sevenbio, beijing, China) and eluted at room temperature for 15 min, then washed with TBST and sealed at room temperature for 1.5 h in 5 % nonfat milk. The membrane was re-incubated with another primary antibody and the next round of western blot experiment was performed. Finally, the relative band intensities of the proteins were quantified and presented as the ratio of the density each protein band to that of β-actin or GAPDH.

### Immunofluorescence staining

The brain tissues of the mice were fixed with 4% paraformaldehyde for 24 h and cryoprotected in 30% sucrose. Coronal sections were cut to a 15-µm thickness using a freezing microtome and stored in a refrigerator. The slices were restored to room temperature for 15 min before use and then washed three times with phosphate-buffered saline (PBS).

High-temperature antigen retrieval was performed in 0.01 M sodium citrate solution (pH: 6.0) for antigen repair, boiled at a high temperature for 10 min, and then cooled naturally to room temperature. Next, the sections were washed three times with PBS. Incubation with 0.3% Triton X-100 was performed for 15 min at room temperature, followed by incubation with diluted goat serum at 37 °C for 50 min. The primary antibodies: anti-L-ferritin antibody (1:600; ab109373, Abcam, USA), anti-H-ferritin antibody (1:600; ab183781, Abcam, USA), anti-GFAP monoclonal anti body (1:500; MAB360, Sigma-Aldrich, USA), anti-Ki67 antibody (1:500, ab15580, Abcam, USA), anti-DCX antibody (1:800; #4604, Cell Signaling Technology, USA), and anti-NeuN antibody (1:500; ab104224, Abcam, USA) were added to the sections, which were then incubated at 4 °C overnight. The corresponding secondary antibodies: Dylight 488 goat anti-mouse IgG (1:200; RS23210, Immunoway, USA) or Alexa Fluor 549 goat anti rabbit IgG (1:200; RS23320, Immunoway, USA). were then added and the samples were incubated at 37 °C for 50 min, after which the sections were stained with DAPI (1:1000) for 4 min. The sections were analyzed using a TG Panoramic Tissue Cell Quantitative Analysis System (Tissue FAXS Plus S, Tissue Gnostics, Austria).

### Inductively coupled plasma mass spectrometry (ICP-MS) of tissue iron content

The mice were perfused with saline to sufficiently remove all blood. The tissues were placed on filter papers to drain the liquid and then transferred to clean microfuge tubes and frozened in liquid nitrogen. After all the tissues were excised, the body weights were recorded. Next, 500 μL 65% nitric acid (68%, J.T. Baker, USA) was added to each tissue sample, which was then stored overnight at room temperature. In ventilation cabinet, the tubes were opened and any excess acid was evaporated for 20 min using in a 90 °C metallic bath. Subsequently, 200 μL H_2_O_2_ was added to each tube, which was incubated in a 70 °C metallic bath for 15 min and then in a 100 °C metallic bath until the remaining acid was completely volatilized. Deionized water was added to bring the final volume to 1 mL, and the total iron content of the wet tissue weight was determined by ICP-MS spectrometer (Agilent7700; Agilent Technologies, Santa Clara, CA, USA).

### RNA isolation and quantitative real-time PCR (qRT-PCR) analysis

Total RNA extraction was performed using TRIzol reagent (Invitrogen) and complementary DNA (cDNA) was synthesized with a PrimeScript™ RT reagent Kit (RR037A, Beijing, China, Takara Biomedical Technology Co., Ltd.). The levels of various target mRNA were assessed by a BioRad real-time PCR system with SYBR Green PCR Master Mix (CW0957L, Cwbio Biotechnology limited company). The primer sequences used were as follows:

StAR forward: 5'-CCGGAGCAGAGTGGTGTCA-3'

StAR reverse: 5'-CAGTGGATGAAGCACCATGC-3'

IL-6 forward: 5'-ACCGCTATGAAGTTCCTCTC-3'

IL-6 reverse: 5'-CTCTGTGAAGTCTCCTCTCC-3'

IL-1β forward: 5'-CCAGCAGGTTATCATCATCATCC-3'

IL-1β reverse: 5'-CTCGCAGCAGCACATCAAC-3'

IL-10 forward: 5'-CAAACAAAGGACCAGCTGGA-3'

IL-10 reverse: 5'-GAGTCCAGCAGACTCAATAC-3'

TGF-β forward: 5'-GACCGCAACAACGCCATCTA-3'

TGF-β reverse: 5'-GGCGTATCAGTGGGGGTCAG-3'

Arg-1 forward: 5'-AGACAGCAGAGGAGGTGAAGAG-3'

Arg-1 reverse: 5'-CGAAG-CAAGCCAAGGTTAAAGC-3'

β-actin forward: 5'-AGGCCCAGAGCAAGAGAGGTA-3'

β-actin reverse: 5'-TCTCCATGTCGTCCCAGTTG-3'

The data were analyzed using the ^ΔΔ^CT method and the target mRNA levels were normalized to those of β-actin (internal control).

### Data processing and statistical analysis

3-month-old Cdh5-Cre transgenic mice were selected as the experimental animals. The *Fpn1*^flox/flox^ mice and *Fpn1*^cdh5^ CKO mice were randomly assigned to Sham or dMCAO groups. G-Power (v3.1.9.2, University of Dusseldorf, Germany) was used for determining reasonable sample size. Due to the large individual variability of mice in behavioral experiments, we correspondingly increased the sample size of mice in each group. The detailed statistical analysis results are shown in Supplemental Table 1, such as the number of animals in each experimental group and power values. Blinding procedures were used as follows: After surgical treatment of mice, a researcher who was blinded to the experimental grouping of the animals performed the neuroethological tests. After the experimental data were acquired, another researcher conducted statistical analysis on the data. GraphPad Prism (v8.0.2) software was used for graphing and analyzing all experimental data. The data were analyzed by Shapiro-Wilk test of normality and variance homogeneity. The data were expressed as the mean ± standard deviation (SD). A two-tailed unpaired Student’s *t*-test was used for comparison between two groups. The data of two variables among the groups were analyzed by two-way ANOVA. Tukey’s (least significant difference) method was used for post-hoc multiple comparison. The detailed statistical analysis results are shown in Supplemental Table 2, such as the number of animals in each experimental group, normality tests, statistic values, *p*-values, mean difference, 95% CIs, and post hoc results for each experiment. A difference of *p* < 0.05 was considered statistically significant [59].

## Supplementary information

Supplemental Table 1

Supplemental Table 2

Original Western Blots bands of Figure 2

Original Western Blots bands of Figure 3

Original Western Blots bands of Figure 5

Original Western Blots bands of Figure 7

Supplementary materials of all full scan the western blots

The reproducibility checklist

## Data Availability

Additional data can be found in the Supplementary materials. The remaining datasets used and analyzed of this study can be obtained on reasonable request from the corresponding author.
