# Supplementary material for: Cdh5-mediated Fpn1 deletion exerts neuroprotective effects during the acute phase and inhibitory effects during the recovery phase of ischemic stroke
Source: Cell Death Dis. 2023 Feb 25;14(2):161. doi: 10.1038/s41419-023-05688-1 (PMC9968354; doi:10.1038/s41419-023-05688-1)
Supplement: Supplementary file 1 — Supplemental Table 1 [file 41419_2023_5688_MOESM1_ESM.docx]

| **Supplemental Table 1 Power analysis of the behavioral experiment data** | | | | | | | |
| --- | --- | --- | --- | --- | --- | --- | --- |
| **Figure** | | | **Number of mice in each panel (n)** | | | | **Power** |
|  |  |  | **Ⅰ** | **Ⅱ** | **Ⅲ** | **Ⅳ** |  |
| Fig. 1C Body Weight | | Before-stroke | 24 | 43 | 26 | 45 | 0.933 |
|  |  | Post-stroke | 24 | 43 | 26 | 45 |  |
| Fig. 1D Neurological score | | Post-stroke | 17 | 27 | 19 | 23 | 1.000 |
| Fig. 1E-H Gait experiment | Fig. 1E LF-stride distance | Before-stroke | 9 | 19 | 7 | 24 | 0.823 |
|  | Fig. 1F LH-stride distance | Post-stroke | 9 | 19 | 7 | 24 | 0.804 |
|  | Fig. 1G RF-stride distance |  |  |  |  |  | 0.890 |
|  | Fig. 1H RH-stride distance |  |  |  |  |  | 0.902 |
| Fig. 1I-L Adhesion removal test | Fig. 1I LF contact time | Before-stroke | 16 | 28 | 19 | 26 | 0.947 |
|  | Fig. 1J LF removal time | Post-stroke | 13 | 26 | 19 | 23 | 0.947 |
|  | Fig. 1K RF contact time* |  |  |  |  |  | 0.737 |
|  | Fig. 1L RF removal time |  |  |  |  |  | 0.824 |
| Fig. 4A Body Weight | | Before-stroke | 24 | 47 | 26 | 55 | 0.934 |
|  |  | Post-stroke | 20 | 18 | 24 | 29 |  |
| Fig. 4B Neurological score | | Post-stroke | 15 | 15 | 19 | 16 | 0.999 |
| Fig. 4C-F Gait experiment | Fig. 4C LF-stride distance | Before-stroke | 26 | 53 | 27 | 50 | 0.908 |
|  | Fig. 4D LH-stride distance | Post-stroke | 19 | 27 | 22 | 26 | 0.863 |
|  | Fig. 4E RF-stride distance |  |  |  |  |  | 0.894 |
|  | Fig. 4F RH-stride distance |  |  |  |  |  | 0.891 |
| Fig. 4G-J Adhesion removal test | Fig. 4G LF contact time | Before-stroke | 15 | 28 | 19 | 26 | 0.921 |
|  | Fig. 4H LF removal time | Post-stroke | 15 | 17 | 19 | 17 | 0.917 |
|  | Fig. 4I RF contact time* |  |  |  |  |  | 0.070 |
|  | Fig. 4J RF removal time |  |  |  |  |  | 0.878 |
| Fig. 6A Body Weight* | | Before-stroke | 24 | 47 | 25 | 55 | 0.349 |
|  |  | Post-stroke | 19 | 16 | 24 | 21 |  |
| Fig. 6B Neurological score | | Post-stroke | 14 | 19 | 17 | 20 | 0.999 |
| Fig. 6C-F Gait experiment | Fig. 6C LF-stride distance | Before-stroke | 26 | 53 | 27 | 50 | 0.917 |
|  | Fig. 6D LH-stride distance | Post-stroke | 16 | 22 | 23 | 22 | 0.898 |
|  | Fig. 6E RF-stride distance |  |  |  |  |  | 0.843 |
|  | Fig. 6F RH-stride distance* |  |  |  |  |  | 0.388 |
| Fig. 6G-J Adhesion removal test | Fig. 6G LF contact time | Before-stroke | 12 | 28 | 19 | 26 | 0.821 |
|  | Fig. 6H LF removal time | Post-stroke | 12 | 21 | 19 | 21 | 0.949 |
|  | Fig. 6I RF contact time* |  |  |  |  |  | 0.458 |
|  | Fig. 6J RF removal time |  |  |  |  |  | 0.914 |
| **Note: Ⅰ**. *Fpn1*^flox/flox^-Sham, **Ⅱ**. *Fpn1*^flox/flox^-dMCAO, **Ⅲ**. *Fpn1*^cdh5^-CKO-Sham, **Ⅳ**. *Fpn1*^cdh5^-CKO-dMCAO.  * A difference of *p* > 0.05 was considered no statistically significant. | | | | | | | |
