# Supplementary material for: Cdh5-mediated Fpn1 deletion exerts neuroprotective effects during the acute phase and inhibitory effects during the recovery phase of ischemic stroke
Source: Cell Death Dis. 2023 Feb 25;14(2):161. doi: 10.1038/s41419-023-05688-1 (PMC9968354; doi:10.1038/s41419-023-05688-1)
Supplement: Supplementary file 2 — Supplemental Table 2 [file 41419_2023_5688_MOESM2_ESM.docx]

**Supplemental Table 2 Statistical summary and analysis methods**

| **Figure reported** | **N** | | | | **Norm. dist?*** | **Statistic** | **Statistic value (df)** | ***p* value** | **Variance source** | ***Post hoc* test** | ***Post hoc p*** | **Mean difference** | **Lower 84% CI** | **Upper 84% CI** |
| --- | --- | --- | --- | --- | --- | --- | --- | --- | --- | --- | --- | --- | --- | --- |
|  | **Ⅰ** | **Ⅱ** | **Ⅲ** | **Ⅳ** |  |  |  |  |  |  |  |  |  |  |
| Fig. 1B Infarction volume ratio |  | 4 |  | 4 | Yes | Unpaired *t* test | *t*(7)*=*3.224 | 0.012 | Difference |  |  | -4.684 | -8.836 | -1.541 |
| Fig. 1C Body Weight |  |  |  |  | Yes | two-way ANOVA | *F* (3, 134) = 0.440 | 0.548 | Interaction |  |  |  |  |  |
| Fig. 1C Body Weight |  |  |  |  |  |  | *F* (1, 134) = 41.070 | <0.001 | Time effect |  |  |  |  |  |
| Fig. 1C Body Weight |  |  |  |  |  |  | *F* (3, 134) = 2.862 | 0.034 | Main effect |  |  |  |  |  |
| Before-stroke | 24 | 43 | 25 | 44 |  |  |  |  | A VS B | Tukey | 0.334 | -1.158 | -2.854 | 0.525 |
|  |  |  |  |  |  |  |  |  | A VS C | Tukey | 0.581 | 0.745 | -1.146 | 2.740 |
|  |  |  |  |  |  |  |  |  | B VS D | Tukey | 0.174 | 1.160 | -0.332 | 2.562 |
|  |  |  |  |  |  |  |  |  | C VS D | Tukey | 0.480 | -0.744 | -2.470 | 0.780 |
| Post-stroke | 24 | 43 | 25 | 44 |  |  |  |  | A VS B | Tukey | 0.840 | -0.388 | -2.183 | 1.385 |
|  |  |  |  |  |  |  |  |  | A VS C | Tukey | 0.403 | 1.065 | -0.816 | 3.060 |
|  |  |  |  |  |  |  |  |  | B VS D | Tukey | 0.454 | 0.644 | -0.646 | 2.246 |
|  |  |  |  |  |  |  |  |  | C VS D | Tukey | 0.605 | -0.620 | -2.444 | 1.014 |
| Fig. 1D Neurological score | 16 | 26 | 18 | 23 | Yes | two-way ANOVA | *F*(1, 72)=7.725 | 0.004 | Interaction |  |  |  |  |  |
| Fig. 1D Neurological score |  |  |  |  |  |  | *F*(1, 72)=21.880 | <0.001 | Main effect (gene) |  |  |  |  |  |
| Fig. 1D Neurological score |  |  |  |  |  |  | *F*(1, 72)=144.300 | <0.001 | Main effect  (treatment-dMCAO) |  |  |  |  |  |
|  |  |  |  |  |  |  |  |  | A VS B | Tukey | <0.001 | -3.452 | -4.443 | -2.571 |
|  |  |  |  |  |  |  |  |  | A VS C | Tukey | 0.563 | 0.408 | -0.442 | 1.348 |
|  |  |  |  |  |  |  |  |  | B VS D | Tukey | <0.001 | -2.140 | -3.032 | -1.256 |
|  |  |  |  |  |  |  |  |  | C VS D | Tukey | <0.001 | 1.721 | 1.014 | 2.528 |
| Fig. 1E LF-stride distance |  |  |  |  | Yes | two-way ANOVA | *F*(3, 44)=2.182 | 0.088 | Interaction |  |  |  |  |  |
| Fig. 1E LF-stride distance |  |  |  |  |  |  | *F*(1, 44)=0.220 | 0.541 | Time effect |  |  |  |  |  |
| Fig. 1E LF-stride distance |  |  |  |  |  |  | *F*(3, 44)=3.245 | 0.027 | Main effect |  |  |  |  |  |
| Before-stroke | 8 | 18 | 6 | 24 |  |  |  |  | A VS B | Tukey | 0.426 | 0.403 | -0.347 | 1.354 |
|  |  |  |  |  |  |  |  |  | A VS C | Tukey | 0.328 | 0.587 | -0.364 | 1.661 |
|  |  |  |  |  |  |  |  |  | B VS D | Tukey | 0.853 | 0.121 | -0.433 | 0.664 |
|  |  |  |  |  |  |  |  |  | C VS D | Tukey | 0.886 | -0.064 | -0.878 | 0.738 |
| Post-stroke | 8 | 18 | 6 | 24 |  |  |  |  | A VS B | Tukey | 0.033 | 0.814 | 0.044 | 1.665 |
|  |  |  |  |  |  |  |  |  | A VS C | Tukey | 0.703 | 0.361 | -0.601 | 1.444 |
|  |  |  |  |  |  |  |  |  | B VS D | Tukey | 0.821 | 0.148 | -0.484 | 0.712 |
|  |  |  |  |  |  |  |  |  | C VS D | Tukey | 0.183 | 0.602 | -0.212 | 1.515 |
| Fig. 1F LH-stride distance |  |  |  |  | Yes | two-way ANOVA | *F*(3, 44)=2.541 | 0.047 | Interaction |  |  |  |  |  |
| Fig. 1F LH-stride distance |  |  |  |  |  |  | *F*(1, 44)=0.447 | 0.405 | Time effect |  |  |  |  |  |
| Fig. 1F LH-stride distance |  |  |  |  |  |  | *F*(3, 44)=3.434 | 0.023 | Main effect |  |  |  |  |  |
| Before-stroke | 8 | 18 | 6 | 24 |  |  |  |  | A VS B | Tukey | 0.244 | 0.430 | -0.215 | 1.266 |
|  |  |  |  |  |  |  |  |  | A VS C | Tukey | 0.058 | 0.774 | -0.046 | 1.713 |
|  |  |  |  |  |  |  |  |  | B VS D | Tukey | 1.000 | 0.011 | -0.445 | 0.466 |
|  |  |  |  |  |  |  |  |  | C VS D | Tukey | 0.563 | -0.343 | -1.134 | 0.440 |
| Post-stroke | 8 | 18 | 6 | 24 |  |  |  |  | A VS B | Tukey | 0.018 | 0.742 | 0.104 | 1.487 |
|  |  |  |  |  |  |  |  |  | A VS C | Tukey | 0.472 | 0.444 | -0.465 | 1.374 |
|  |  |  |  |  |  |  |  |  | B VS D | Tukey | 0.883 | 0.051 | -0.405 | 0.527 |
|  |  |  |  |  |  |  |  |  | C VS D | Tukey | 0.435 | 0.448 | -0.334 | 1.241 |
| Fig. 1G RF-stride distance |  |  |  |  | Yes | two-way ANOVA | *F*(3, 44)=2.574 | 0.045 | Interaction |  |  |  |  |  |
| Fig. 1G RF-stride distance |  |  |  |  |  |  | *F*(1, 44)=0.050 | 0.707 | Time effect |  |  |  |  |  |
| Fig. 1G RF-stride distance |  |  |  |  |  |  | *F*(3, 44)=3.520 | 0.018 | Main effect |  |  |  |  |  |
| Before-stroke | 8 | 18 | 6 | 24 |  |  |  |  | A VS B | Tukey | 0.481 | 0.473 | -0.404 | 1.361 |
|  |  |  |  |  |  |  |  |  | A VS C | Tukey | 0.182 | 0.740 | -0.245 | 1.845 |
|  |  |  |  |  |  |  |  |  | B VS D | Tukey | 0.728 | 0.220 | -0.444 | 0.784 |
|  |  |  |  |  |  |  |  |  | C VS D | Tukey | 0.866 | -0.146 | -1.080 | 0.685 |
| Post-stroke | 8 | 18 | 6 | 24 |  |  |  |  | A VS B | Tukey | 0.047 | 0.784 | 0.006 | 1.673 |
|  |  |  |  |  |  |  |  |  | A VS C | Tukey | 0.580 | 0.455 | -0.540 | 1.462 |
|  |  |  |  |  |  |  |  |  | B VS D | Tukey | 0.544 | 0.303 | -0.361 | 0.866 |
|  |  |  |  |  |  |  |  |  | C VS D | Tukey | 0.174 | 0.632 | -0.210 | 1.564 |
| Fig. 1H RH-stride distance |  |  |  |  | Yes | two-way ANOVA | *F* (3, 44) = 0.623 | 0.442 | Interaction |  |  |  |  |  |
| Fig. 1H RH-stride distance |  |  |  |  |  |  | *F* (1, 44) = 0.025 | 0.763 | Time effect |  |  |  |  |  |
| Fig. 1H RH-stride distance |  |  |  |  |  |  | *F* (3, 44) = 4.217 | 0.008 | Main effect |  |  |  |  |  |
|  |  |  |  |  |  |  |  |  |  |  |  |  |  |  |
| Before-stroke | 8 | 18 | 6 | 24 |  |  |  |  | A VS B | Tukey | 0.228 | 0.464 | -0.210 | 1.350 |
|  |  |  |  |  |  |  |  |  | A VS C | Tukey | 0.305 | 0.543 | -0.324 | 1.531 |
|  |  |  |  |  |  |  |  |  | B VS D | Tukey | 0.805 | 0.144 | -0.441 | 0.640 |
|  |  |  |  |  |  |  |  |  | C VS D | Tukey | 0.884 | 0.065 | -0.646 | 0.810 |
| Post-stroke | 8 | 18 | 6 | 24 |  |  |  |  | A VS B | Tukey | 0.046 | 0.682 | 0.006 | 1.466 |
|  |  |  |  |  |  |  |  |  | A VS C | Tukey | 0.474 | 0.434 | -0.443 | 1.413 |
|  |  |  |  |  |  |  |  |  | B VS D | Tukey | 0.623 | 0.238 | -0.346 | 0.734 |
|  |  |  |  |  |  |  |  |  | C VS D | Tukey | 0.411 | 0.485 | -0.337 | 1.328 |
| Fig. 1I LF contact time |  |  |  |  | Yes | two-way ANOVA | *F* (3, 64) = 18.040 | <0.001 | Interaction |  |  |  |  |  |
| Fig. 1I LF contact time |  |  |  |  |  |  | *F* (1, 64) = 30.200 | <0.001 | Time effect |  |  |  |  |  |
| Fig. 1I LF contact Time |  |  |  |  |  |  | *F* (3, 76) = 7.523 | <0.001 | Main effect |  |  |  |  |  |
| Before-stroke | 15 | 27 | 18 | 25 |  |  |  |  |  | Tukey | 0.541 | 4.886 | -5.006 | 15.000 |
|  |  |  |  |  |  |  |  |  | A VS C | Tukey | 0.817 | 2.847 | -7.861 | 14.760 |
|  |  |  |  |  |  |  |  |  | B VS D | Tukey | 0.547 | -4.244 | -13.720 | 4.325 |
|  |  |  |  |  |  |  |  |  | C VS D | Tukey | 0.840 | -2.186 | -12.710 | 7.414 |
| Post-stroke | 13 | 25 | 18 | 23 |  |  |  |  | A VS B | Tukey | <0.001 | -27.740 | -40.630 | -15.860 |
|  |  |  |  |  |  |  |  |  | A VS C | Tukey | 0.875 | 1.551 | -10.840 | 14.260 |
|  |  |  |  |  |  |  |  |  | B VS D | Tukey | 0.014 | 11.650 | 1.627 | 21.700 |
|  |  |  |  |  |  |  |  |  | C VS D | Tukey | <0.001 | -17.640 | -28.530 | -6.754 |
| Fig. 1J LF removal time |  |  |  |  | Yes | two-way ANOVA | *F* (3, 65) = 38.410 | <0.001 | Interaction |  |  |  |  |  |
| Fig. 1J LF removal time |  |  |  |  |  |  | *F* (1, 65) = 112.100 | <0.001 | Time effect |  |  |  |  |  |
| Fig. 1J LF removal time |  |  |  |  |  |  | *F* (3, 75) = 24.540 | <0.001 | Main effect |  |  |  |  |  |
| Before-stroke | 15 | 27 | 18 | 25 |  |  |  |  | A VS B | Tukey | 1.000 | 0.514 | -20.440 | 21.570 |
|  |  |  |  |  |  |  |  |  | A VS C | Tukey | 0.888 | -1.367 | -24.180 | 21.430 |
|  |  |  |  |  |  |  |  |  | B VS D | Tukey | 0.821 | -4.463 | -22.660 | 13.730 |
|  |  |  |  |  |  |  |  |  | C VS D | Tukey | 0.878 | -2.470 | -22.660 | 16.710 |
| Post-stroke | 13 | 25 | 18 | 23 |  |  |  |  | A VS B | Tukey | <0.001 | -77.180 | -110.800 | -54.440 |
|  |  |  |  |  |  |  |  |  | A VS C | Tukey | 0.881 | -2.644 | -25.770 | 21.360 |
|  |  |  |  |  |  |  |  |  | B VS D | Tukey | 0.025 | 20.840 | 1.654 | 40.140 |
|  |  |  |  |  |  |  |  |  | C VS D | Tukey | <0.001 | -54.470 | -74.280 | -43.550 |
| Fig. 1K RF contact time |  |  |  |  | Yes | two-way ANOVA | *F* (3, 65) = 3.540 | 0.015 | Interaction |  |  |  |  |  |
| Fig. 1K RF contact time |  |  |  |  |  |  | *F* (1, 65) = 0.4656 | 0.440 | Time effect |  |  |  |  |  |
| Fig. 1K RF contact time |  |  |  |  |  |  | *F* (3, 75) = 2.143 | 0.100 | Main effect |  |  |  |  |  |
| Before-stroke | 15 | 27 | 18 | 25 |  |  |  |  |  |  |  |  |  |  |
| Post-stroke | 13 | 25 | 18 | 23 |  |  |  |  |  |  |  |  |  |  |
| Fig. 1L RF removal time |  |  |  |  | Yes | two-way ANOVA | *F* (3, 65) = 12.120 | <0.001 | Interaction |  |  |  |  |  |
| Fig. 1L RF removal time |  |  |  |  |  |  | *F* (1, 65) = 33.410 | <0.001 | Time effect |  |  |  |  |  |
| Fig. 1L RF removal time |  |  |  |  |  |  | *F* (3, 75) = 8.668 | <0.001 | Main effect |  |  |  |  |  |
| Before-stroke | 15 | 27 | 18 | 25 |  |  |  |  | A VS B | Tukey | 0.845 | 4.337 | -16.500 | 25.270 |
|  |  |  |  |  |  |  |  |  | A VS C | Tukey | 0.834 | 4.418 | -17.340 | 28.170 |
|  |  |  |  |  |  |  |  |  | B VS D | Tukey | 0.813 | -4.728 | -23.800 | 14.240 |
|  |  |  |  |  |  |  |  |  | C VS D | Tukey | 0.776 | -4.810 | -26.040 | 14.220 |
| Post-stroke | 13 | 24 | 18 | 20 |  |  |  |  | A VS B | Tukey | <0.001 | -47.710 | -62.460 | -24.040 |
|  |  |  |  |  |  |  |  |  | A VS C | Tukey | 0.547 | 11.330 | -13.750 | 35.410 |
|  |  |  |  |  |  |  |  |  | B VS D | Tukey | 0.003 | 26.310 | 6.273 | 46.340 |
|  |  |  |  |  |  |  |  |  | C VS D | Tukey | <0.001 | -32.720 | -44.420 | -11.120 |
| Fig. 2B FtL expression | 5 | 5 | 5 | 5 | Yes | two-way ANOVA | *F*(1, 20)=0.812 | 0.341 | Interaction |  |  |  |  |  |
| Fig. 2B FtL expression |  |  |  |  |  |  | *F*(1, 20)=34.313 | <0.001 | Main effect (gene) |  |  |  |  |  |
| Fig. 2B FtL expression |  |  |  |  |  |  | *F*(1, 20)=6.384 | 0.013 | Main effect  (treatment-dMCAO) |  |  |  |  |  |
|  |  |  |  |  |  |  |  |  | A VS B | Tukey | 0.064 | -0.784 | -1.745 | 0.058 |
|  |  |  |  |  |  |  |  |  | A VS C | Tukey | 0.012 | 1.182 | 0.230 | 2.144 |
|  |  |  |  |  |  |  |  |  | B VS D | Tukey | <0.001 | 1.546 | 0.584 | 2.518 |
|  |  |  |  |  |  |  |  |  | C VS D | Tukey | 0.504 | -0.428 | -1.382 | 0.434 |
| Fig. 2C TfR1 expression | 5 | 5 | 5 | 5 | Yes | two-way ANOVA | *F*(1, 20)=0.148 | 0.584 | Interaction |  |  |  |  |  |
| Fig. 2C TfR1 expression |  |  |  |  |  |  | *F*(1, 20)=22.685 | <0.001 | Main effect (gene) |  |  |  |  |  |
| Fig. 2C TfR1 expression |  |  |  |  |  |  | *F*(1, 20)=0.021 | 0.776 | Main effect  (treatment-dMCAO) |  |  |  |  |  |
|  |  |  |  |  |  |  |  |  | A VS B | Tukey | 0.887 | -0.044 | -0.804 | 0.685 |
|  |  |  |  |  |  |  |  |  | A VS C | Tukey | 0.007 | -1.111 | -1.851 | -0.251 |
|  |  |  |  |  |  |  |  |  | B VS D | Tukey | 0.026 | -0.840 | -1.680 | -0.080 |
|  |  |  |  |  |  |  |  |  | C VS D | Tukey | 0.870 | 0.116 | -0.634 | 0.856 |
| Fig. 2D FtH expression | 5 | 5 | 5 | 5 | Yes | two-way ANOVA | *F*(1, 20)=0.076 | 0.661 | Interaction |  |  |  |  |  |
| Fig. 2D FtH expression |  |  |  |  |  |  | *F*(1, 20)=26.873 | <0.001 | Main effect (gene) |  |  |  |  |  |
| Fig. 2D FtH expression |  |  |  |  |  |  | *F*(1, 20)=5.774 | 0.015 | Main effect  (treatment-dMCAO) |  |  |  |  |  |
|  |  |  |  |  |  |  |  |  | A VS B | Tukey | 0.366 | -0.384 | -1.053 | 0.265 |
|  |  |  |  |  |  |  |  |  | A VS C | Tukey | 0.004 | 0.844 | 0.264 | 1.513 |
|  |  |  |  |  |  |  |  |  | B VS D | Tukey | <0.001 | 0.744 | 0.164 | 1.413 |
|  |  |  |  |  |  |  |  |  | C VS D | Tukey | 0.255 | -0.484 | -1.153 | 0.165 |
| Fig. 2F FPN1 expression | 5 | 5 | 5 | 5 | Yes | two-way ANOVA | *F*(1, 20)=0.854 | 0.337 | Interaction |  |  |  |  |  |
| Fig. 2F FPN1 expression |  |  |  |  |  |  | *F*(1, 20)=0.162 | 0.573 | Main effect (gene) |  |  |  |  |  |
| Fig. 2F FPN1 expression |  |  |  |  |  |  | *F*(1, 20)=0.020 | 0.780 | Main effect  (treatment-dMCAO) |  |  |  |  |  |
| Fig. 2G Bcl2/Bax expression | 5 | 5 | 5 | 5 | Yes | two-way ANOVA | *F*(1, 20)=1.116 | 0.303 | Interaction |  |  |  |  |  |
| Fig. 2G Bcl2/Bax expression |  |  |  |  |  |  | *F*(1, 20)=34.627 | <0.001 | Main effect (gene) |  |  |  |  |  |
| Fig. 2G Bcl2/Bax expression |  |  |  |  |  |  | *F*(1, 20)=0.244 | 0.518 | Main effect  (treatment-dMCAO) |  |  |  |  |  |
|  |  |  |  |  |  |  |  |  | A VS B | Tukey | 0.868 | 0.042 | -0.322 | 0.425 |
|  |  |  |  |  |  |  |  |  | A VS C | Tukey | 0.013 | -0.446 | -0.731 | -0.073 |
|  |  |  |  |  |  |  |  |  | B VS D | Tukey | <0.001 | -0.546 | -1.031 | -0.273 |
|  |  |  |  |  |  |  |  |  | C VS D | Tukey | 0.581 | -0.147 | -0.422 | 0.226 |
| Fig. 2H p-Erk1/2 expression | 5 | 5 | 5 | 5 | Yes | two-way ANOVA | *F*(1, 20)=1.564 | 0.210 | Interaction |  |  |  |  |  |
| Fig. 2H p-Erk1/2 expression |  |  |  |  |  |  | *F*(1, 20)=8.123 | 0.006 | Main effect (gene) |  |  |  |  |  |
| Fig. 2H p-Erk1/2 expression |  |  |  |  |  |  | *F*(1, 20)=13.120 | 0.002 | Main effect  (treatment-dMCAO) |  |  |  |  |  |
|  |  |  |  |  |  |  |  |  | A VS B | Tukey | 0.366 | -0.162 | -0.454 | 0.120 |
|  |  |  |  |  |  |  |  |  | A VS C | Tukey | 0.521 | -0.126 | -0.420 | 0.154 |
|  |  |  |  |  |  |  |  |  | B VS D | Tukey | 0.030 | -0.317 | -0.511 | -0.025 |
|  |  |  |  |  |  |  |  |  | C VS D | Tukey | 0.012 | -0.353 | -0.544 | -0.061 |
| Fig. 3B ACSL4 expression | 5 | 5 | 5 | 5 | Yes | two-way ANOVA | *F*(1, 20)=2.240 | 0.140 | Interaction |  |  |  |  |  |
| Fig. 3B ACSL4 expression |  |  |  |  |  |  | *F*(1, 20)=12.558 | 0.002 | Main effect (gene) |  |  |  |  |  |
| Fig. 3B ACSL4 expression |  |  |  |  |  |  | *F*(1, 20)=0.040 | 0.724 | Main effect  (treatment-dMCAO) |  |  |  |  |  |
|  |  |  |  |  |  |  |  |  | A VS B | Tukey | 0.704 | -0.068 | -0.324 | 0.155 |
|  |  |  |  |  |  |  |  |  | A VS C | Tukey | 0.470 | 0.127 | -0.116 | 0.363 |
|  |  |  |  |  |  |  |  |  | B VS D | Tukey | 0.008 | 0.313 | 0.057 | 0.447 |
|  |  |  |  |  |  |  |  |  | C VS D | Tukey | 0.524 | 0.106 | -0.138 | 0.342 |
| Fig. 3C GPX4 expression | 5 | 5 | 5 | 5 | Yes | two-way ANOVA | *F*(1, 20)=5.166 | 0.022 | Interaction |  |  |  |  |  |
| Fig. 3C GPX4 expression |  |  |  |  |  |  | *F*(1, 20)=7.672 | 0.007 | Main effect (gene) |  |  |  |  |  |
| Fig. 3C GPX4 expression |  |  |  |  |  |  | *F*(1, 20)=2.478 | 0.123 | Main effect  (treatment-dMCAO) |  |  |  |  |  |
|  |  |  |  |  |  |  |  |  | A VS B | Tukey | 0.041 | 0.142 | 0.004 | 0.300 |
|  |  |  |  |  |  |  |  |  | A VS C | Tukey | 0.875 | -0.017 | -0.154 | 0.130 |
|  |  |  |  |  |  |  |  |  | B VS D | Tukey | 0.004 | -0.203 | -0.340 | -0.045 |
|  |  |  |  |  |  |  |  |  | C VS D | Tukey | 0.824 | -0.033 | -0.170 | 0.114 |
| Fig. 3D Total Nrf2 expression | 5 | 5 | 5 | 5 | Yes | two-way ANOVA | *F*(1, 20)=12.140 | 0.002 | Interaction |  |  |  |  |  |
| Fig. 3D Total Nrf2 expression |  |  |  |  |  |  | *F*(1, 20)=147.761 | <0.001 | Main effect (gene) |  |  |  |  |  |
| Fig. 3D Total Nrf2 expression |  |  |  |  |  |  | *F*(1, 20)=33.731 | <0.001 | Main effect  (treatment-dMCAO) |  |  |  |  |  |
|  |  |  |  |  |  |  |  |  | A VS B | Tukey | 0.365 | -0.103 | -0.268 | 0.062 |
|  |  |  |  |  |  |  |  |  | A VS C | Tukey | <0.001 | -0.376 | -0.452 | -0.211 |
|  |  |  |  |  |  |  |  |  | B VS D | Tukey | <0.001 | -0.585 | -0.761 | -0.420 |
|  |  |  |  |  |  |  |  |  | C VS D | Tukey | <0.001 | -0.413 | -0.477 | -0.236 |
| Fig. 3E Cytoplasmic Nrf2 expression | 5 | 5 | 5 | 5 | Yes | two-way ANOVA | *F*(1, 20)=0.645 | 0.384 | Interaction |  |  |  |  |  |
| Fig. 3E Cytoplasmic Nrf2 expression |  |  |  |  |  |  | *F*(1, 20)=12.862 | 0.002 | Main effect (gene) |  |  |  |  |  |
| Fig. 3E Cytoplasmic Nrf2 expression |  |  |  |  |  |  | *F*(1, 20)=42.351 | <0.001 | Main effect  (treatment-dMCAO) |  |  |  |  |  |
|  |  |  |  |  |  |  |  |  | A VS B | Tukey | 0.004 | -0.180 | -0.324 | -0.046 |
|  |  |  |  |  |  |  |  |  | A VS C | Tukey | 0.246 | -0.082 | -0.225 | 0.041 |
|  |  |  |  |  |  |  |  |  | B VS D | Tukey | 0.023 | -0.141 | -0.274 | -0.016 |
|  |  |  |  |  |  |  |  |  | C VS D | Tukey | <0.001 | -0.248 | -0.373 | -0.115 |
| Fig. 3F Nuclear Nrf2 expression | 5 | 5 | 5 | 5 | Yes | two-way ANOVA | *F*(1, 20)=1.156 | 0.283 | Interaction |  |  |  |  |  |
| Fig. 3F Nuclear Nrf2 expression |  |  |  |  |  |  | *F*(1, 20)=4.251 | 0.033 | Main effect (gene) |  |  |  |  |  |
| Fig. 3F Nuclear Nrf2 expression |  |  |  |  |  |  | *F*(1, 20)=35.500 | <0.001 | Main effect  (treatment-dMCAO) |  |  |  |  |  |
|  |  |  |  |  |  |  |  |  | A VS B | Tukey | <0.001 | -1.565 | -2.505 | -0.644 |
|  |  |  |  |  |  |  |  |  | A VS C | Tukey | 0.112 | -0.683 | -1.623 | 0.136 |
|  |  |  |  |  |  |  |  |  | B VS D | Tukey | 0.725 | -0.274 | -1.214 | 0.544 |
|  |  |  |  |  |  |  |  |  | C VS D | Tukey | 0.011 | -1.157 | -2.087 | -0.237 |
| Fig. 3G HO1 expression | 5 | 5 | 5 | 5 | Yes | two-way ANOVA | *F*(1, 20)=5.438 | 0.018 | Interaction |  |  |  |  |  |
| Fig. 3G HO1 expression |  |  |  |  |  |  | *F*(1, 20)=0.132 | 0.620 | Main effect (gene) |  |  |  |  |  |
| Fig. 3G HO1 expression |  |  |  |  |  |  | *F(*1, 20)=6.347 | 0.013 | Main effect  (treatment-dMCAO) |  |  |  |  |  |
|  |  |  |  |  |  |  |  |  | A VS B | Tukey | 1.000 | 0.012 | -0.280 | 0.314 |
|  |  |  |  |  |  |  |  |  | A VS C | Tukey | 0.187 | -0.223 | -0.424 | 0.068 |
|  |  |  |  |  |  |  |  |  | B VS D | Tukey | 0.427 | 0.156 | -0.134 | 0.458 |
|  |  |  |  |  |  |  |  |  | C VS D | Tukey | 0.006 | 0.402 | 0.100 | 0.604 |
| Fig. 3H 4HNE expression | 5 | 5 | 5 | 5 | Yes | two-way ANOVA | *F*(1, 20)=6.827 | 0.011 | Interaction |  |  |  |  |  |
| Fig. 3H 4HNE expression |  |  |  |  |  |  | *F*(1, 20)=4.345 | 0.032 | Main effect (gene) |  |  |  |  |  |
| Fig. 3H 4HNE expression |  |  |  |  |  |  | *F*(1, 20)=4.367 | 0.048 | Main effect  (treatment-dMCAO) |  |  |  |  |  |
|  |  |  |  |  |  |  |  |  | A VS B | Tukey | 0.844 | -0.044 | -0.276 | 0.188 |
|  |  |  |  |  |  |  |  |  | A VS C | Tukey | 0.874 | -0.031 | -0.264 | 0.212 |
|  |  |  |  |  |  |  |  |  | B VS D | Tukey | 0.007 | 0.314 | 0.062 | 0.447 |
|  |  |  |  |  |  |  |  |  | C VS D | Tukey | 0.012 | 0.301 | 0.047 | 0.444 |
| Fig. 3I IL-6 mRNA level | 5 | 5 | 5 | 5 | Yes | two-way ANOVA | *F*(1, 20)=6.040 | 0.014 | Interaction |  |  |  |  |  |
| Fig. 3I IL-6 mRNA level |  |  |  |  |  |  | *F*(1, 20)=7.015 | 0.010 | Main effect (gene) |  |  |  |  |  |
| Fig. 3I IL-6 mRNA level |  |  |  |  |  |  | *F*(1, 20)=51.06 | <0.001 | Main effect  (treatment-dMCAO) |  |  |  |  |  |
|  |  |  |  |  |  |  |  |  | A VS B | Tukey | <0.001 | -61.310 | -87.260 | -44.340 |
|  |  |  |  |  |  |  |  |  | A VS C | Tukey | 0.888 | 1.212 | -24.640 | 27.170 |
|  |  |  |  |  |  |  |  |  | B VS D | Tukey | 0.004 | 36.350 | 10.400 | 54.330 |
|  |  |  |  |  |  |  |  |  | C VS D | Tukey | 0.007 | -34.150 | -52.120 | -7.185 |
| Fig. 3J IL-1β mRNA level | 5 | 5 | 5 | 5 | Yes | two-way ANOVA | *F*(1, 20)=0.118 | 0.633 | Interaction |  |  |  |  |  |
| Fig. 3J IL-1β mRNA level |  |  |  |  |  |  | *F*(1, 20)=0.152 | 0.581 | Main effect (gene) |  |  |  |  |  |
| Fig. 3J IL-1β mRNA level |  |  |  |  |  |  | *F*(1, 20)=47.72 | <0.001 | Main effect  (treatment-dMCAO) |  |  |  |  |  |
|  |  |  |  |  |  |  |  |  | A VS B | Tukey | <0.001 | -20.570 | -31.740 | -8.415 |
|  |  |  |  |  |  |  |  |  | A VS C | Tukey | >0.888 | 0.152 | -11.000 | 11.330 |
|  |  |  |  |  |  |  |  |  | B VS D | Tukey | 0.841 | 2.110 | -8.043 | 13.260 |
|  |  |  |  |  |  |  |  |  | C VS D | Tukey | <0.001 | -17.630 | -28.780 | -6.457 |
| Fig. 3K IL-10 mRNA level | 5 | 5 | 5 | 5 | Yes | two-way ANOVA | *F*(1, 20)=0.114 | 0.637 | Interaction |  |  |  |  |  |
| Fig. 3K IL-10 mRNA level |  |  |  |  |  |  | *F*(1, 20)=11.200 | 0.003 | Main effect (gene) |  |  |  |  |  |
| Fig. 3K IL-10 mRNA level |  |  |  |  |  |  | *F*(1, 20)=5.423 | 0.018 | Main effect  (treatment-dMCAO) |  |  |  |  |  |
|  |  |  |  |  |  |  |  |  | A VS B | Tukey | 0.418 | -0.232 | -0.544 | 0.172 |
|  |  |  |  |  |  |  |  |  | A VS C | Tukey | 0.168 | -0.314 | -0.627 | 0.088 |
|  |  |  |  |  |  |  |  |  | B VS D | Tukey | 0.064 | -0.374 | -0.688 | 0.028 |
|  |  |  |  |  |  |  |  |  | C VS D | Tukey | 0.204 | -0.303 | -0.615 | 0.111 |
| Fig. 3L TGF-β mRNA level | 5 | 5 | 5 | 5 | Yes | two-way ANOVA | *F*(1, 20)=1.153 | 0.284 | Interaction |  |  |  |  |  |
| Fig. 3L TGF-β mRNA level |  |  |  |  |  |  | *F*(1, 20)=0.564 | 0.421 | Main effect (gene) |  |  |  |  |  |
| Fig. 3L TGF-β mRNA level |  |  |  |  |  |  | *F*(1, 20)=4.604 | 0.026 | Main effect  (treatment-dMCAO) |  |  |  |  |  |
|  |  |  |  |  |  |  |  |  | A VS B | Tukey | 0.088 | 0.380 | -0.044 | 0.735 |
|  |  |  |  |  |  |  |  |  | A VS C | Tukey | 0.446 | 0.214 | -0.232 | 0.550 |
|  |  |  |  |  |  |  |  |  | B VS D | Tukey | 0.887 | -0.028 | -0.464 | 0.416 |
|  |  |  |  |  |  |  |  |  | C VS D | Tukey | 0.681 | 0.147 | -0.287 | 0.483 |
| Fig. 3M Arg-1 mRNA level | 5 | 5 | 5 | 5 | Yes | two-way ANOVA | *F*(1, 20)=2.144 | 0.148 | Interaction |  |  |  |  |  |
| Fig. 3M Arg-1 mRNA level |  |  |  |  |  |  | *F*(1, 20)=4.544 | 0.027 | Main effect (gene) |  |  |  |  |  |
| Fig. 3M Arg-1 mRNA level |  |  |  |  |  |  | *F*(1, 20)=26.43 | <0.001 | Main effect  (treatment-dMCAO) |  |  |  |  |  |
|  |  |  |  |  |  |  |  |  | A VS B | Tukey | 0.054 | 0.627 | -0.035 | 1.482 |
|  |  |  |  |  |  |  |  |  | A VS C | Tukey | 0.048 | -0.642 | -1.404 | 0.022 |
|  |  |  |  |  |  |  |  |  | B VS D | Tukey | 0.815 | -0.165 | -0.840 | 0.477 |
|  |  |  |  |  |  |  |  |  | C VS D | Tukey | <0.001 | 1.283 | 0.428 | 2.046 |
| Fig. 4A Body Weight |  |  |  |  | Yes | two-way ANOVA | *F* (3, 234) = 3.274 | 0.022 | Interaction |  |  |  |  |  |
| Fig. 4A Body Weight |  |  |  |  |  |  | *F* (1, 234) = 14.84 | <0.001 | Time effect |  |  |  |  |  |
| Fig. 4A Body Weight |  |  |  |  |  |  | *F* (3, 234) = 2.428 | 0.047 | Main effect |  |  |  |  |  |
| Before-stroke | 24 | 46 | 25 | 44 |  |  |  |  | A VS B | Tukey | 0.416 | -0.785 | -2.472 | 0.682 |
|  |  |  |  |  |  |  |  |  | A VS C | Tukey | 0.548 | 0.745 | -1.046 | 2.640 |
|  |  |  |  |  |  |  |  |  | B VS D | Tukey | 0.144 | 1.077 | -0.247 | 2.423 |
|  |  |  |  |  |  |  |  |  | C VS D | Tukey | 0.614 | -0.544 | -2.244 | 0.845 |
| Post-stroke | 20 | 17 | 24 | 28 |  |  |  |  | A VS B | Tukey | 0.718 | 0.636 | -1.446 | 2.822 |
|  |  |  |  |  |  |  |  |  | A VS C | Tukey | >0.888 | -0.011 | -2.046 | 2.024 |
|  |  |  |  |  |  |  |  |  | B VS D | Tukey | 0.430 | 1.171 | -0.736 | 3.188 |
|  |  |  |  |  |  |  |  |  | C VS D | Tukey | 0.037 | 1.828 | 0.064 | 3.674 |
| Fig. 4B Neurological score | 14 | 14 | 18 | 15 | Yes | two-way ANOVA | *F* (1, 51) = 14.140 | <0.001 | Interaction |  |  |  |  |  |
| Fig. 4B Neurological score |  |  |  |  |  |  | *F* (1, 51) = 45.110 | <0.001 | Main effect (gene) |  |  |  |  |  |
| Fig. 4B Neurological score |  |  |  |  |  |  | *F* (1, 51) = 12.350 | <0.001 | Main effect  (treatment-dMCAO) |  |  |  |  |  |
|  |  |  |  |  |  |  |  |  | A VS C | Tukey | 0.887 | 0.140 | -1.844 | 2.234 |
|  |  |  |  |  |  |  |  |  | A VS B | Tukey | 0.164 | -1.633 | -3.846 | 0.470 |
|  |  |  |  |  |  |  |  |  | B VS D | Tukey | <0.001 | -5.035 | -7.083 | -3.868 |
|  |  |  |  |  |  |  |  |  | C VS D | Tukey | <0.001 | -4.152 | -5.341 | -1.874 |
| Fig. 4C LF stride distance |  |  |  |  | Yes | two-way ANOVA | *F* (3, 242) = 0.703 | 0.483 | Interaction |  |  |  |  |  |
| Fig. 4C LF stride distance |  |  |  |  |  |  | *F* (1, 242) = 26.070 | <0.001 | Time effect |  |  |  |  |  |
| Fig. 4C LF stride distance |  |  |  |  |  |  | *F* (3, 242) = 5.633 | <0.001 | Main effect |  |  |  |  |  |
| Before-stroke | 25 | 43 | 26 | 40 |  |  |  |  | A VS B | Tukey | 0.631 | 0.157 | -0.244 | 0.480 |
|  |  |  |  |  |  |  |  |  | A VS C | Tukey | 0.067 | 0.440 | -0.034 | 0.834 |
|  |  |  |  |  |  |  |  |  | B VS D | Tukey | 0.054 | 0.334 | -0.013 | 0.571 |
|  |  |  |  |  |  |  |  |  | C VS D | Tukey | 0.878 | 0.042 | -0.358 | 0.462 |
| Post-stroke | 18 | 26 | 22 | 25 |  |  |  |  | A VS B | Tukey | >0.888 | 0.008 | -0.417 | 0.435 |
|  |  |  |  |  |  |  |  |  | A VS C | Tukey | 0.844 | 0.111 | -0.441 | 0.552 |
|  |  |  |  |  |  |  |  |  | B VS D | Tukey | 0.032 | 0.414 | 0.032 | 0.888 |
|  |  |  |  |  |  |  |  |  | C VS D | Tukey | 0.145 | 0.414 | -0.085 | 0.824 |
| Fig. 4D LH stride distance |  |  |  |  | Yes | two-way ANOVA | *F* (3, 74) = 2.130 | 0.103 | Interaction |  |  |  |  |  |
| Fig. 4D LH stride distance |  |  |  |  |  |  | *F* (1, 74) = 7.347 | 0.004 | Time effect |  |  |  |  |  |
| Fig. 4D LH stride distance |  |  |  |  |  |  | *F* (3, 147) = 6.211 | <0.001 | Main effect |  |  |  |  |  |
| Before-stroke | 25 | 43 | 26 | 40 |  |  |  |  | A VS B | Tukey | >0.888 | -0.002 | -0.354 | 0.350 |
|  |  |  |  |  |  |  |  |  | A VS C | Tukey | 0.282 | 0.274 | -0.132 | 0.600 |
|  |  |  |  |  |  |  |  |  | B VS D | Tukey | 0.123 | 0.244 | -0.043 | 0.443 |
|  |  |  |  |  |  |  |  |  | C VS D | Tukey | 0.885 | -0.032 | -0.383 | 0.330 |
| Post-stroke | 18 | 26 | 22 | 25 |  |  |  |  | A VS B | Tukey | 0.644 | 0.166 | -0.265 | 0.530 |
|  |  |  |  |  |  |  |  |  | A VS C | Tukey | 0.454 | 0.237 | -0.235 | 0.612 |
|  |  |  |  |  |  |  |  |  | B VS D | Tukey | 0.008 | 0.410 | 0.084 | 0.825 |
|  |  |  |  |  |  |  |  |  | C VS D | Tukey | 0.042 | 0.448 | 0.011 | 0.776 |
| Fig. 4E RF stride distance |  |  |  |  | Yes | two-way ANOVA | *F* (3, 242) = 1.433 | 0.206 | Interaction |  |  |  |  |  |
| Fig. 4E RF stride distance |  |  |  |  |  |  | *F* (1, 242) = 21.32 | <0.001 | Time effect |  |  |  |  |  |
| Fig. 4E RF stride distance |  |  |  |  |  |  | *F* (3, 242) = 7.458 | <0.001 | Main effect |  |  |  |  |  |
| Before-stroke | 25 | 43 | 26 | 40 |  |  |  |  | A VS B | Tukey | 0.676 | 0.137 | -0.244 | 0.418 |
|  |  |  |  |  |  |  |  |  | A VS C | Tukey | 0.377 | 0.257 | -0.158 | 0.605 |
|  |  |  |  |  |  |  |  |  | B VS D | Tukey | 0.347 | 0.188 | -0.114 | 0.413 |
|  |  |  |  |  |  |  |  |  | C VS D | Tukey | 0.856 | 0.057 | -0.312 | 0.448 |
| Post-stroke | 18 | 26 | 22 | 25 |  |  |  |  | A VS B | Tukey | 0.584 | 0.202 | -0.264 | 0.568 |
|  |  |  |  |  |  |  |  |  | A VS C | Tukey | 0.145 | 0.404 | -0.084 | 0.804 |
|  |  |  |  |  |  |  |  |  | B VS D | Tukey | 0.004 | 0.462 | 0.134 | 1.008 |
|  |  |  |  |  |  |  |  |  | C VS D | Tukey | 0.157 | 0.357 | -0.083 | 0.730 |
| Fig. 4F RH stride distance |  |  |  |  | Yes | two-way ANOVA | *F* (3, 242) = 1.261 | 0.274 | Interaction |  |  |  |  |  |
| Fig. 4F RH stride distance |  |  |  |  |  |  | *F* (1, 242) = 13.00 | <0.001 | Time effect |  |  |  |  |  |
| Fig. 4F RH stride distance |  |  |  |  |  |  | *F* (3, 242) = 5.854 | <0.001 | Main effect |  |  |  |  |  |
| Before-stroke | 25 | 43 | 26 | 40 |  |  |  |  | A VS B | Tukey | 0.872 | 0.044 | -0.318 | 0.426 |
|  |  |  |  |  |  |  |  |  | A VS C | Tukey | 0.804 | 0.112 | -0.315 | 0.440 |
|  |  |  |  |  |  |  |  |  | B VS D | Tukey | 0.245 | 0.222 | -0.075 | 0.428 |
|  |  |  |  |  |  |  |  |  | C VS D | Tukey | 0.560 | 0.153 | -0.208 | 0.434 |
| Post-stroke | 18 | 26 | 22 | 25 |  |  |  |  | A VS B | Tukey | 0.764 | 0.135 | -0.330 | 0.503 |
|  |  |  |  |  |  |  |  |  | A VS C | Tukey | 0.745 | 0.140 | -0.337 | 0.537 |
|  |  |  |  |  |  |  |  |  | B VS D | Tukey | 0.010 | 0.418 | 0.081 | 0.846 |
|  |  |  |  |  |  |  |  |  | C VS D | Tukey | 0.022 | 0.404 | 0.044 | 0.845 |
| Fig. 4G LF contact time |  |  |  |  | Yes | two-way ANOVA | *F* (3, 51) = 3.484 | 0.021 | Interaction |  |  |  |  |  |
| Fig. 4G LF contact time |  |  |  |  |  |  | *F* (1, 51) = 22.44 | <0.001 | Time effect |  |  |  |  |  |
| Fig. 4G LF contact time |  |  |  |  |  |  | *F* (3, 76) = 3.536 | 0.015 | Main effect |  |  |  |  |  |
| Before-stroke | 14 | 27 | 18 | 25 |  |  |  |  | A VS B | Tukey | 0.464 | 4.265 | -5.763 | 16.430 |
|  |  |  |  |  |  |  |  |  | A VS C | Tukey | 0.760 | 3.081 | -10.030 | 15.220 |
|  |  |  |  |  |  |  |  |  | B VS D | Tukey | 0.582 | -4.372 | -14.640 | 4.874 |
|  |  |  |  |  |  |  |  |  | C VS D | Tukey | 0.850 | -2.186 | -13.580 | 8.287 |
| Post-stroke | 14 | 16 | 18 | 16 |  |  |  |  | A VS B | Tukey | >0.888 | -0.054 | -13.440 | 13.310 |
|  |  |  |  |  |  |  |  |  | A VS C | Tukey | 0.861 | 2.304 | -10.720 | 14.430 |
|  |  |  |  |  |  |  |  |  | B VS D | Tukey | 0.008 | -14.730 | -27.620 | -2.835 |
|  |  |  |  |  |  |  |  |  | C VS D | Tukey | 0.002 | -17.200 | -30.730 | -4.453 |
| Fig. 4H LF removal time |  |  |  |  | Yes | two-way ANOVA | *F* (3, 147) = 4.128 | 0.002 | Interaction |  |  |  |  |  |
| Fig. 4H LF removal time |  |  |  |  |  |  | *F* (1, 147) = 31.60 | <0.001 | Time effect |  |  |  |  |  |
| Fig. 4H LF removal time |  |  |  |  |  |  | *F* (3, 147) = 6.837 | <0.001 | Main effect |  |  |  |  |  |
| Before-stroke | 14 | 27 | 18 | 25 |  |  |  |  | A VS B | Tukey | 0.774 | 3.245 | -7.278 | 14.670 |
|  |  |  |  |  |  |  |  |  | A VS C | Tukey | 0.886 | 1.001 | -11.440 | 13.440 |
|  |  |  |  |  |  |  |  |  | B VS D | Tukey | 0.574 | -4.185 | -14.010 | 4.523 |
|  |  |  |  |  |  |  |  |  | C VS D | Tukey | 0.855 | -1.841 | -12.730 | 7.830 |
| Post-stroke | 14 | 16 | 18 | 16 |  |  |  |  | A VS B | Tukey | 0.782 | 3.488 | -8.262 | 15.260 |
|  |  |  |  |  |  |  |  |  | A VS C | Tukey | 0.187 | 8.417 | -2.834 | 21.860 |
|  |  |  |  |  |  |  |  |  | B VS D | Tukey | <0.001 | -18.670 | -32.140 | -6.415 |
|  |  |  |  |  |  |  |  |  | C VS D | Tukey | <0.001 | -24.700 | -36.740 | -13.650 |
| Fig. 4I RF contact time |  |  |  |  | Yes | two-way ANOVA | *F* (3, 51) = 0.371 | 0.656 | Interaction |  |  |  |  |  |
| Fig. 4I RF contact time |  |  |  |  |  |  | *F* (1, 51) = 0.427 | 0.414 | Time effect |  |  |  |  |  |
| Fig. 4I RF contact time |  |  |  |  |  |  | *F* (3, 76) = 0.082 | 0.854 | Main effect |  |  |  |  |  |
| Before-stroke | 14 | 27 | 18 | 25 |  |  |  |  |  |  |  |  |  |  |
| Post-stroke | 14 | 16 | 18 | 16 |  |  |  |  |  |  |  |  |  |  |
| Fig. 4J RF removal time |  |  |  |  | Yes | two-way ANOVA | *F* (3, 147) = 2.824 | 0.035 | Interaction |  |  |  |  |  |
| Fig. 4J RF removal time |  |  |  |  |  |  | *F* (1, 147) = 0.413 | 0.464 | Time effect |  |  |  |  |  |
| Fig. 4J RF removal time |  |  |  |  |  |  | *F* (3, 147) = 4.040 | 0.002 | Main effect |  |  |  |  |  |
| Before-stroke | 14 | 27 | 18 | 25 |  |  |  |  | A VS B | Tukey | 0.811 | 4.440 | -12.840 | 21.730 |
|  |  |  |  |  |  |  |  |  | A VS C | Tukey | 0.754 | 4.533 | -13.140 | 24.410 |
|  |  |  |  |  |  |  |  |  | B VS D | Tukey | 0.741 | -4.616 | -18.420 | 10.080 |
|  |  |  |  |  |  |  |  |  | C VS D | Tukey | 0.675 | -4.810 | -22.320 | 10.400 |
| Post-stroke | 14 | 16 | 18 | 16 |  |  |  |  | A VS B | Tukey | 0.512 | -8.065 | -27.330 | 10.170 |
|  |  |  |  |  |  |  |  |  | A VS C | Tukey | 0.750 | 4.581 | -13.070 | 24.460 |
|  |  |  |  |  |  |  |  |  | B VS D | Tukey | 0.144 | -14.140 | -33.700 | 3.484 |
|  |  |  |  |  |  |  |  |  | C VS D | Tukey | <0.001 | -28.820 | -47.060 | -11.660 |
| Fig. 5A Total iron content | 5 | 5 | 5 | 5 | Yes | two-way ANOVA | *F*(1, 20)=3.778 | 0.053 | Interaction |  |  |  |  |  |
| Fig. 5A Total iron content |  |  |  |  |  |  | *F*(1, 20)=10.517 | 0.004 | Main effect (gene) |  |  |  |  |  |
| Fig. 5A Total iron content |  |  |  |  |  |  | *F*(1, 20)=3.600 | 0.058 | Main effect  (treatment-dMCAO) |  |  |  |  |  |
|  |  |  |  |  |  |  |  |  | A VS B | Tukey | 0.044 | -2.741 | -4.627 | 0.045 |
|  |  |  |  |  |  |  |  |  | A VS C | Tukey | 0.700 | 0.837 | -1.848 | 3.724 |
|  |  |  |  |  |  |  |  |  | B VS D | Tukey | 0.006 | 3.714 | 0.827 | 5.602 |
|  |  |  |  |  |  |  |  |  | C VS D | Tukey | 1.000 | 0.034 | -2.742 | 2.822 |
| Fig. 5C FtL expression | 5 | 5 | 5 | 5 | Yes | two-way ANOVA | *F*(1, 20)=21.225 | <0.001 | Interaction |  |  |  |  |  |
| Fig. 5C FtL expression |  |  |  |  |  |  | *F*(1, 20)=17.057 | <0.001 | Main effect (gene) |  |  |  |  |  |
| Fig. 5C FtL expression |  |  |  |  |  |  | *F*(1, 20)=148.885 | <0.001 | Main effect  (treatment-dMCAO) |  |  |  |  |  |
|  |  |  |  |  |  |  |  |  | A VS B | Tukey | <0.001 | -0.770 | -1.075 | -0.563 |
|  |  |  |  |  |  |  |  |  | A VS C | Tukey | 0.884 | -0.018 | -0.224 | 0.177 |
|  |  |  |  |  |  |  |  |  | B VS D | Tukey | <0.001 | 0.452 | 0.245 | 0.588 |
|  |  |  |  |  |  |  |  |  | C VS D | Tukey | <0.001 | -0.388 | -0.504 | -0.182 |
| Fig. 5D FtH expression | 5 | 5 | 5 | 5 | Yes | two-way ANOVA | *F*(1, 20)=0.018 | 0.782 | Interaction |  |  |  |  |  |
| Fig. 5D FtH expression |  |  |  |  |  |  | *F*(1, 20)=38.474 | <0.001 | Main effect (gene) |  |  |  |  |  |
| Fig. 5D FtH expression |  |  |  |  |  |  | *F*(1, 20)=4.417 | 0.031 | Main effect  (treatment-dMCAO) |  |  |  |  |  |
|  |  |  |  |  |  |  |  |  | A VS B | Tukey | 0.328 | -0.111 | -0.280 | 0.056 |
|  |  |  |  |  |  |  |  |  | A VS C | Tukey | 0.002 | 0.267 | 0.088 | 0.445 |
|  |  |  |  |  |  |  |  |  | B VS D | Tukey | 0.001 | 0.280 | 0.112 | 0.458 |
|  |  |  |  |  |  |  |  |  | C VS D | Tukey | 0.428 | -0.088 | -0.266 | 0.070 |
| Fig. 5E TfR1 expression | 5 | 5 | 5 | 5 | Yes | two-way ANOVA | *F*(1, 20)=17.755 | <0.001 | Interaction |  |  |  |  |  |
| Fig. 5E TfR1 expression |  |  |  |  |  |  | *F*(1, 20)=64.813 | <0.001 | Main effect (gene) |  |  |  |  |  |
| Fig. 5E TfR1 expression |  |  |  |  |  |  | *F*(1, 20)=14.135 | 0.001 | Main effect  (treatment-dMCAO) |  |  |  |  |  |
|  |  |  |  |  |  |  |  |  | A VS B | Tukey | <0.001 | 0.408 | 0.208 | 0.508 |
|  |  |  |  |  |  |  |  |  | A VS C | Tukey | 0.026 | -0.221 | -0.420 | -0.021 |
|  |  |  |  |  |  |  |  |  | B VS D | Tukey | <0.001 | -0.548 | -0.748 | -0.448 |
|  |  |  |  |  |  |  |  |  | C VS D | Tukey | 0.865 | -0.028 | -0.228 | 0.160 |
| Fig. 5F FPN1 expression | 5 | 5 | 5 | 5 | Yes | two-way ANOVA | *F*(1, 20)=4.654 | 0.041 | Interaction |  |  |  |  |  |
| Fig. 5F FPN1 expression |  |  |  |  |  |  | *F*(1, 20)=4.078 | 0.046 | Main effect (gene) |  |  |  |  |  |
| Fig. 5F FPN1 expression |  |  |  |  |  |  | *F*(1, 20)=0.474 | 0.443 | Main effect  (treatment-dMCAO) |  |  |  |  |  |
|  |  |  |  |  |  |  |  |  | A VS B | Tukey | 0.182 | -0.284 | -0.580 | 0.101 |
|  |  |  |  |  |  |  |  |  | A VS C | Tukey | 1.000 | -0.015 | -0.412 | 0.368 |
|  |  |  |  |  |  |  |  |  | B VS D | Tukey | 0.034 | 0.420 | 0.024 | 0.715 |
|  |  |  |  |  |  |  |  |  | C VS D | Tukey | 0.640 | 0.142 | -0.244 | 0.436 |
| Fig. 6A Body Weight |  |  |  |  | Yes | two-way ANOVA | *F* (3, 65) = 0.2447 | 0.747 | Interaction |  |  |  |  |  |
| Fig. 6A Body Weight |  |  |  |  |  |  | *F* (1, 65) = 1.046 | 0.310 | Time effect |  |  |  |  |  |
| Fig. 6A Body Weight |  |  |  |  |  |  | *F* (3, 146) = 1.868 | 0.120 | Main effect |  |  |  |  |  |
| Before-stroke | 24 | 46 | 24 | 44 |  |  |  |  |  |  |  |  |  |  |
| Post-stroke | 18 | 15 | 24 | 21 |  |  |  |  |  |  |  |  |  |  |
| Fig. 6B Neurological score | 14 | 18 | 16 | 20 | Yes | two-way ANOVA | *F* (1, 55) = 7.140 | 0.005 | Interaction |  |  |  |  |  |
| Fig. 6B Neurological score |  |  |  |  |  |  | *F* (1, 55) = 4.680 | <0.001 | Main effect (gene) |  |  |  |  |  |
| Fig. 6B Neurological score |  |  |  |  |  |  | *F* (1, 55) = 50.83 | 0.018 | Main effect  (treatment-dMCAO) |  |  |  |  |  |
|  |  |  |  |  |  |  |  |  | A VS C | Tukey | 0.881 | 0.065 | -0.478 | 0.640 |
|  |  |  |  |  |  |  |  |  | A VS B | Tukey | 0.005 | -0.734 | -1.473 | -0.175 |
|  |  |  |  |  |  |  |  |  | C VS D | Tukey | <0.001 | -1.686 | -2.404 | -1.180 |
|  |  |  |  |  |  |  |  |  | B VS D | Tukey | 0.001 | -0.776 | -1.466 | -0.286 |
| Fig. 6C LF stride distance |  |  |  |  | Yes | two-way ANOVA | *F* (3, 231) = 2.202 | 0.078 | Interaction |  |  |  |  |  |
| Fig. 6C LF stride distance |  |  |  |  |  |  | *F* (1, 231) = 7.603 | 0.004 | Time effect |  |  |  |  |  |
| Fig. 6C LF stride distance |  |  |  |  |  |  | *F* (3, 231) = 4.467 | 0.001 | Main effect |  |  |  |  |  |
| Before-stroke | 25 | 43 | 26 | 40 |  |  |  |  | A VS B | Tukey | 0.404 | 0.222 | -0.145 | 0.480 |
|  |  |  |  |  |  |  |  |  | A VS C | Tukey | 0.257 | 0.285 | -0.125 | 0.618 |
|  |  |  |  |  |  |  |  |  | B VS D | Tukey | 0.603 | 0.125 | -0.166 | 0.428 |
|  |  |  |  |  |  |  |  |  | C VS D | Tukey | 0.873 | 0.042 | -0.314 | 0.418 |
| Post-stroke | 15 | 22 | 23 | 22 |  |  |  |  | A VS B | Tukey | 0.528 | -0.234 | -0.638 | 0.261 |
|  |  |  |  |  |  |  |  |  | A VS C | Tukey | 0.377 | 0.306 | -0.184 | 0.706 |
|  |  |  |  |  |  |  |  |  | B VS D | Tukey | 0.006 | 0.474 | 0.122 | 1.047 |
|  |  |  |  |  |  |  |  |  | C VS D | Tukey | 0.884 | 0.044 | -0.414 | 0.403 |
| Fig. 6D LH stride distance |  |  |  |  | Yes | two-way ANOVA | *F* (3, 231) = 1.218 | 0.304 | Interaction |  |  |  |  |  |
| Fig. 6D LH stride distance |  |  |  |  |  |  | *F* (1, 231) = 3.073 | 0.070 | Time effect |  |  |  |  |  |
| Fig. 6D LH stride distance |  |  |  |  |  |  | *F* (3, 231) = 7.605 | <0.001 | Main effect |  |  |  |  |  |
| Before-stroke | 25 | 43 | 26 | 40 |  |  |  |  | A VS B | Tukey | 0.884 | 0.034 | -0.321 | 0.382 |
|  |  |  |  |  |  |  |  |  | A VS C | Tukey | 0.175 | 0.317 | -0.081 | 0.626 |
|  |  |  |  |  |  |  |  |  | B VS D | Tukey | 0.110 | 0.246 | -0.036 | 0.440 |
|  |  |  |  |  |  |  |  |  | C VS D | Tukey | 0.886 | -0.026 | -0.372 | 0.328 |
| Post-stroke | 15 | 22 | 23 | 22 |  |  |  |  | A VS B | Tukey | 0.554 | -0.215 | -0.604 | 0.263 |
|  |  |  |  |  |  |  |  |  | A VS C | Tukey | 0.044 | 0.466 | -0.006 | 0.852 |
|  |  |  |  |  |  |  |  |  | B VS D | Tukey | 0.024 | 0.484 | 0.044 | 0.843 |
|  |  |  |  |  |  |  |  |  | C VS D | Tukey | 0.542 | -0.188 | -0.543 | 0.244 |
| Fig. 6E RF stride distance |  |  |  |  | Yes | two-way ANOVA | *F* (3, 231) = 2.002 | 0.114 | Interaction |  |  |  |  |  |
| Fig. 6E RF stride distance |  |  |  |  |  |  | *F* (1, 231) = 2.180 | 0.140 | Time effect |  |  |  |  |  |
| Fig. 6E RF stride distance |  |  |  |  |  |  | *F* (3, 231) = 5.377 | <0.001 | Main effect |  |  |  |  |  |
| Before-stroke | 25 | 43 | 26 | 40 |  |  |  |  | A VS B | Tukey | 0.777 | 0.100 | -0.247 | 0.447 |
|  |  |  |  |  |  |  |  |  | A VS C | Tukey | 0.211 | 0.308 | -0.102 | 0.620 |
|  |  |  |  |  |  |  |  |  | B VS D | Tukey | 0.153 | 0.236 | -0.047 | 0.432 |
|  |  |  |  |  |  |  |  |  | C VS D | Tukey | 0.886 | 0.027 | -0.328 | 0.374 |
| Post-stroke | 15 | 22 | 23 | 22 |  |  |  |  | A VS B | Tukey | 0.173 | -0.374 | -0.764 | 0.107 |
|  |  |  |  |  |  |  |  |  | A VS C | Tukey | 0.466 | 0.261 | -0.215 | 0.646 |
|  |  |  |  |  |  |  |  |  | B VS D | Tukey | 0.048 | 0.448 | -0.002 | 0.800 |
|  |  |  |  |  |  |  |  |  | C VS D | Tukey | 0.534 | -0.204 | -0.541 | 0.241 |
| Fig. 6F RH stride distance |  |  |  |  | Yes | two-way ANOVA | *F* (3, 64) = 1.737 | 0.147 | Interaction |  |  |  |  |  |
| Fig. 6F RH stride distance |  |  |  |  |  |  | *F* (1, 64) = 4.455 | 0.037 | Time effect |  |  |  |  |  |
| Fig. 6F RH stride distance |  |  |  |  |  |  | *F* (3, 145) = 3.264 | 0.023 | Main effect |  |  |  |  |  |
| Before-stroke | 25 | 43 | 26 | 40 |  |  |  |  | A VS B | Tukey | 0.677 | 0.130 | -0.231 | 0.481 |
|  |  |  |  |  |  |  |  |  | A VS C | Tukey | 0.672 | 0.141 | -0.254 | 0.454 |
|  |  |  |  |  |  |  |  |  | B VS D | Tukey | 0.371 | 0.174 | -0.114 | 0.471 |
|  |  |  |  |  |  |  |  |  | C VS D | Tukey | 0.545 | 0.153 | -0.186 | 0.423 |
| Post-stroke | 15 | 22 | 23 | 22 |  |  |  |  | A VS B | Tukey | 0.404 | -0.256 | -0.652 | 0.228 |
|  |  |  |  |  |  |  |  |  | A VS C | Tukey | 0.514 | 0.232 | -0.248 | 0.622 |
|  |  |  |  |  |  |  |  |  | B VS D | Tukey | 0.131 | 0.374 | -0.061 | 0.737 |
|  |  |  |  |  |  |  |  |  | C VS D | Tukey | 0.812 | -0.114 | -0.454 | 0.334 |
| Fig. 6G LF contact time |  |  |  |  | Yes | two-way ANOVA | *F* (3, 140) = 3.665 | 0.012 | Interaction |  |  |  |  |  |
| Fig. 6G LF contact time |  |  |  |  |  |  | *F* (1, 140) = 12.58 | 0.001 | Time effect |  |  |  |  |  |
| Fig. 6G LF contact time |  |  |  |  |  |  | *F* (3, 140) = 4.201 | 0.002 | Main effect |  |  |  |  |  |
| Before-stroke | 12 | 27 | 18 | 25 |  |  |  |  | A VS B | Tukey | 0.722 | 4.087 | -7.180 | 15.380 |
|  |  |  |  |  |  |  |  |  | A VS C | Tukey | 0.868 | 2.008 | -11.120 | 14.140 |
|  |  |  |  |  |  |  |  |  | B VS D | Tukey | 0.550 | -4.275 | -13.870 | 4.414 |
|  |  |  |  |  |  |  |  |  | C VS D | Tukey | 0.841 | -2.186 | -12.840 | 7.442 |
| Post-stroke | 12 | 21 | 18 | 21 |  |  |  |  | A VS B | Tukey | 0.332 | -7.376 | -21.260 | 4.400 |
|  |  |  |  |  |  |  |  |  | A VS C | Tukey | 0.814 | -3.284 | -15.430 | 8.735 |
|  |  |  |  |  |  |  |  |  | B VS D | Tukey | 0.020 | -12.400 | -23.380 | -1.407 |
|  |  |  |  |  |  |  |  |  | C VS D | Tukey | 0.001 | -16.480 | -27.660 | -5.214 |
| Fig. 6H LF removal time |  |  |  |  | Yes | two-way ANOVA | *F* (3, 140) = 23.21 | <0.001 | Interaction |  |  |  |  |  |
| Fig. 6H LF removal time |  |  |  |  |  |  | *F* (1, 140) = 22.64 | <0.001 | Time effect |  |  |  |  |  |
| Fig. 6H LF removal time |  |  |  |  |  |  | *F* (3, 140) = 26.43 | <0.001 | Main effect |  |  |  |  |  |
| Before-stroke | 12 | 27 | 18 | 25 |  |  |  |  | A VS B | Tukey | 0.877 | 2.237 | -14.400 | 18.770 |
|  |  |  |  |  |  |  |  |  | A VS C | Tukey | >0.888 | -0.006 | -17.750 | 17.740 |
|  |  |  |  |  |  |  |  |  | B VS D | Tukey | 0.752 | -4.185 | -17.120 | 8.628 |
|  |  |  |  |  |  |  |  |  | C VS D | Tukey | 0.877 | -1.841 | -16.370 | 13.470 |
| Post-stroke | 12 | 21 | 18 | 21 |  |  |  |  | A VS B | Tukey | 0.146 | -14.200 | -33.600 | 3.302 |
|  |  |  |  |  |  |  |  |  | A VS C | Tukey | 0.804 | -4.846 | -23.700 | 13.810 |
|  |  |  |  |  |  |  |  |  | B VS D | Tukey | <0.001 | -41.820 | -56.600 | -35.140 |
|  |  |  |  |  |  |  |  |  | C VS D | Tukey | <0.001 | -52.170 | -67.360 | -44.880 |
| Fig. 6I RF contact time |  |  |  |  | Yes | two-way ANOVA | *F* (3, 140) = 1.750 | 0.138 | Interaction |  |  |  |  |  |
| Fig. 6I RF contact time |  |  |  |  |  |  | *F* (1, 140) = 0.328 | 0.456 | Time effect |  |  |  |  |  |
| Fig. 6I RF contact time |  |  |  |  |  |  | *F* (3, 140) = 2.284 | 0.070 | Main effect |  |  |  |  |  |
| Before-stroke | 12 | 27 | 18 | 25 |  |  |  |  |  |  |  |  |  |  |
| Post-stroke | 12 | 21 | 18 | 21 |  |  |  |  |  |  |  |  |  |  |
| Fig. 6J RF removal time |  |  |  |  | Yes | two-way ANOVA | *F* (3, 140) = 4.424 | 0.004 | Interaction |  |  |  |  |  |
| Fig. 6J RF removal time |  |  |  |  |  |  | *F* (1, 140) = 1.537 | 0.203 | Time effect |  |  |  |  |  |
| Fig. 6J RF removal time |  |  |  |  |  |  | *F* (3, 140) = 7.671 | <0.001 | Main effect |  |  |  |  |  |
| Before-stroke | 12 | 27 | 18 | 25 |  |  |  |  | A VS B | Tukey | 0.824 | 4.132 | -13.040 | 21.310 |
|  |  |  |  |  |  |  |  |  | A VS C | Tukey | 0.872 | 2.532 | -14.620 | 20.880 |
|  |  |  |  |  |  |  |  |  | B VS D | Tukey | 0.444 | -5.747 | -20.410 | 5.600 |
|  |  |  |  |  |  |  |  |  | C VS D | Tukey | 0.681 | -4.347 | -20.370 | 8.555 |
| Post-stroke | 12 | 21 | 18 | 21 |  |  |  |  | A VS B | Tukey | 0.802 | -4.654 | -22.670 | 13.240 |
|  |  |  |  |  |  |  |  |  | A VS C | Tukey | 0.557 | -7.024 | -25.370 | 10.330 |
|  |  |  |  |  |  |  |  |  | B VS D | Tukey | <0.001 | -27.670 | -44.140 | -13.420 |
|  |  |  |  |  |  |  |  |  | C VS D | Tukey | <0.001 | -24.420 | -41.270 | -8.652 |
| Fig. 7A Total iron content | 5 | 5 | 5 | 5 | Yes | two-way ANOVA | *F*(1, 20)=0.453 | 0.452 | Interaction |  |  |  |  |  |
| Fig. 7A Total iron content | 5 | 5 | 5 | 5 |  |  | *F*(1, 20)=5.050 | 0.023 | Main effect (gene) |  |  |  |  |  |
| Fig. 7A Total iron content | 5 | 5 | 5 | 5 |  |  | *F*(1, 20)=48.751 | <0.001 | Main effect  (treatment-dMCAO) |  |  |  |  |  |
|  |  |  |  |  |  |  |  |  | A VS B | Tukey | 0.001 | -2.271 | -3.612 | -0.740 |
|  |  |  |  |  |  |  |  |  | A VS C | Tukey | 0.138 | 1.151 | -0.260 | 2.482 |
|  |  |  |  |  |  |  |  |  | B VS D | Tukey | 0.527 | 0.518 | -0.712 | 2.048 |
|  |  |  |  |  |  |  |  |  | C VS D | Tukey | <0.001 | -2.724 | -4.244 | -1.383 |
| Fig. 7C FtL expression | 5 | 5 | 5 | 5 | Yes | two-way ANOVA | *F*(1, 20)=2.816 | 0.103 | Interaction |  |  |  |  |  |
| Fig. 7C FtL expression | 5 | 5 | 5 | 5 |  |  | *F*(1, 20)=8.623 | 0.004 | Main effect (gene) |  |  |  |  |  |
| Fig. 7C FtL expression | 5 | 5 | 5 | 5 |  |  | *F*(1, 20)=41.285 | <0.001 | Main effect  (treatment-dMCAO) |  |  |  |  |  |
|  |  |  |  |  |  |  |  |  | A VS B | Tukey | <0.001 | -0.548 | -0.868 | -0.337 |
|  |  |  |  |  |  |  |  |  | A VS C | Tukey | 0.643 | 0.114 | -0.205 | 0.434 |
|  |  |  |  |  |  |  |  |  | B VS D | Tukey | 0.014 | 0.381 | 0.060 | 0.611 |
|  |  |  |  |  |  |  |  |  | C VS D | Tukey | 0.015 | -0.372 | -0.603 | -0.052 |
| Fig. 7D FtH expression | 5 | 5 | 5 | 5 | Yes | two-way ANOVA | *F*(1, 20)=0.564 | 0.421 | Interaction |  |  |  |  |  |
| Fig. 7D FtH expression | 5 | 5 | 5 | 5 |  |  | *F*(1, 20)=30.414 | <0.001 | Main effect (gene) |  |  |  |  |  |
| Fig. 7D FtH expression | 5 | 5 | 5 | 5 |  |  | *F*(1, 20)=10.470 | 0.004 | Main effect  (treatment-dMCAO) |  |  |  |  |  |
|  |  |  |  |  |  |  |  |  | A VS B | Tukey | 0.345 | -0.213 | -0.453 | 0.135 |
|  |  |  |  |  |  |  |  |  | A VS C | Tukey | 0.001 | 0.448 | 0.210 | 0.808 |
|  |  |  |  |  |  |  |  |  | B VS D | Tukey | 0.016 | 0.414 | 0.054 | 0.654 |
|  |  |  |  |  |  |  |  |  | C VS D | Tukey | 0.043 | -0.347 | -0.607 | -0.008 |
| Fig. 7E TfR1 expression | 5 | 5 | 5 | 5 | Yes | two-way ANOVA | *F*(1, 20)=1.014 | 0.325 | Interaction |  |  |  |  |  |
| Fig. 7E TfR1 expression | 5 | 5 | 5 | 5 |  |  | *F*(1, 20)=31.658 | <0.001 | Main effect (gene) |  |  |  |  |  |
| Fig. 7E TfR1 expression | 5 | 5 | 5 | 5 |  |  | *F*(1, 20)=3.450 | 0.064 | Main effect  (treatment-dMCAO) |  |  |  |  |  |
|  |  |  |  |  |  |  |  |  | A VS B | Tukey | 0.204 | 0.156 | -0.051 | 0.384 |
|  |  |  |  |  |  |  |  |  | A VS C | Tukey | 0.017 | -0.255 | -0.484 | -0.038 |
|  |  |  |  |  |  |  |  |  | B VS D | Tukey | <0.001 | -0.372 | -0.510 | -0.144 |
|  |  |  |  |  |  |  |  |  | C VS D | Tukey | 0.824 | 0.041 | -0.166 | 0.267 |
| Fig. 7F FPN1 expression | 5 | 5 | 5 | 5 | Yes | two-way ANOVA | *F*(1, 20)=1.647 | 0.201 | Interaction |  |  |  |  |  |
| Fig. 7F FPN1 expression | 5 | 5 | 5 | 5 |  |  | *F*(1, 20)=14.412 | <0.001 | Main effect (gene) |  |  |  |  |  |
| Fig. 7F FPN1 expression | 5 | 5 | 5 | 5 |  |  | *F*(1, 20)=4.164 | 0.034 | Main effect  (treatment-dMCAO) |  |  |  |  |  |
|  |  |  |  |  |  |  |  |  | A VS B | Tukey | 0.805 | 0.028 | -0.081 | 0.147 |
|  |  |  |  |  |  |  |  |  | A VS C | Tukey | 0.274 | 0.067 | -0.041 | 0.187 |
|  |  |  |  |  |  |  |  |  | B VS D | Tukey | 0.006 | 0.147 | 0.038 | 0.266 |
|  |  |  |  |  |  |  |  |  | C VS D | Tukey | 0.073 | 0.107 | -0.011 | 0.226 |
| Fig. 7G GFAP expression | 5 | 5 | 5 | 5 | Yes | two-way ANOVA | *F*(1, 20)=16.031 | 0.001 | Interaction |  |  |  |  |  |
| Fig. 7G GFAP expression | 5 | 5 | 5 | 5 |  |  | *F*(1, 20)=0.243 | 0.520 | Main effect (gene) |  |  |  |  |  |
| Fig. 7G GFAP expression | 5 | 5 | 5 | 5 |  |  | *F*(1, 20)=106.704 | <0.001 | Main effect  (treatment-dMCAO) |  |  |  |  |  |
|  |  |  |  |  |  |  |  |  | A VS B | Tukey | 0.001 | -0.346 | -0.473 | -0.131 |
|  |  |  |  |  |  |  |  |  | A VS C | Tukey | 0.070 | 0.206 | -0.018 | 0.433 |
|  |  |  |  |  |  |  |  |  | B VS D | Tukey | 0.017 | -0.254 | -0.481 | -0.037 |
|  |  |  |  |  |  |  |  |  | C VS D | Tukey | <0.001 | -0.728 | -1.044 | -0.503 |
| Fig. 7J Quantification of immunofluorescence staining of FtL |  | 4 |  | 4 | Yes | Unpaired *t* test | *t*(5)*=*4.721 | 0.003 |  |  |  | -40.400 | -51.050 | -18.840 |
| Fig. 7K Quantification of immunofluorescence staining of FtH |  | 4 |  | 4 | Yes | Unpaired *t* test | *t*(5)*=*4.810 | 0.001 |  |  |  | -42.000 | -48.380 | -24.510 |
| Fig. 8E Quantification of immunofluorescence staining of GFAP |  | 4 |  | 4 | Yes | Unpaired *t* test | *t*(5)*=*-4.444 | 0.001 |  |  |  | 54.240 | 35.410 | 83.880 |
| Fig. 8F Quantification of immunofluorescence staining of Ki67 |  | 4 |  | 4 | Yes | Unpaired *t* test | *t*(5)*=*5.163 | 0.001 |  |  |  | -37.240 | -43.410 | -23.080 |
| Fig. 8G Quantification of immunofluorescence staining of DCX |  | 4 |  | 4 | Yes | Unpaired *t* test | *t*(5)*=*0.724 | 0.441 |  |  |  | -4.240 | -20.730 | 10.330 |
| Fig. 8H Quantification of immunofluorescence staining of NeuN |  | 4 |  | 4 | Yes | Unpaired *t* test | *t*(5)*=*10.406 | <0.001 |  |  |  | -100.000 | -123.400 | -65.480 |
| **Note**: **Ⅰ**. *Fpn1*^flox/flox^-Sham, **Ⅱ**. *Fpn1*^flox/flox^-dMCAO, **Ⅲ**. *Fpn1*^cdh4^-CKO-Sham, **Ⅳ**. *Fpn1*^cdh4^-CKO-dMCAO. * Shapiro-Wilk test of normality. | | | | | | | | | |  |  |  |  |  |
