# Supplementary material for: Cdh5-mediated Fpn1 deletion exerts neuroprotective effects during the acute phase and inhibitory effects during the recovery phase of ischemic stroke
Source: Cell Death Dis. 2023 Feb 25;14(2):161. doi: 10.1038/s41419-023-05688-1 (PMC9968354; doi:10.1038/s41419-023-05688-1)

Figure 2
Cerebral cortex

From left to right: *Fpn1*$^{\text{flox/flox}}$ Con 1, *Fpn1*$^{\text{flox/flox}}$ Con 2, *Fpn1*$^{\text{flox/flox}}$ Con 3, *Fpn1*$^{\text{flox/flox}}$ Ips 1, *Fpn1*$^{\text{flox/flox}}$ Ips 2, *Fpn1*$^{\text{flox/flox}}$ Ips 3, *Fpn1*$^{\text{cdh5}}$-CKO Con 1, *Fpn1*$^{\text{cdh5}}$-CKO Con 2, *Fpn1*$^{\text{cdh5}}$-CKO Con3, *Fpn1*$^{\text{cdh5}}$-CKO Ips 1, *Fpn1*$^{\text{cdh5}}$-CKO Ips 2, *Fpn1*$^{\text{cdh5}}$-CKO Ips 3

Membrane 1, Slice 1, probed with antibodies to **TfR1**

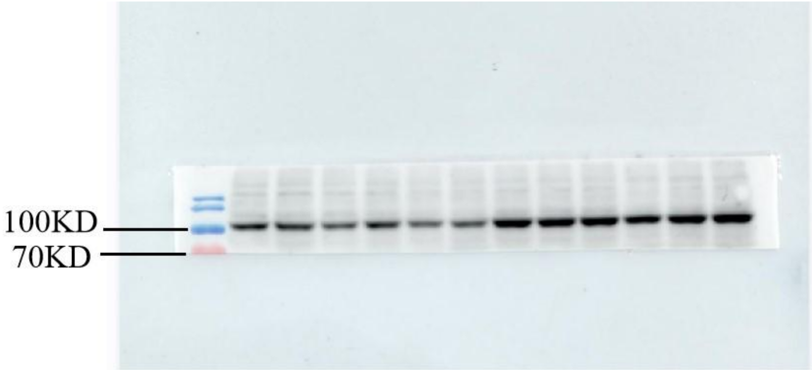

Membrane 1, Slice 2, probed with antibodies to **β-actin**

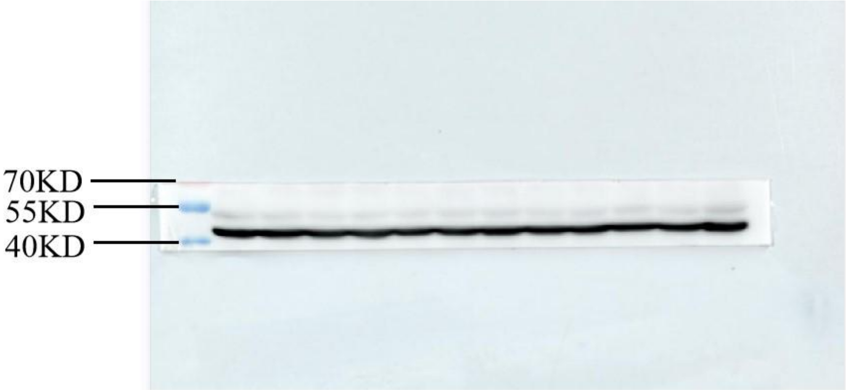

Membrane 1, Slice 3, probed with antibodies to **GAPDH**

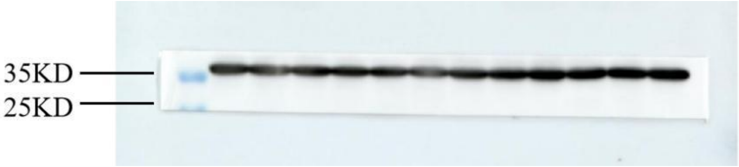

Membrane 1, Slice 4, probed with antibodies to **FtL**

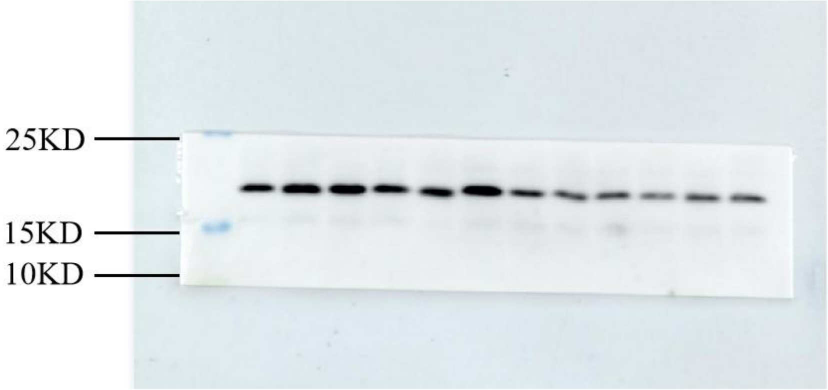

From left to right: *Fpn1*$^{flox/flox}$ Con 4, *Fpn1*$^{flox/flox}$ Con 5, *Fpn1*$^{flox/flox}$ Con 6, *Fpn1*$^{flox/flox}$ Ips 4, *Fpn1*$^{flox/flox}$ Ips 5, *Fpn1*$^{flox/flox}$ Ips 6, *Fpn1*^{cdh5}-CKO Con 4, *Fpn1*^{cdh5}-CKO Con 5, *Fpn1*^{cdh5}-CKO Con 6, *Fpn1*^{cdh5}-CKO Ips 4, *Fpn1*^{cdh5}-CKO Ips 5, *Fpn1*^{cdh5}-CKO Ips 6

Membrane 2, Slice 1, probed with antibodies to **TfR1**

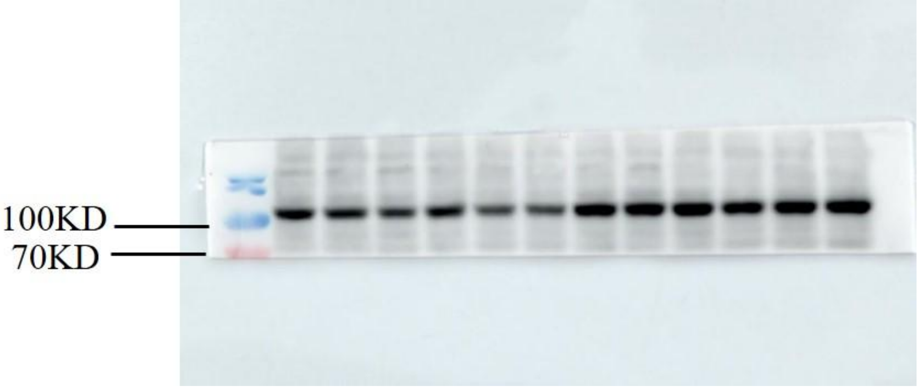

Membrane 2, Slice 2, probed with antibodies to **β-actin**

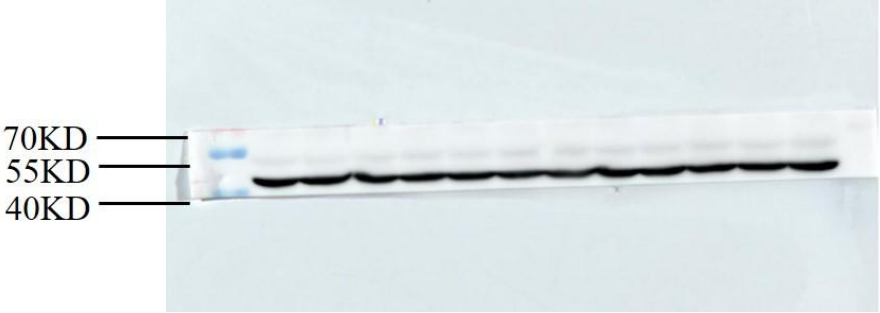

Membrane 2, Slice 3, probed with antibodies to **FtL**

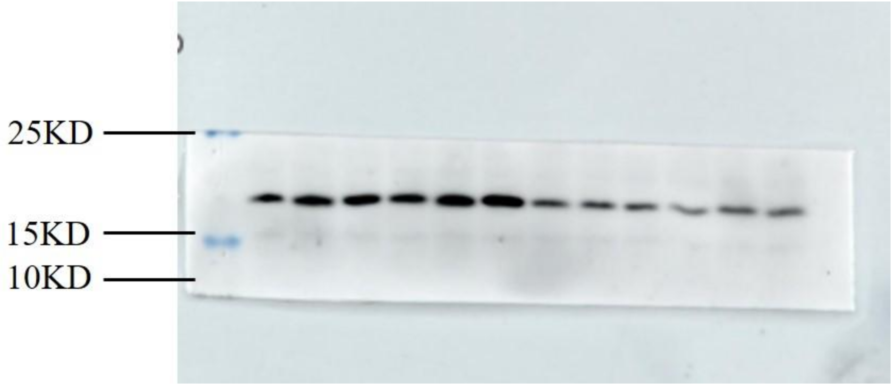

From left to right: *Fpn1*$^{flox/flox}$ Con 1, *Fpn1*$^{flox/flox}$ Con 2, *Fpn1*$^{flox/flox}$ Con 3, *Fpn1*$^{flox/flox}$ Ips 1, *Fpn1*$^{flox/flox}$ Ips 2, *Fpn1*$^{flox/flox}$ Ips 3, *Fpn1*^{cdh5}-CKO Con 1, *Fpn1*^{cdh5}-CKO Con 2, *Fpn1*^{cdh5}-CKO Con3, *Fpn1*^{cdh5}-CKO Ips 1, *Fpn1*^{cdh5}-CKO Ips 2, *Fpn1*^{cdh5}-CKO Ips 3

Membrane 3, Slice 1, probed with antibodies to **GAPDH**

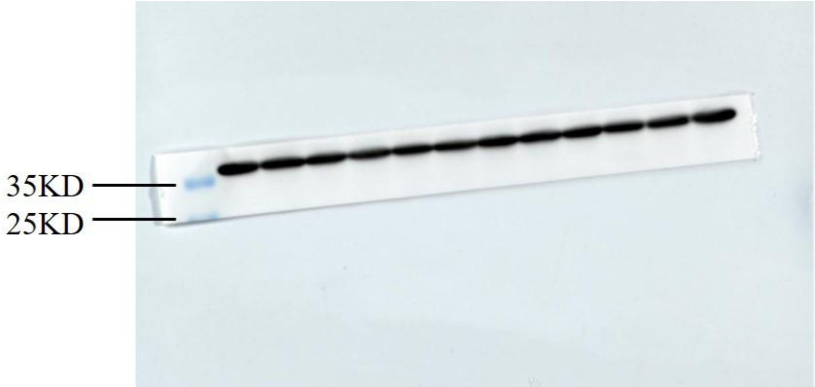

Membrane 3, Slice 2, probed with antibodies to **FtH**

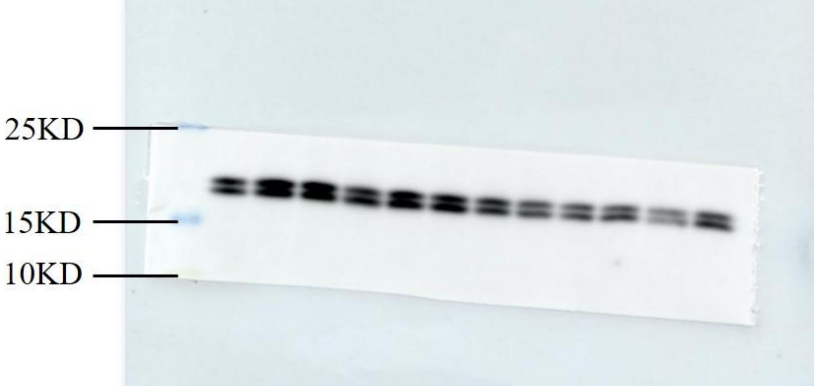

From left to right: *Fpn1*$^{flox/flox}$ Con 4, *Fpn1*$^{flox/flox}$ Con 5, *Fpn1*$^{flox/flox}$ Con 6, *Fpn1*$^{flox/flox}$ Ips 4, *Fpn1*$^{flox/flox}$ Ips 5, *Fpn1*$^{flox/flox}$ Ips 6, *Fpn1*^{cdh5}-CKO Con 4, *Fpn1*^{cdh5}-CKO Con 5, *Fpn1*^{cdh5}-CKO Con 6, *Fpn1*^{cdh5}-CKO Ips 4, *Fpn1*^{cdh5}-CKO Ips 5, *Fpn1*^{cdh5}-CKO Ips 6

Membrane 4, Slice 1, probed with antibodies to **GAPDH**

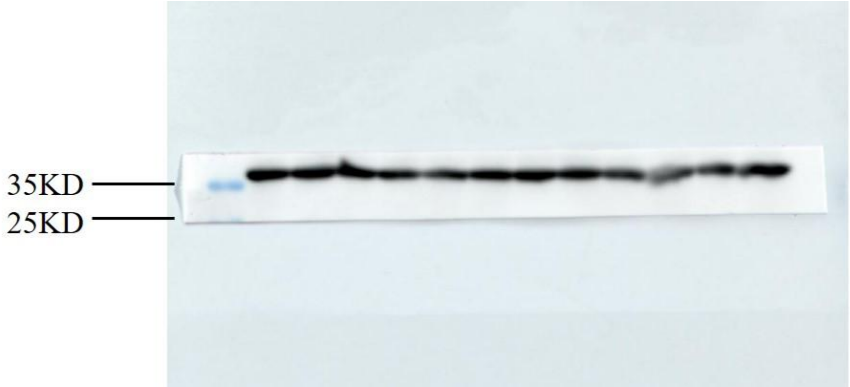

Membrane 4, Slice 2, probed with antibodies to **FtH**

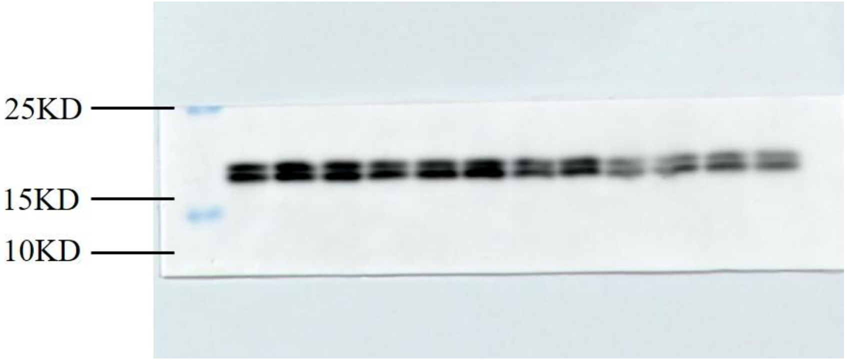

From left to right: *Fpn1*$^{flox/flox}$ Con 1, *Fpn1*$^{flox/flox}$ Con 2, *Fpn1*$^{flox/flox}$ Con 3, *Fpn1*$^{flox/flox}$ Ips 1, *Fpn1*$^{flox/flox}$ Ips 2, *Fpn1*$^{flox/flox}$ Ips 3, *Fpn1*^{cdh5}-CKO Con 1, *Fpn1*^{cdh5}-CKO Con 2, *Fpn1*^{cdh5}-CKO Con3, *Fpn1*^{cdh5}-CKO Ips 1, *Fpn1*^{cdh5}-CKO Ips 2, *Fpn1*^{cdh5}-CKO Ips 3

Membrane 5, Slice 1, probed with antibodies to **4HNE**

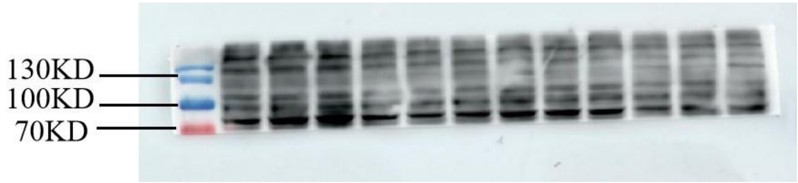

Membrane 5, Slice 2, probed with antibodies to **FPN1**

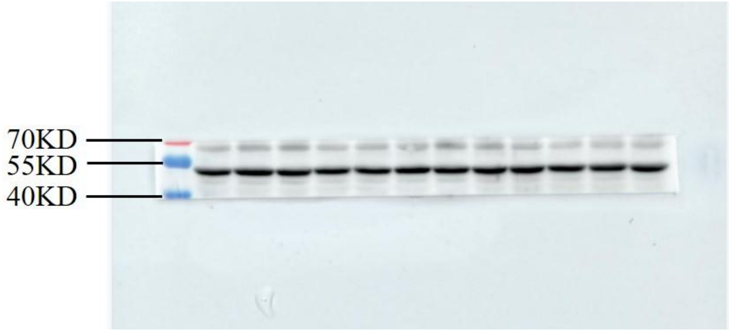

Membrane 5, Slice 2, probed with antibodies to **β-actin**

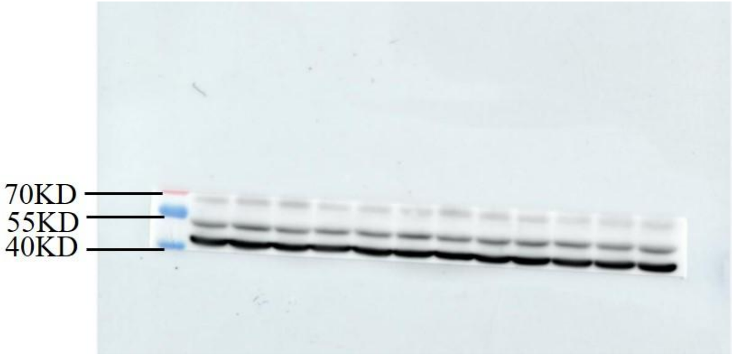

Membrane 5, Slice 3, probed with antibodies to **GAPDH**

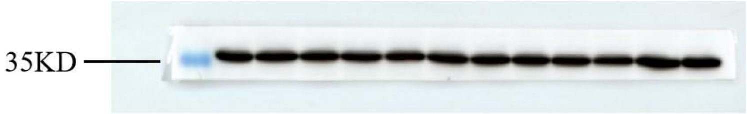

Membrane 5, Slice 4, probed with antibodies to **Bcl2**

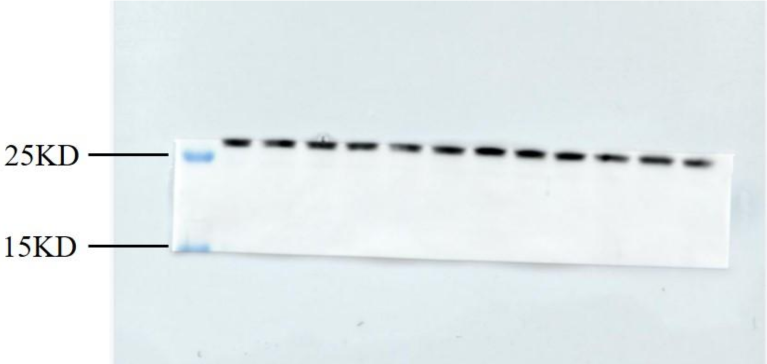

From left to right: *Fpn1*$^{flox/flox}$ Con 4, *Fpn1*$^{flox/flox}$ Con 5, *Fpn1*$^{flox/flox}$ Con 6, *Fpn1*$^{flox/flox}$ Ips 4, *Fpn1*$^{flox/flox}$ Ips 5, *Fpn1*$^{flox/flox}$ Ips 6, *Fpn1*^{cdh5}–CKO Con 4, *Fpn1*^{cdh5}–CKO Con 5, *Fpn1*^{cdh5}–CKO Con 6, *Fpn1*^{cdh5}–CKO Ips 4, *Fpn1*^{cdh5}–CKO Ips 5, *Fpn1*^{cdh5}–CKO Ips 6

Membrane 6, Slice 1, probed with antibodies to **FPN1**

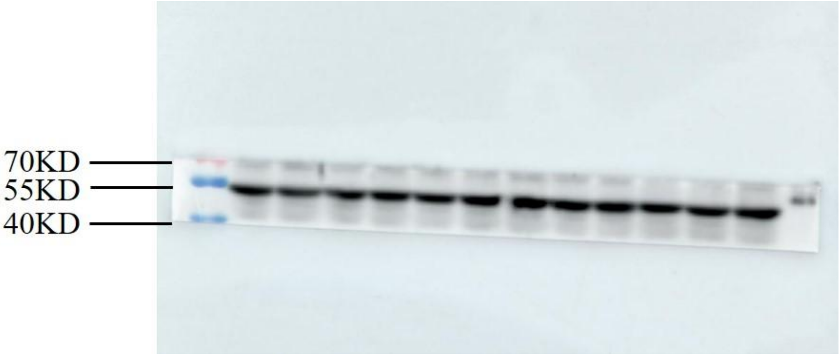

Membrane 6, Slice 2, probed with antibodies to **β-actin**

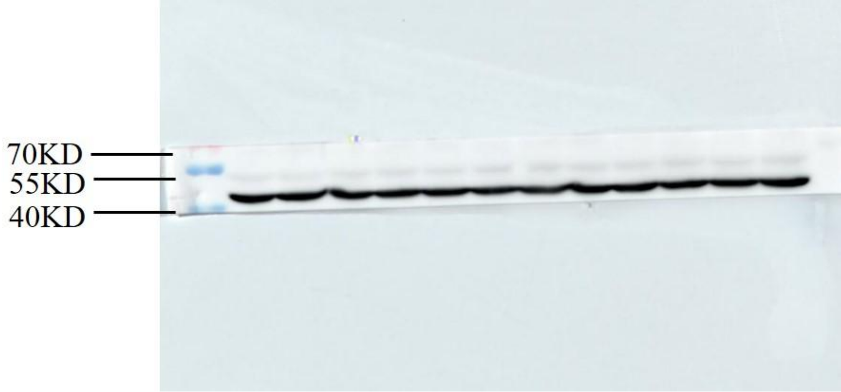

From left to right: *Fpn1*$^{flox/flox}$ Con 4, *Fpn1*$^{flox/flox}$ Con 5, *Fpn1*$^{flox/flox}$ Con 6, *Fpn1*$^{flox/flox}$ Ips 4, *Fpn1*$^{flox/flox}$ Ips 5, *Fpn1*$^{flox/flox}$ Ips 6, *Fpn1*^{cdh5}-CKO Con 4, *Fpn1*^{cdh5}-CKO Con 5, *Fpn1*^{cdh5}-CKO Con 6, *Fpn1*^{cdh5}-CKO Ips 4, *Fpn1*^{cdh5}-CKO Ips 5, *Fpn1*^{cdh5}-CKO Ips 6

Membrane 7, Slice 1, probed with antibodies to **GAPDH**

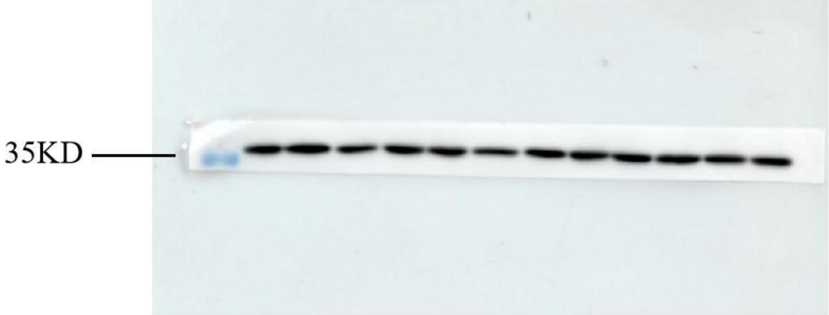

Membrane 7, Slice 2, probed with antibodies to **Bcl2**

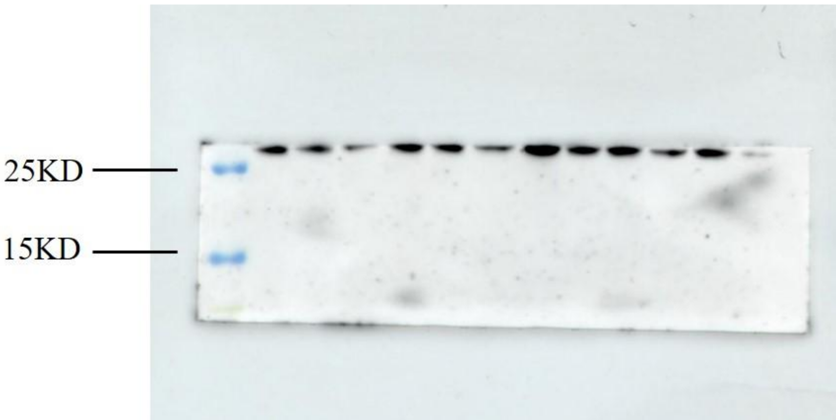

From left to right: *Fpn1*$^{flox/flox}$ Con 1, *Fpn1*$^{flox/flox}$ Con 2, *Fpn1*$^{flox/flox}$ Con 3, *Fpn1*$^{flox/flox}$ Ips 1, *Fpn1*$^{flox/flox}$ Ips 2, *Fpn1*$^{flox/flox}$ Ips 3, *Fpn1*^{cdh5}-CKO Con 1, *Fpn1*^{cdh5}-CKO Con 2, *Fpn1*^{cdh5}-CKO Con3, *Fpn1*^{cdh5}-CKO Ips 1, *Fpn1*^{cdh5}-CKO Ips 2, *Fpn1*^{cdh5}-CKO Ips 3

Membrane 8, Slice 1, probed with antibodies to **β-actin**

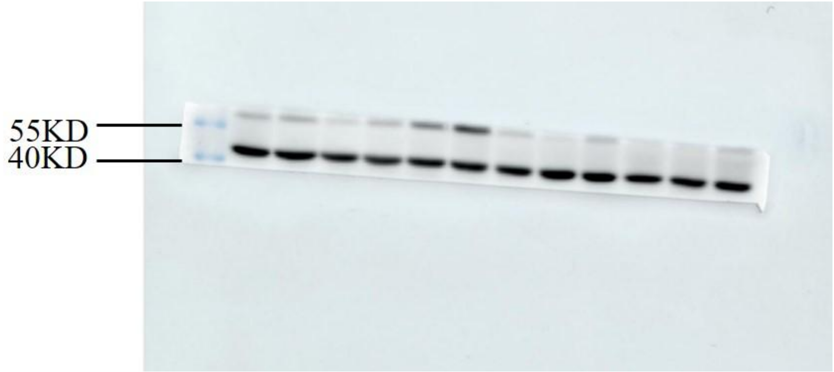

Membrane 8, Slice 2, probed with antibodies to **Bax**

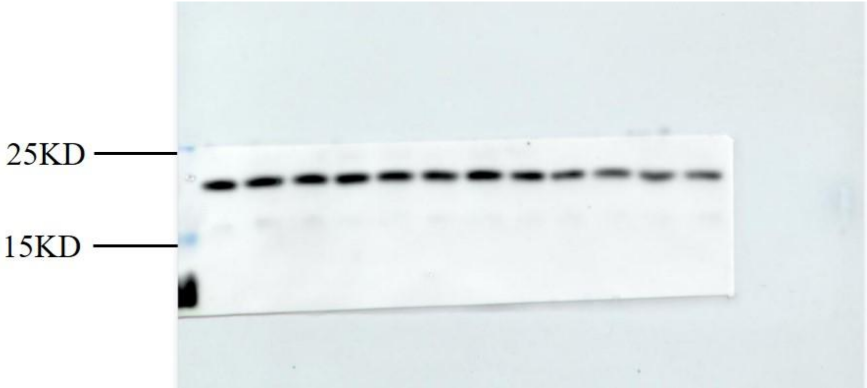

From left to right: *Fpn1*$^{flox/flox}$ Con 4, *Fpn1*$^{flox/flox}$ Con 5, *Fpn1*$^{flox/flox}$ Con 6, *Fpn1*$^{flox/flox}$ Ips 4, *Fpn1*$^{flox/flox}$ Ips 5, *Fpn1*$^{flox/flox}$ Ips 6, *Fpn1*^{cdh5}-CKO Con 4, *Fpn1*^{cdh5}-CKO Con 5, *Fpn1*^{cdh5}-CKO Con 6, *Fpn1*^{cdh5}-CKO Ips 4, *Fpn1*^{cdh5}-CKO Ips 5, *Fpn1*^{cdh5}-CKO Ips 6

Membrane 9, Slice 1, probed with antibodies to **β-actin**

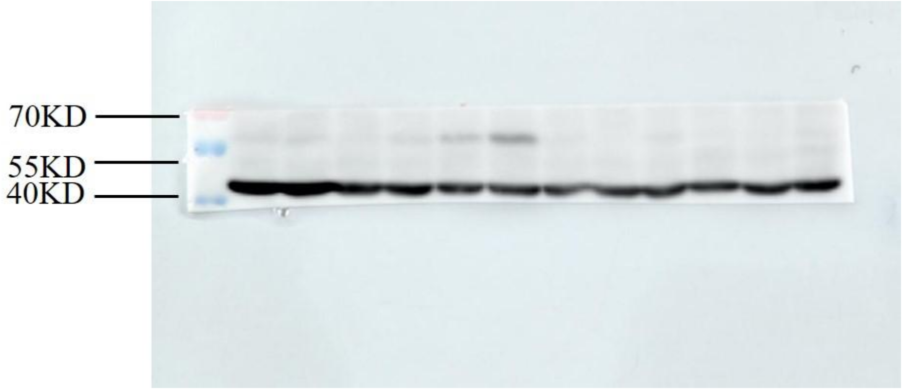

Membrane 9, Slice 2, probed with antibodies to **Bax**

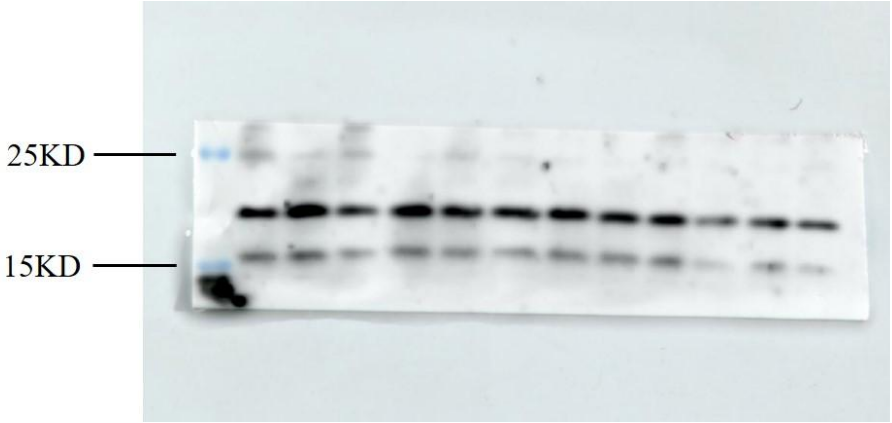

From left to right: *Fpn1*$^{flox/flox}$ Con 1, *Fpn1*$^{flox/flox}$ Con 2, *Fpn1*$^{flox/flox}$ Con 3, *Fpn1*$^{flox/flox}$ Ips 1, *Fpn1*$^{flox/flox}$ Ips 2, *Fpn1*$^{flox/flox}$ Ips 3, *Fpn1*^{cdh5}-CKO Con 1, *Fpn1*^{cdh5}-CKO Con 2, *Fpn1*^{cdh5}-CKO Con3, *Fpn1*^{cdh5}-CKO Ips 1, *Fpn1*^{cdh5}-CKO Ips 2, *Fpn1*^{cdh5}-CKO Ips 3

Membrane 10, Slice 1, probed with antibodies to **p-Erk 1/2**

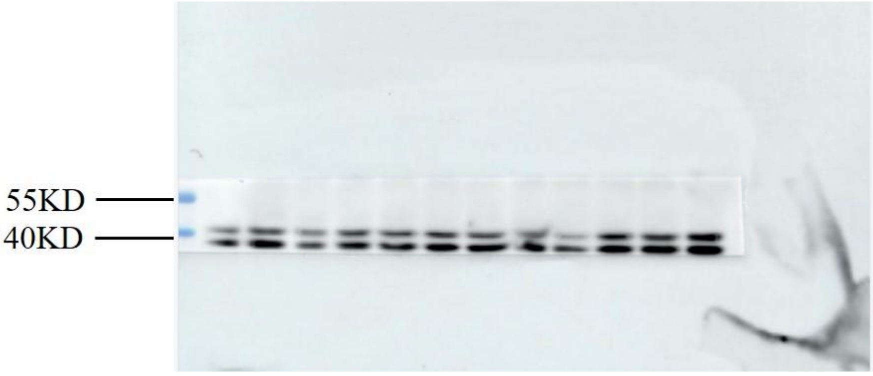

Membrane 10, Slice 1, probed with antibodies to **Erk 1/2**

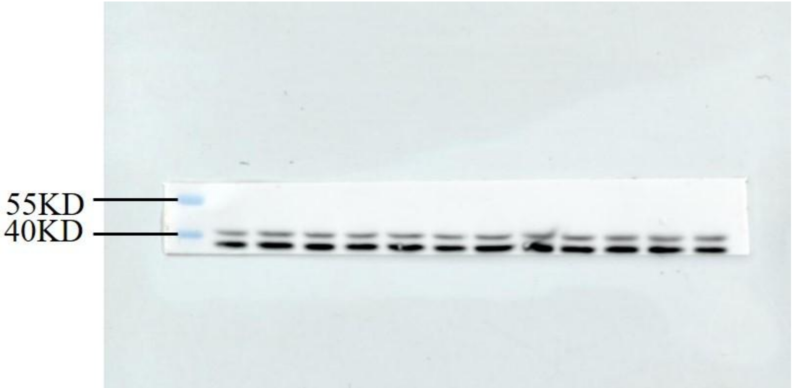

Membrane 10, Slice 2, probed with antibodies to **GAPDH**

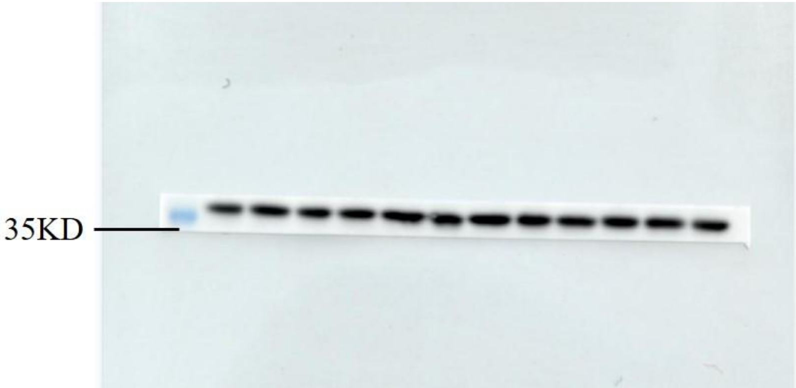

From left to right: *Fpn1*$^{flox/flox}$ Con 4, *Fpn1*$^{flox/flox}$ Con 5, *Fpn1*$^{flox/flox}$ Con 6, *Fpn1*$^{flox/flox}$ Ips 4, *Fpn1*$^{flox/flox}$ Ips 5, *Fpn1*$^{flox/flox}$ Ips 6, *Fpn1*^{cdh5}–CKO Con 4, *Fpn1*^{cdh5}–CKO Con 5, *Fpn1*^{cdh5}–CKO Con 6, *Fpn1*^{cdh5}–CKO Ips 4, *Fpn1*^{cdh5}–CKO Ips 5, *Fpn1*^{cdh5}–CKO Ips 6

Membrane 11, Slice 1, probed with antibodies to **p-Erk 1/2**

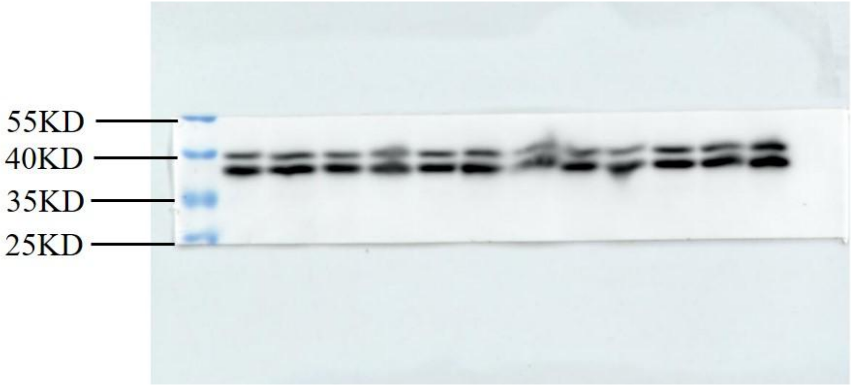

Membrane 11, Slice 1, probed with antibodies to **Erk 1/2**

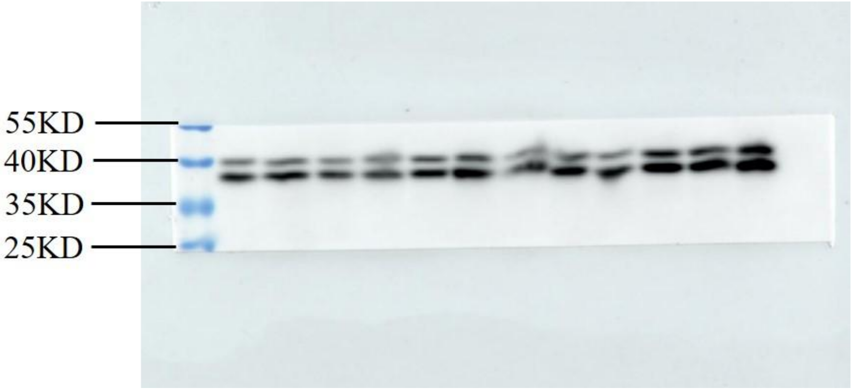

Membrane 11, Slice 1, probed with antibodies to **GAPDH**

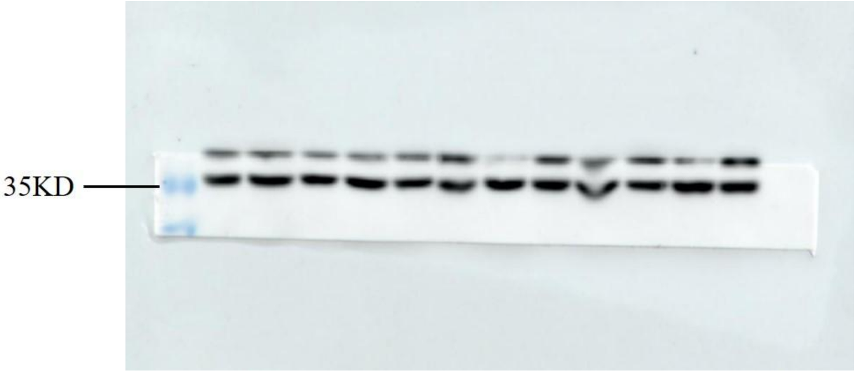

Supplement: Supplementary file 3 — Original Western Blots bands of Figure 2 [file 41419_2023_5688_MOESM3_ESM.pdf]
