# Supplementary material for: Cdh5-mediated Fpn1 deletion exerts neuroprotective effects during the acute phase and inhibitory effects during the recovery phase of ischemic stroke
Source: Cell Death Dis. 2023 Feb 25;14(2):161. doi: 10.1038/s41419-023-05688-1 (PMC9968354; doi:10.1038/s41419-023-05688-1)

Figure 3

Cerebral cortex

From left to right: *Fpn1*$^{flox/flox}$ Con 1, *Fpn1*$^{flox/flox}$ Con 2, *Fpn1*$^{flox/flox}$ Con 3, *Fpn1*$^{flox/flox}$ Ips 1, *Fpn1*$^{flox/flox}$ Ips 2, *Fpn1*$^{flox/flox}$ Ips 3, *Fpn1*^{cdh5}-CKO Con 1, *Fpn1*^{cdh5}-CKO Con 2, *Fpn1*^{cdh5}-CKO Con3, *Fpn1*^{cdh5}-CKO Ips 1, *Fpn1*^{cdh5}-CKO Ips 2, *Fpn1*^{cdh5}-CKO Ips 3

Membrane 12, Slice 1, probed with antibodies to **ACSL4**

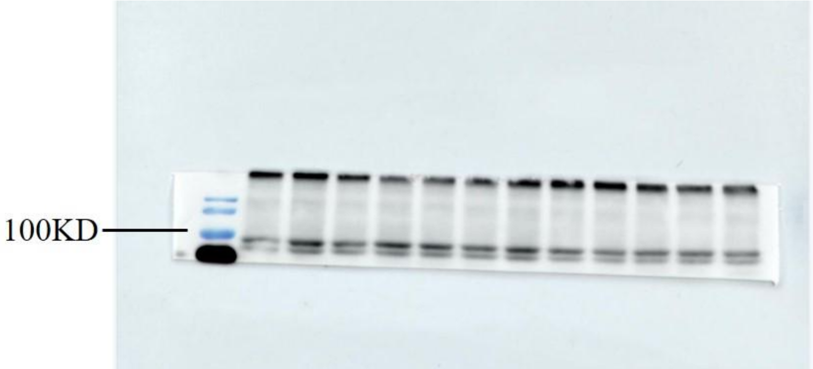

Membrane 12, Slice 2, probed with antibodies to **β-actin**

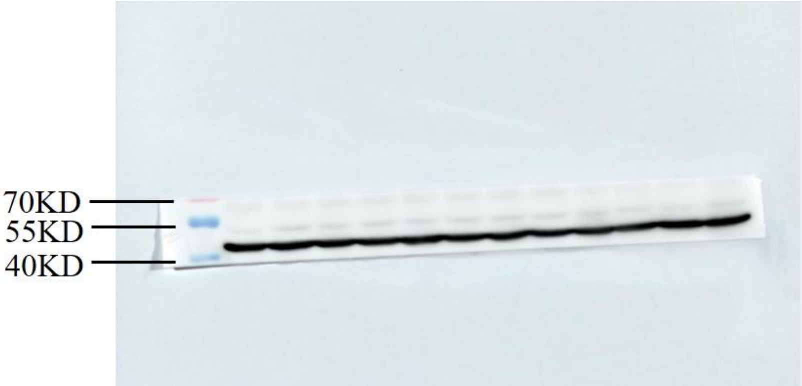

From left to right: *Fpn1*$^{\text{flox/flox}}$ Con 4, *Fpn1*$^{\text{flox/flox}}$ Con 5, *Fpn1*$^{\text{flox/flox}}$ Con 6, *Fpn1*$^{\text{flox/flox}}$ Ips 4, *Fpn1*$^{\text{flox/flox}}$ Ips 5, *Fpn1*$^{\text{flox/flox}}$ Ips 6, *Fpn1*$^{\text{cdh5}}$-CKO Con 4, *Fpn1*$^{\text{cdh5}}$-CKO Con 5, *Fpn1*$^{\text{cdh5}}$-CKO Con 6, *Fpn1*$^{\text{cdh5}}$-CKO Ips 4, *Fpn1*$^{\text{cdh5}}$-CKO Ips 5, *Fpn1*$^{\text{cdh5}}$-CKO Ips 6

Membrane 13, Slice 1, probed with antibodies to **ACSL4**

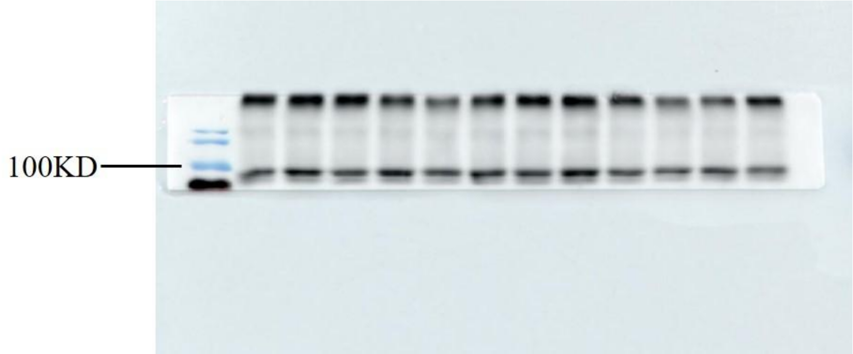

Membrane 13, Slice 2, probed with antibodies to **β-actin**

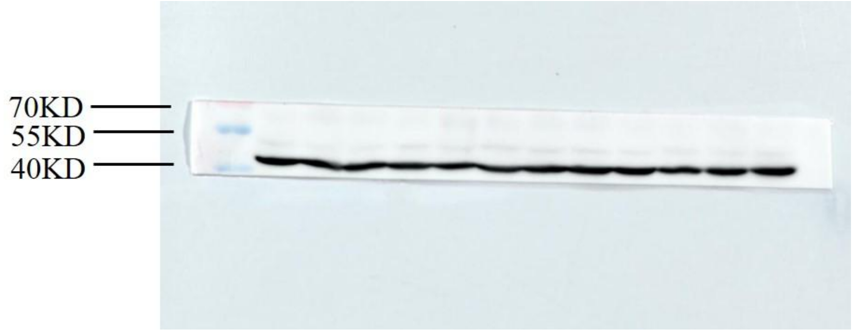

From left to right: *Fpn1*$^{flox/flox}$ Con 1, *Fpn1*$^{flox/flox}$ Con 2, *Fpn1*$^{flox/flox}$ Con 3, *Fpn1*$^{flox/flox}$ Ips 1, *Fpn1*$^{flox/flox}$ Ips 2, *Fpn1*$^{flox/flox}$ Ips 3, *Fpn1*^{cdh5}-CKO Con 1, *Fpn1*^{cdh5}-CKO Con 2, *Fpn1*^{cdh5}-CKO Con3, *Fpn1*^{cdh5}-CKO Ips 1, *Fpn1*^{cdh5}-CKO Ips 2, *Fpn1*^{cdh5}-CKO Ips 3

Membrane 14, Slice 1, probed with antibodies to **Total Nrf2**

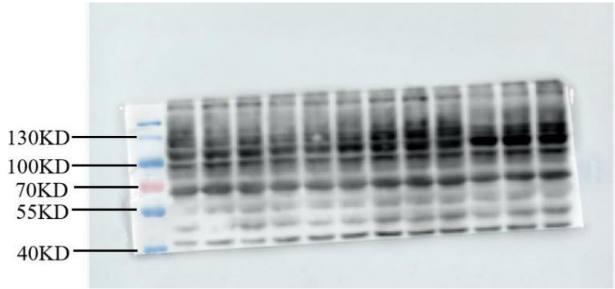

Membrane 14, Slice 2, probed with antibodies to **HO1**

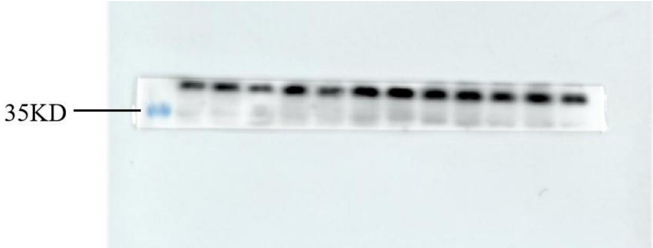

Membrane 14, Slice 2, probed with antibodies to **GAPDH**

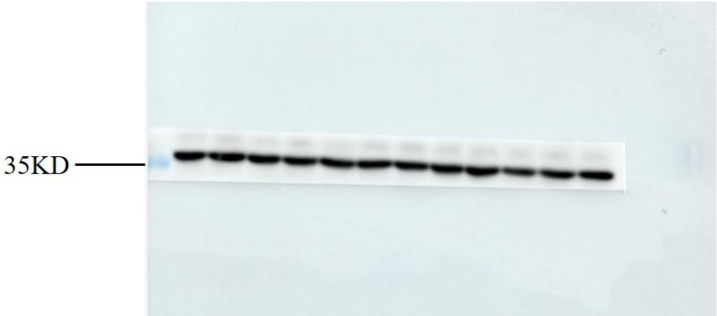

Membrane 14, Slice 3, probed with antibodies to **GPX4**

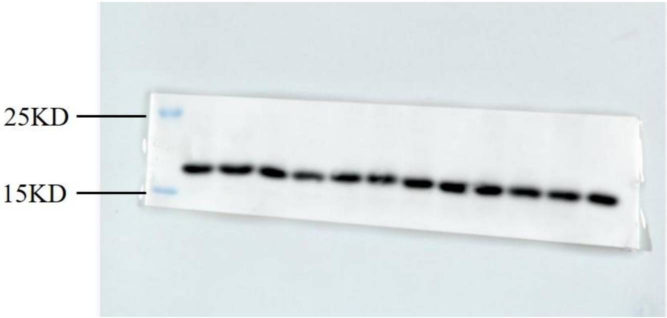

From left to right: *Fpn1*$^{flox/flox}$ Con 4, *Fpn1*$^{flox/flox}$ Con 5, *Fpn1*$^{flox/flox}$ Con 6, *Fpn1*$^{flox/flox}$ Ips 4, *Fpn1*$^{flox/flox}$ Ips 5, *Fpn1*$^{flox/flox}$ Ips 6, *Fpn1*^{cdh5}-CKO Con 4, *Fpn1*^{cdh5}-CKO Con 5, *Fpn1*^{cdh5}-CKO Con 6, *Fpn1*^{cdh5}-CKO Ips 4, *Fpn1*^{cdh5}-CKO Ips 5, *Fpn1*^{cdh5}-CKO Ips

Membrane 15, Slice 1, probed with antibodies to **Total Nrf2**

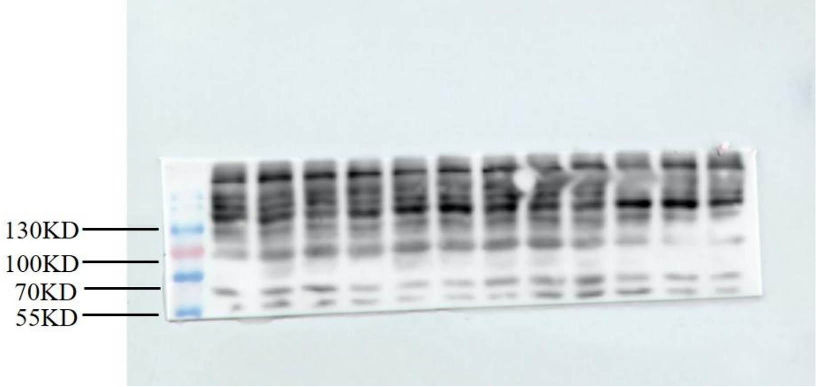

Membrane 15, Slice 2, probed with antibodies to **GAPDH**

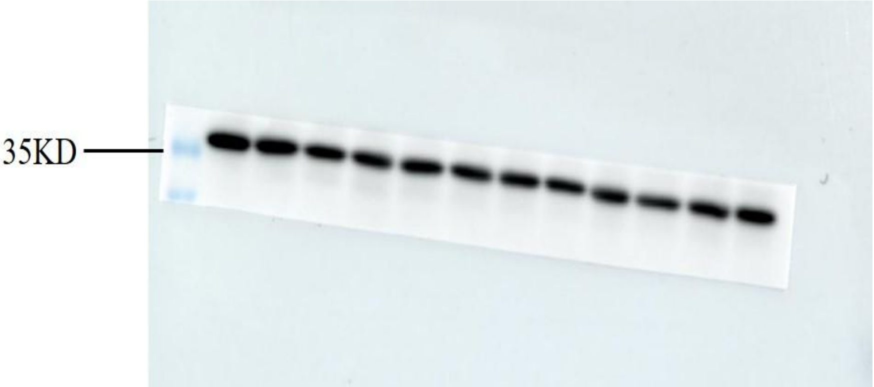

From left to right: *Fpn1*$^{flox/flox}$ Con 4, *Fpn1*$^{flox/flox}$ Con 5, *Fpn1*$^{flox/flox}$ Con 6, *Fpn1*$^{flox/flox}$ Ips 4, *Fpn1*$^{flox/flox}$ Ips 5, *Fpn1*$^{flox/flox}$ Ips 6, *Fpn1*^{cdh5}–CKO Con 4, *Fpn1*^{cdh5}–CKO Con 5, *Fpn1*^{cdh5}–CKO Con 6, *Fpn1*^{cdh5}–CKO Ips 4, *Fpn1*^{cdh5}–CKO Ips 5, *Fpn1*^{cdh5}–CKO Ips 6

Membrane 16, Slice 1, probed with antibodies to **HO1**

35KD

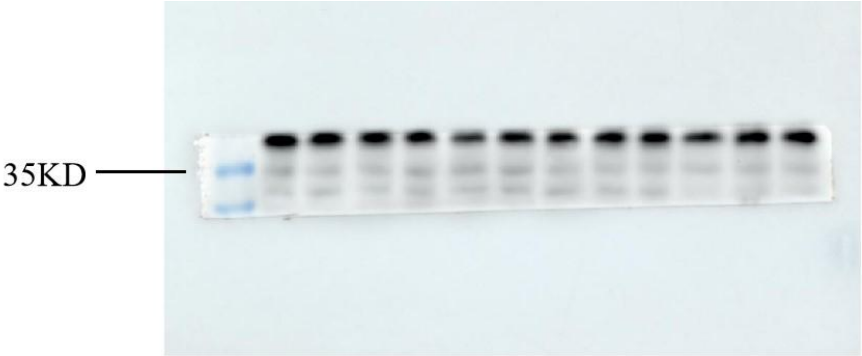

Membrane 16, Slice 1, probed with antibodies to **GAPDH**

35KD

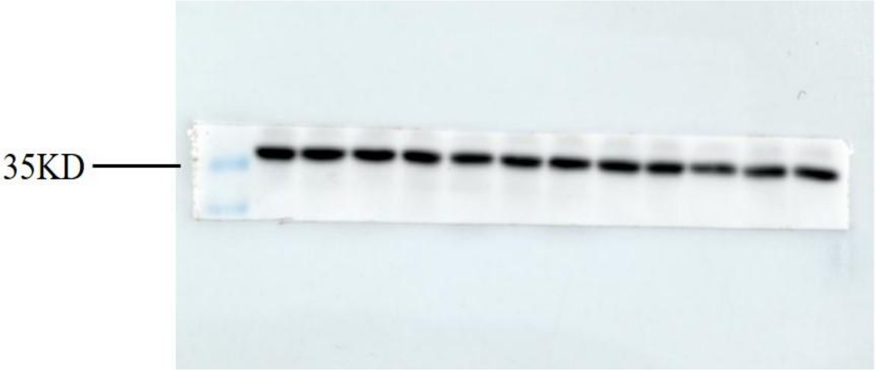

From left to right: *Fpn1*$^{flox/flox}$ Con 4, *Fpn1*$^{flox/flox}$ Con 5, *Fpn1*$^{flox/flox}$ Con 6, *Fpn1*$^{flox/flox}$ Ips 4, *Fpn1*$^{flox/flox}$ Ips 5, *Fpn1*$^{flox/flox}$ Ips 6, *Fpn1*^{cdh5}-CKO Con 4, *Fpn1*^{cdh5}-CKO Con 5, *Fpn1*^{cdh5}-CKO Con 6, *Fpn1*^{cdh5}-CKO Ips 4, *Fpn1*^{cdh5}-CKO Ips 5, *Fpn1*^{cdh5}-CKO Ips 6

Membrane 17, Slice 1, probed with antibodies to **GAPDH**

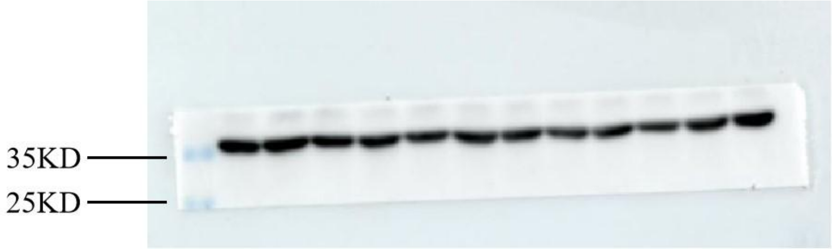

Membrane 17, Slice 2, probed with antibodies to **GPX4**

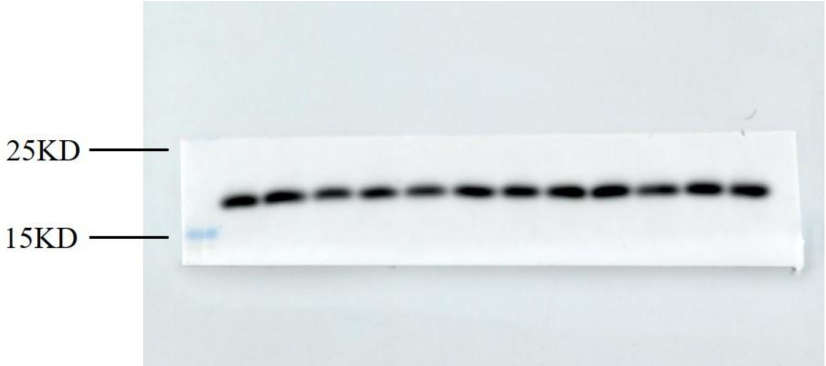

From left to right: *Fpn1*$^{flox/flox}$ Con 1, *Fpn1*$^{flox/flox}$ Con 2, *Fpn1*$^{flox/flox}$ Con 3, *Fpn1*$^{flox/flox}$ Ips 1, *Fpn1*$^{flox/flox}$ Ips 2, *Fpn1*$^{flox/flox}$ Ips 3, *Fpn1*^{cdh5}–CKO Con 1, *Fpn1*^{cdh5}–CKO Con 2, *Fpn1*^{cdh5}–CKO Con3, *Fpn1*^{cdh5}–CKO Ips 1, *Fpn1*^{cdh5}–CKO Ips 2, *Fpn1*^{cdh5}–CKO Ips 3

Membrane 18, Slice 1, probed with antibodies to **Cytoplasmic Nrf2**

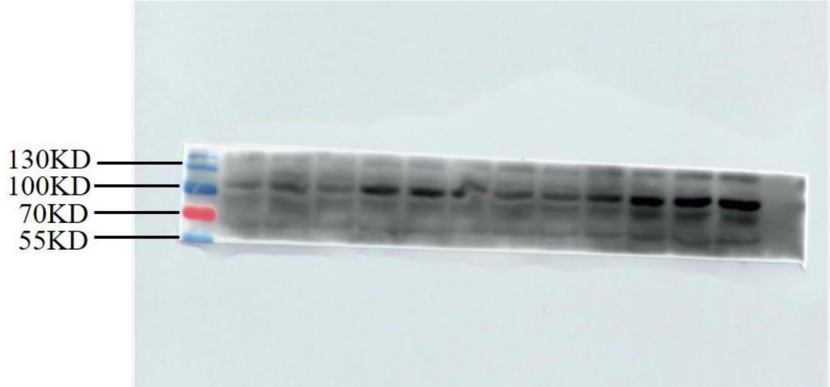

Membrane 18, Slice 2, probed with antibodies to **β-actin**

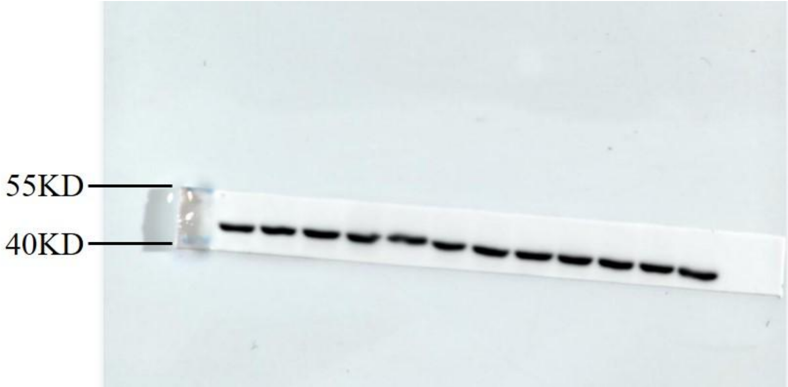

From left to right: *Fpn1*$^{flox/flox}$ Con 4, *Fpn1*$^{flox/flox}$ Con 5, *Fpn1*$^{flox/flox}$ Con 6, *Fpn1*$^{flox/flox}$ Ips 4, *Fpn1*$^{flox/flox}$ Ips 5, *Fpn1*$^{flox/flox}$ Ips 6, *Fpn1*^{cdh5}-CKO Con 4, *Fpn1*^{cdh5}-CKO Con 5, *Fpn1*^{cdh5}-CKO Con 6, *Fpn1*^{cdh5}-CKO Ips 4, *Fpn1*^{cdh5}-CKO Ips 5, *Fpn1*^{cdh5}-CKO Ips 6

Membrane 19, Slice 1, probed with antibodies to **Cytoplasmic Nrf2**

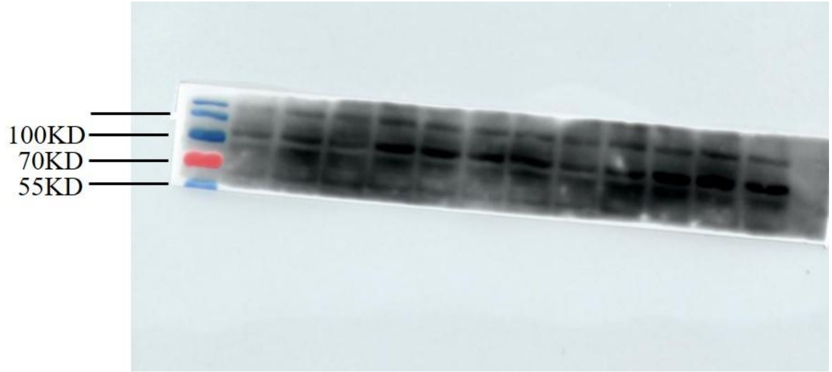

Membrane 19, Slice 2, probed with antibodies to **β-actin**

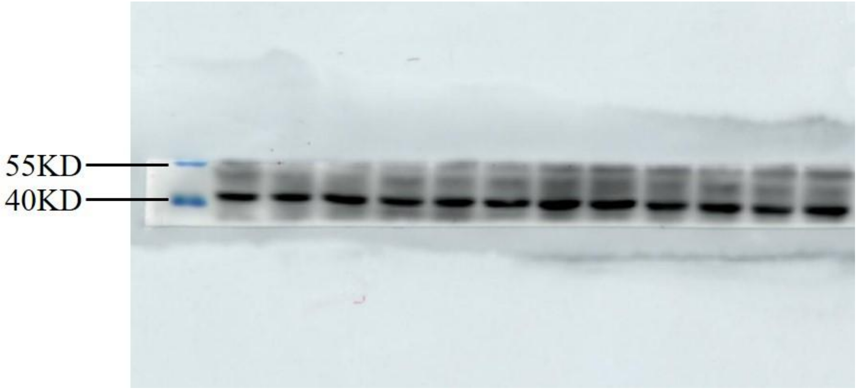

From left to right: *Fpn1*$^{flox/flox}$ Con 1, *Fpn1*$^{flox/flox}$ Con 2, *Fpn1*$^{flox/flox}$ Con 3, *Fpn1*$^{flox/flox}$ Ips 1, *Fpn1*$^{flox/flox}$ Ips 2, *Fpn1*$^{flox/flox}$ Ips 3, *Fpn1*^{cdh5}–CKO Con 1, *Fpn1*^{cdh5}–CKO Con 2, *Fpn1*^{cdh5}–CKO Con3, *Fpn1*^{cdh5}–CKO Ips 1, *Fpn1*^{cdh5}–CKO Ips 2, *Fpn1*^{cdh5}–CKO Ips 3

Membrane 20, Slice 1, probed with antibodies to **Nuclear Nrf2**

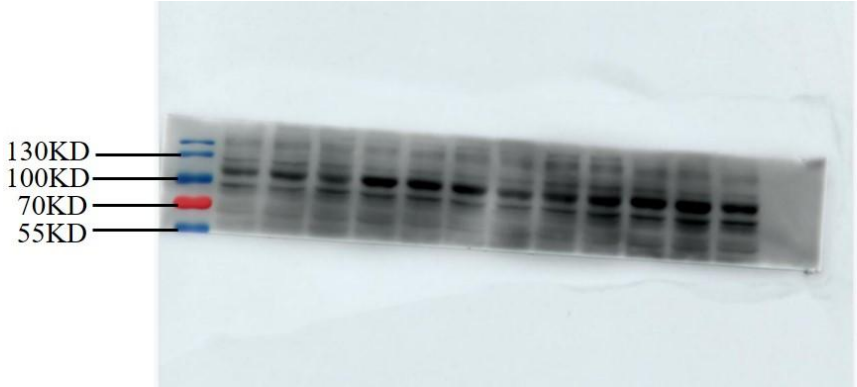

Membrane 20, Slice 2, probed with antibodies to **Histone**

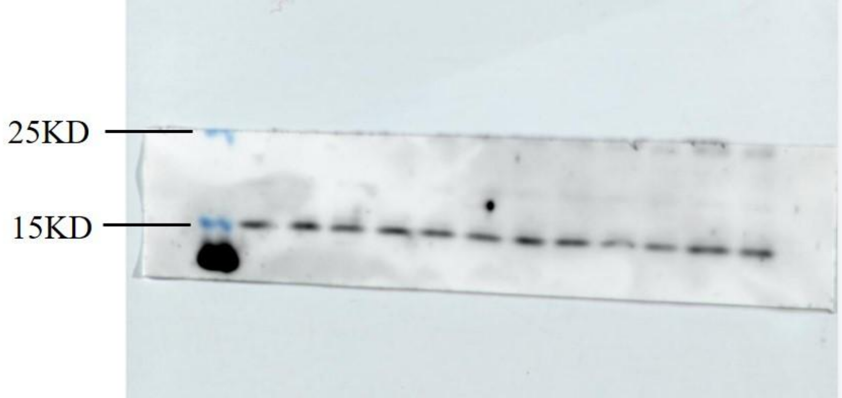

From left to right: *Fpn1*$^{\text{flox/flox}}$ Con 4, *Fpn1*$^{\text{flox/flox}}$ Con 5, *Fpn1*$^{\text{flox/flox}}$ Con 6, *Fpn1*$^{\text{flox/flox}}$ Ips 4, *Fpn1*$^{\text{flox/flox}}$ Ips 5, *Fpn1*$^{\text{flox/flox}}$ Ips 6, *Fpn1*$^{\text{cdh5}}$-CKO Con 4, *Fpn1*$^{\text{cdh5}}$-CKO Con 5, *Fpn1*$^{\text{cdh5}}$-CKO Con 6, *Fpn1*$^{\text{cdh5}}$-CKO Ips 4, *Fpn1*$^{\text{cdh5}}$-CKO Ips 5, *Fpn1*$^{\text{cdh5}}$-CKO Ips 6

Membrane 21, Slice 1, probed with antibodies to **Nuclear Nrf2**

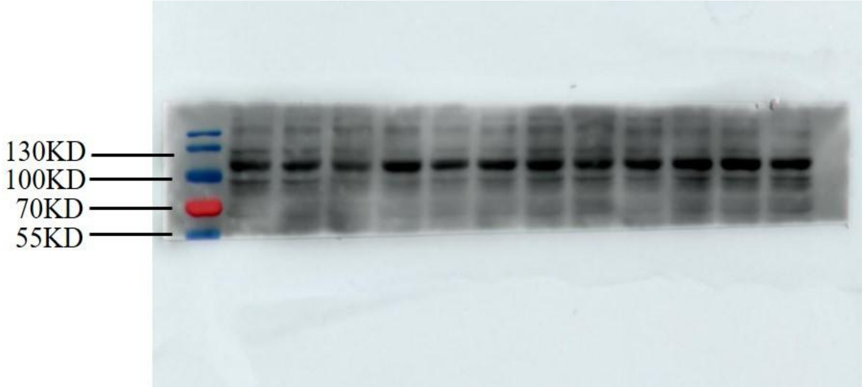

Membrane 21, Slice 2, probed with antibodies to **Histone**

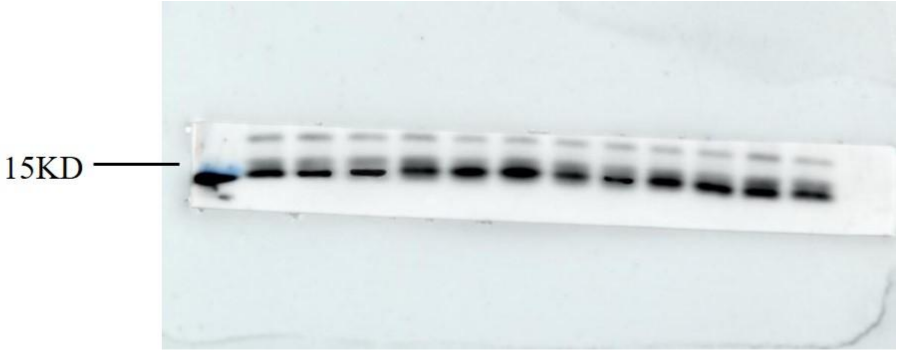

From left to right: *Fpn1*$^{flox/flox}$ Con 1, *Fpn1*$^{flox/flox}$ Con 2, *Fpn1*$^{flox/flox}$ Con 3, *Fpn1*$^{flox/flox}$ Ips 1, *Fpn1*$^{flox/flox}$ Ips 2, *Fpn1*$^{flox/flox}$ Ips 3, *Fpn1*^{cdh5}–CKO Con 1, *Fpn1*^{cdh5}–CKO Con 2, *Fpn1*^{cdh5}–CKO Con3, *Fpn1*^{cdh5}–CKO Ips 1, *Fpn1*^{cdh5}–CKO Ips 2, *Fpn1*^{cdh5}–CKO Ips 3

Membrane 22, Slice 1, probed with antibodies to **4HNE**

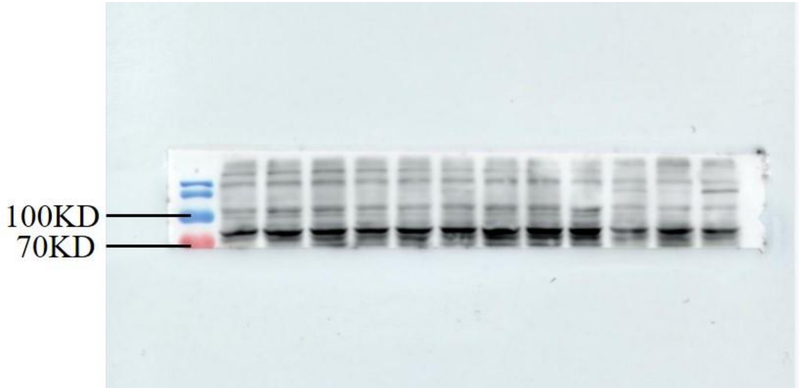

Membrane 22, Slice 2, probed with antibodies to **β-actin**

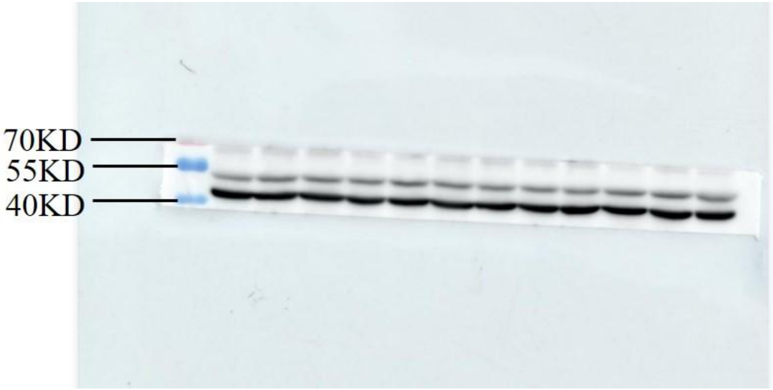

From left to right: *Fpn1*$^{flox/flox}$ Con 4, *Fpn1*$^{flox/flox}$ Con 5, *Fpn1*$^{flox/flox}$ Con 6, *Fpn1*$^{flox/flox}$ Ips 4, *Fpn1*$^{flox/flox}$ Ips 5, *Fpn1*$^{flox/flox}$ Ips 6, *Fpn1*^{cdh5}-CKO Con 4, *Fpn1*^{cdh5}-CKO Con 5, *Fpn1*^{cdh5}-CKO Con 6, *Fpn1*^{cdh5}-CKO Ips 4, *Fpn1*^{cdh5}-CKO Ips 5, *Fpn1*^{cdh5}-CKO Ips 6

Membrane 23, Slice 1, probed with antibodies to **4HNE**

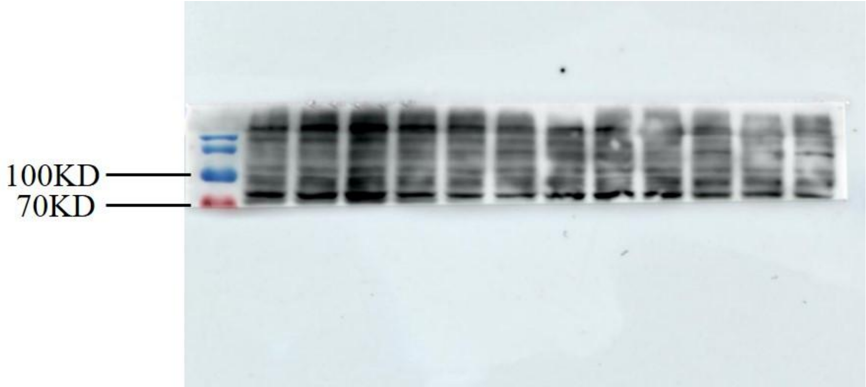

Membrane 23, Slice 2, probed with antibodies to **β-actin**

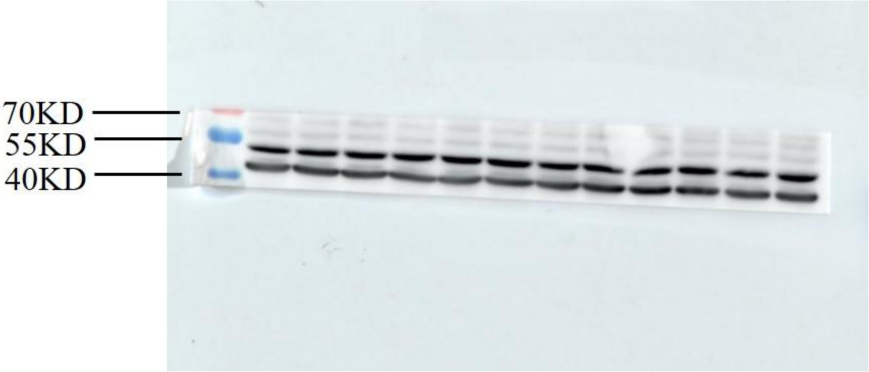

Supplement: Supplementary file 4 — Original Western Blots bands of Figure 3 [file 41419_2023_5688_MOESM4_ESM.pdf]
