# Supplementary material for: Cdh5-mediated Fpn1 deletion exerts neuroprotective effects during the acute phase and inhibitory effects during the recovery phase of ischemic stroke
Source: Cell Death Dis. 2023 Feb 25;14(2):161. doi: 10.1038/s41419-023-05688-1 (PMC9968354; doi:10.1038/s41419-023-05688-1)

Figure 5
Cerebral cortex

From left to right: *Fpn1*$^{flox/flox}$ Con 1, *Fpn1*$^{flox/flox}$ Con 2, *Fpn1*$^{flox/flox}$ Con 3, *Fpn1*$^{flox/flox}$ Ips 1, *Fpn1*$^{flox/flox}$ Ips 2, *Fpn1*$^{flox/flox}$ Ips 3, *Fpn1*^{cdh5}-CKO Con 1, *Fpn1*^{cdh5}-CKO Con 2, *Fpn1*^{cdh5}-CKO Con 3, *Fpn1*^{cdh5}-CKO Ips 1, *Fpn1*^{cdh5}-CKO Ips 2, *Fpn1*^{cdh5}-CKO Ips 3, *Fpn1*$^{flox/flox}$ Ips 4, *Fpn1*^{cdh5}-CKO Ips 4

Membrane 24, Slice 1, probed with antibodies to **β-actin**

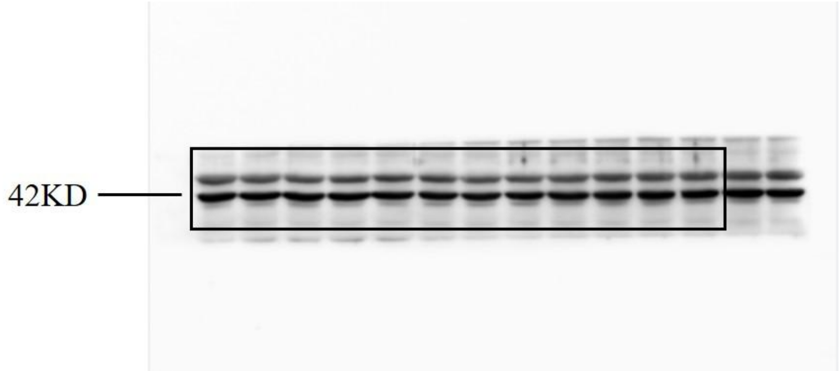

Membrane 24, Slice 2, probed with antibodies to **FtL**

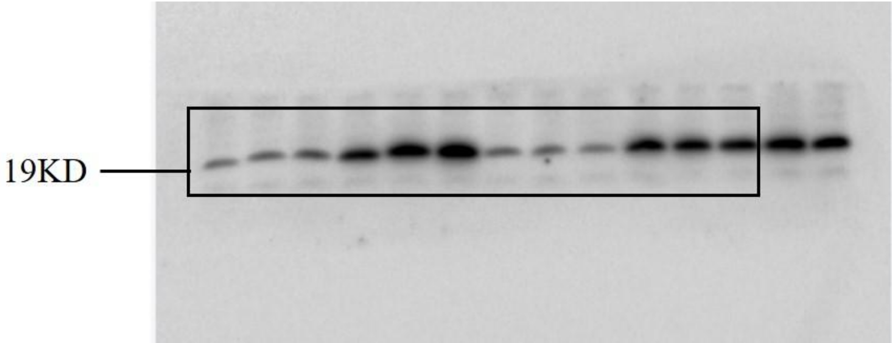

From left to right: *Fpn1*$^{flox/flox}$ Con 5, *Fpn1*$^{flox/flox}$ Con 6, *Fpn1*$^{flox/flox}$ Con 7, *Fpn1*$^{flox/flox}$ Ips 5, *Fpn1*$^{flox/flox}$ Ips 6, *Fpn1*$^{flox/flox}$ Ips 7, *Fpn1*^{cdh5}–CKO Con 5, *Fpn1*^{cdh5}–CKO Con 6, *Fpn1*^{cdh5}–CKO Con 7, *Fpn1*^{cdh5}–CKO Ips 5, *Fpn1*^{cdh5}–CKO Ips 6, *Fpn1*^{cdh5}–CKO Ips 7

Membrane 25, Slice 1, probed with antibodies to **β-actin**

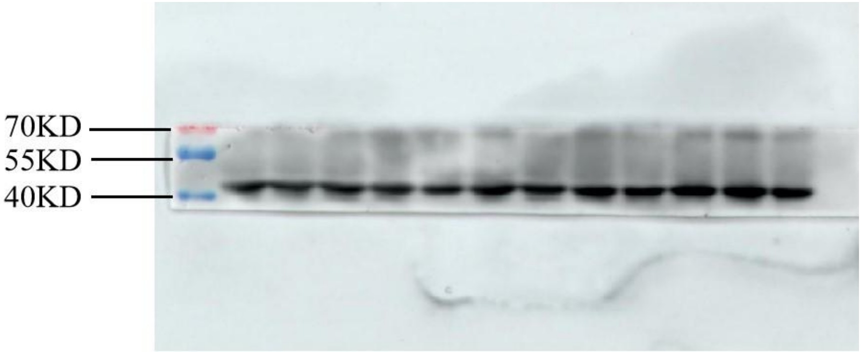

Membrane 25, Slice 2, probed with antibodies to **FtL**

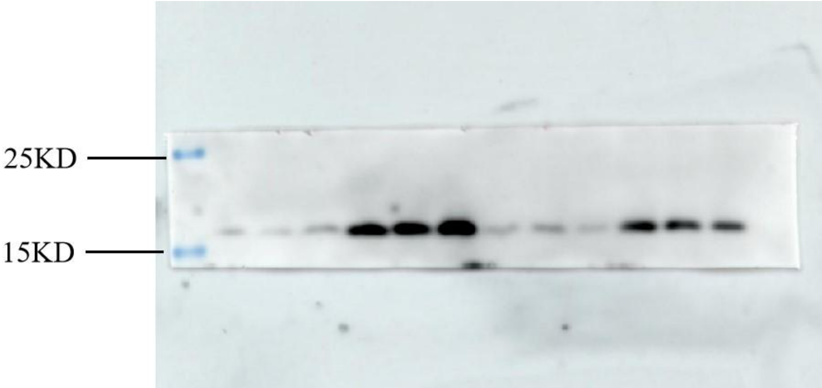

From left to right: *Fpn1*$^{flox/flox}$ Con 1, *Fpn1*$^{flox/flox}$ Con 2, *Fpn1*$^{flox/flox}$ Con 3, *Fpn1*$^{flox/flox}$ Ips 1, *Fpn1*$^{flox/flox}$ Ips 2, *Fpn1*$^{flox/flox}$ Ips 3, *Fpn1*^{cdh5}–CKO Con 1, *Fpn1*^{cdh5}–CKO Con 2, *Fpn1*^{cdh5}–CKO Con 3, *Fpn1*^{cdh5}–CKO Ips 1, *Fpn1*^{cdh5}–CKO Ips 2, *Fpn1*^{cdh5}–CKO Ips 3, *Fpn1*$^{flox/flox}$ Ips 4, *Fpn1*^{cdh5}–CKO Ips 4

Membrane 26, Slice 1, probed with antibodies to **TfR1**

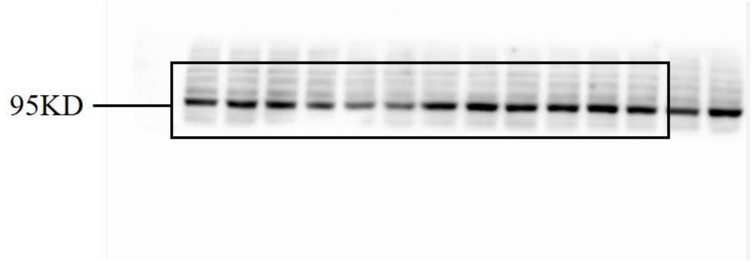

Membrane 26, Slice 2, probed with antibodies to **β-actin**

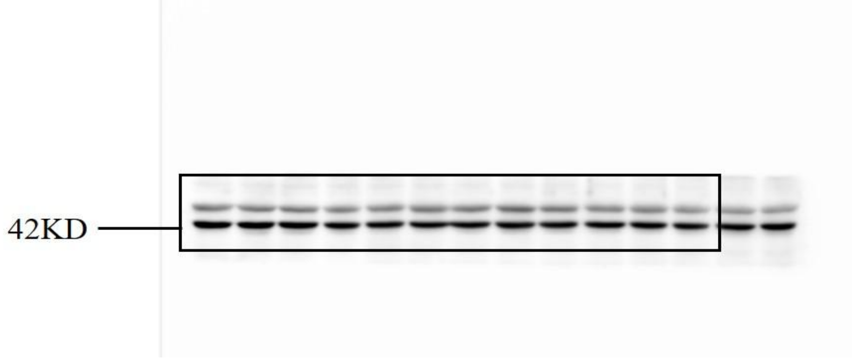

Membrane 26, Slice 3, probed with antibodies to **pre-hepcidin**

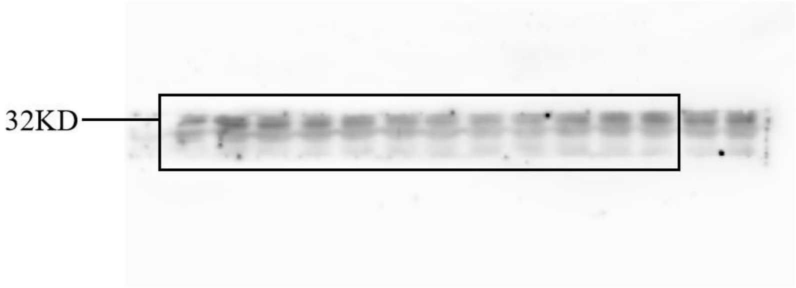

Membrane 26, Slice 4, probed with antibodies to **FtH**

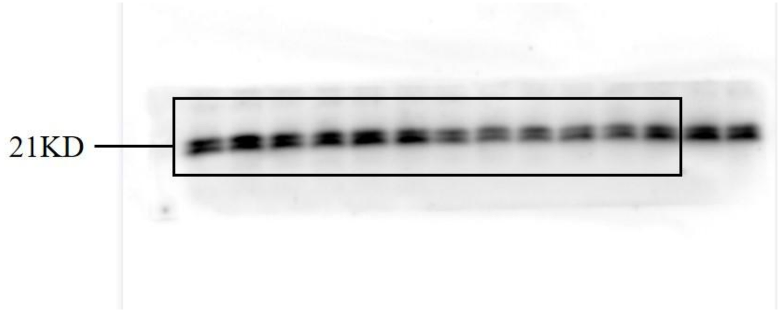

From left to right: *Fpn1*$^{flox/flox}$ Con 1, *Fpn1*$^{flox/flox}$ Con 2, *Fpn1*$^{flox/flox}$ Con 3, *Fpn1*$^{flox/flox}$ Ips 1, *Fpn1*$^{flox/flox}$ Ips 2, *Fpn1*$^{flox/flox}$ Ips 3, *Fpn1*^{cdh5}-CKO Con 1, *Fpn1*^{cdh5}-CKO Con 2, *Fpn1*^{cdh5}-CKO Con 3, *Fpn1*^{cdh5}-CKO Ips 1, *Fpn1*^{cdh5}-CKO Ips 2, *Fpn1*^{cdh5}-CKO Ips 3, *Fpn1*$^{flox/flox}$ Ips 4, *Fpn1*^{cdh5}-CKO Ips 4

Membrane 27, Slice 1, probed with antibodies to **FPN1**

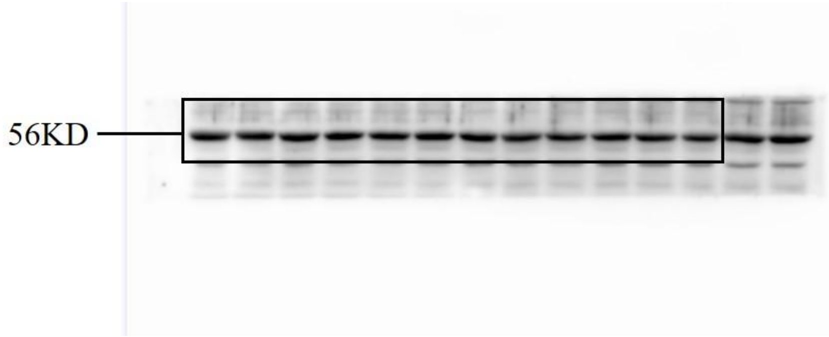

Membrane 27, Slice 2, probed with antibodies to **β-actin**

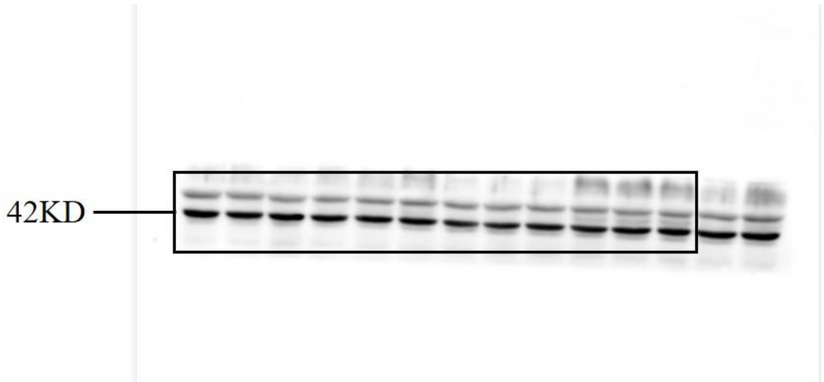

From left to right: *Fpn1*$^{flox/flox}$ Con 5, *Fpn1*$^{flox/flox}$ Con 6, *Fpn1*$^{flox/flox}$ Con 7, *Fpn1*$^{flox/flox}$ Ips 5, *Fpn1*$^{flox/flox}$ Ips 6, *Fpn1*$^{flox/flox}$ Ips 7, *Fpn1*^{cdh5}-CKO Con 5, *Fpn1*^{cdh5}-CKO Con 6, *Fpn1*^{cdh5}-CKO Con 7, *Fpn1*^{cdh5}-CKO Ips 5, *Fpn1*^{cdh5}-CKO Ips 6, *Fpn1*^{cdh5}-CKO Ips 7

Membrane 28, Slice 1, probed with antibodies to **TfR1**

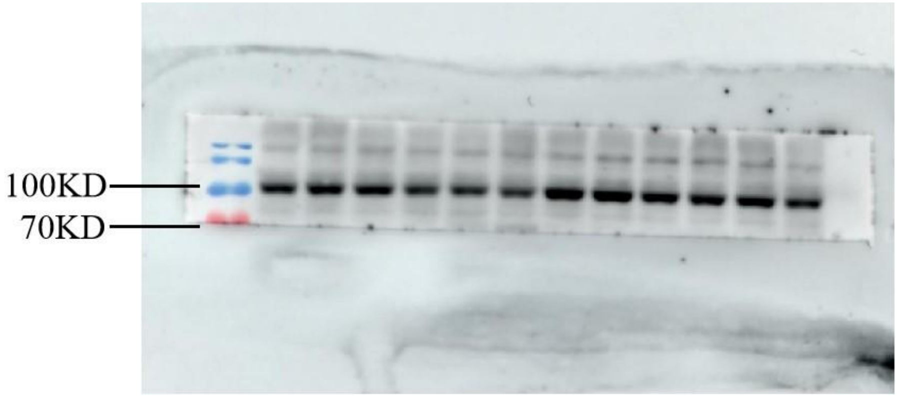

Membrane 28, Slice 2, probed with antibodies to **FPN1**

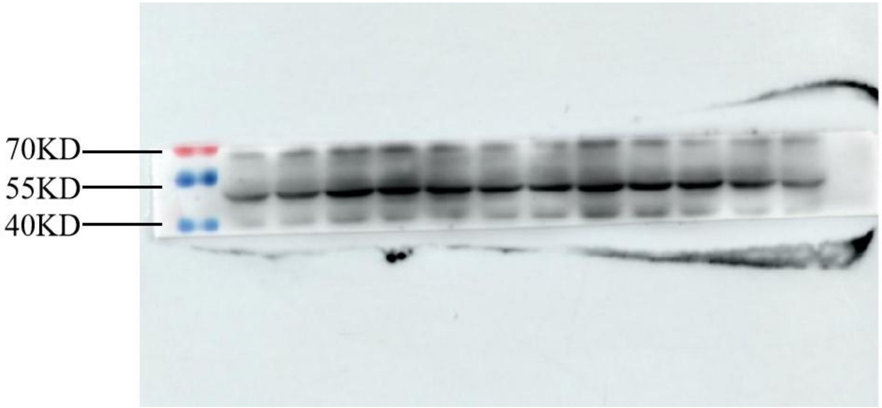

Membrane 28, Slice 2, probed with antibodies to **β-actin**

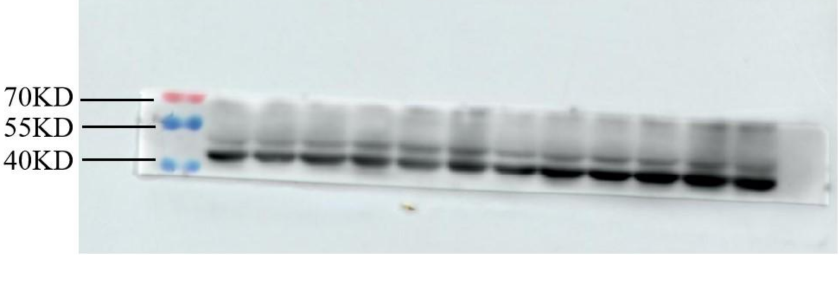

From left to right: *Fpn1*$^{flox/flox}$ Con 5, *Fpn1*$^{flox/flox}$ Con 6, *Fpn1*$^{flox/flox}$ Con 7, *Fpn1*$^{flox/flox}$ Ips 5, *Fpn1*$^{flox/flox}$ Ips 6, *Fpn1*$^{flox/flox}$ Ips 7, *Fpn1*^{cdh5}-CKO Con 5, *Fpn1*^{cdh5}-CKO Con 6, *Fpn1*^{cdh5}-CKO Con 7, *Fpn1*^{cdh5}-CKO Ips 5, *Fpn1*^{cdh5}-CKO Ips 6, *Fpn1*^{cdh5}-CKO Ips 7

Membrane 29, Slice 1, probed with antibodies to **β-actin**

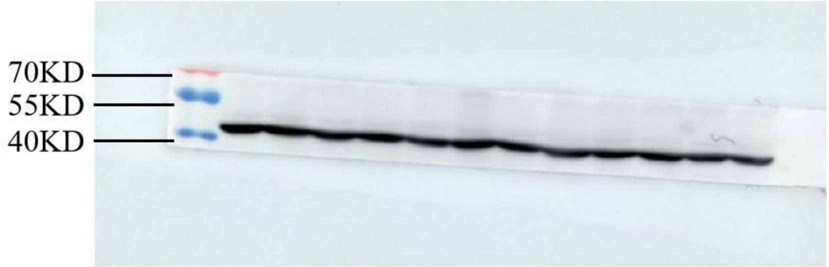

\

Membrane 29, Slice 2, probed with antibodies to **FtH**

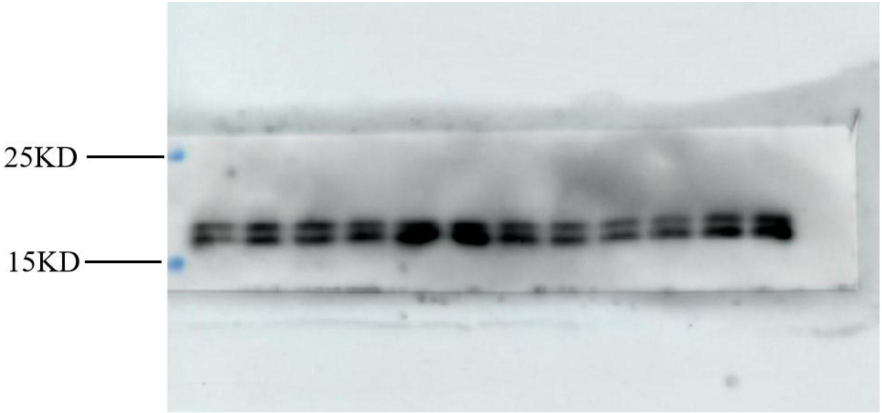

Supplement: Supplementary file 5 — Original Western Blots bands of Figure 5 [file 41419_2023_5688_MOESM5_ESM.pdf]
