# Supplementary material for: Cdh5-mediated Fpn1 deletion exerts neuroprotective effects during the acute phase and inhibitory effects during the recovery phase of ischemic stroke
Source: Cell Death Dis. 2023 Feb 25;14(2):161. doi: 10.1038/s41419-023-05688-1 (PMC9968354; doi:10.1038/s41419-023-05688-1)

Figure 7
Cerebral cortex

From left to right: *Fpn1*$^{flox/flox}$ Con 1, *Fpn1*$^{flox/flox}$ Con 2, *Fpn1*$^{flox/flox}$ Con 3, *Fpn1*$^{flox/flox}$ Ips 1, *Fpn1*$^{flox/flox}$ Ips 2, *Fpn1*$^{flox/flox}$ Ips 3, *Fpn1*^{cdh5}-CKO Con 1, *Fpn1*^{cdh5}-CKO Con 2, *Fpn1*^{cdh5}-CKO Con 3, *Fpn1*^{cdh5}-CKO Ips 1, *Fpn1*^{cdh5}-CKO Ips 2, *Fpn1*^{cdh5}-CKO Ips 3

Membrane 30, Slice 1, probed with antibodies to **Ki67**

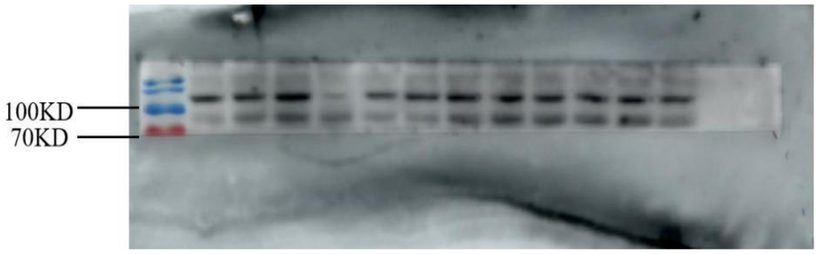

Membrane 30, Slice 2, probed with antibodies to **GFAP**

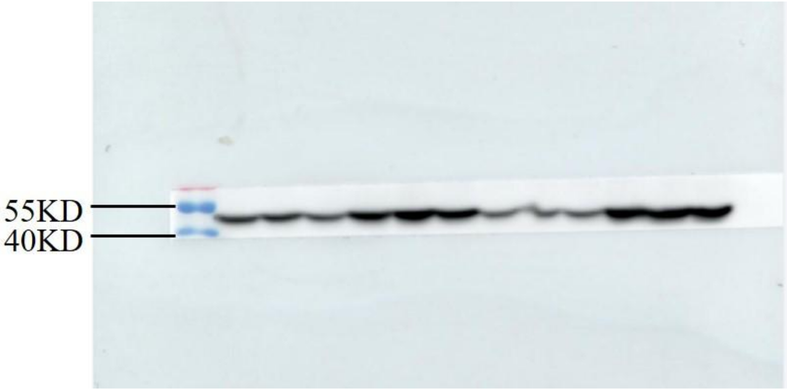

Membrane 30, Slice 3, probed with antibodies to **GAPDH**

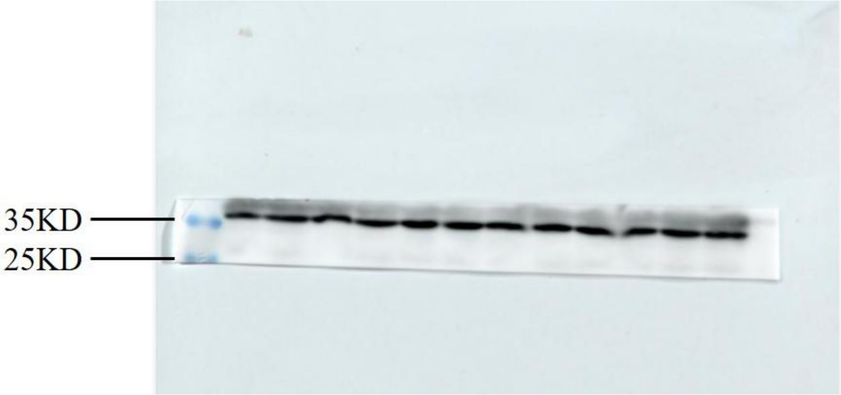

Membrane 30, Slice 4, probed with antibodies to **FtL**

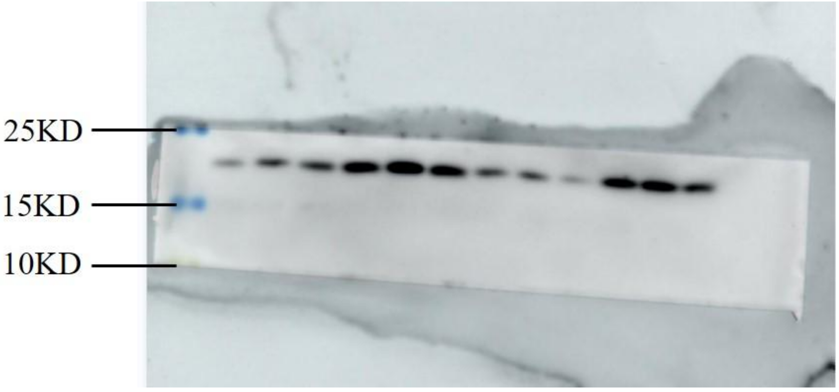

From left to right: *Fpn1*$^{flox/flox}$ Con 4, *Fpn1*$^{flox/flox}$ Con 5, *Fpn1*$^{flox/flox}$ Con 6, *Fpn1*$^{flox/flox}$ Ips 4, *Fpn1*$^{flox/flox}$ Ips 5, *Fpn1*$^{flox/flox}$ Ips 6, *Fpn1*^{cdh5}-CKO Con 4, *Fpn1*^{cdh5}-CKO Con 5, *Fpn1*^{cdh5}-CKO Con 6, *Fpn1*^{cdh5}-CKO Ips 4, *Fpn1*^{cdh5}-CKO Ips 5, *Fpn1*^{cdh5}-CKO Ips 6

Membrane 31, Slice 1, probed with antibodies to **GFAP**

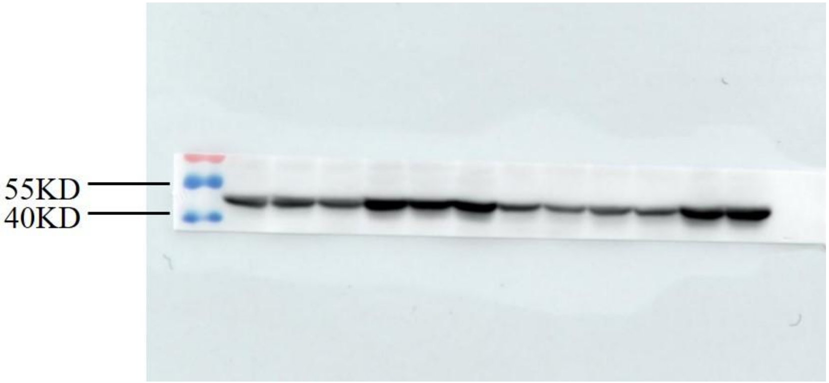

Membrane 31, Slice 2, probed with antibodies to **GAPDH**

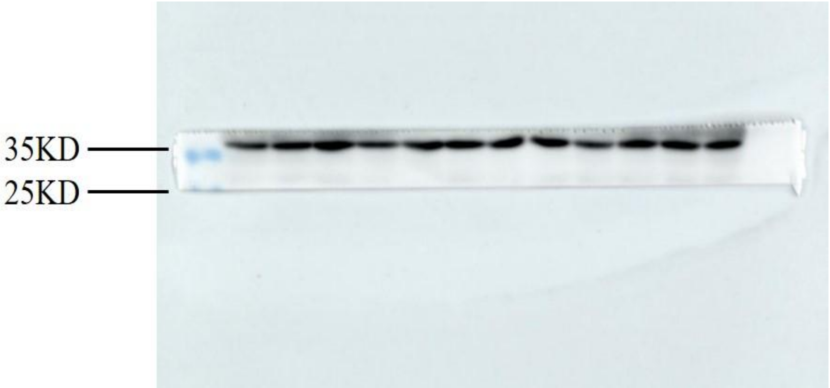

From left to right: *Fpn1*$^{flox/flox}$ Con 4, *Fpn1*$^{flox/flox}$ Con 5, *Fpn1*$^{flox/flox}$ Con 6, *Fpn1*$^{flox/flox}$ Ips 4, *Fpn1*$^{flox/flox}$ Ips 5, *Fpn1*$^{flox/flox}$ Ips 6, *Fpn1*^{cdh5}-CKO Con 4, *Fpn1*^{cdh5}-CKO Con 5, *Fpn1*^{cdh5}-CKO Con 6, *Fpn1*^{cdh5}-CKO Ips 4, *Fpn1*^{cdh5}-CKO Ips 5, *Fpn1*^{cdh5}-CKO Ips 6

Membrane 32, Slice 1, probed with antibodies to **GAPDH**

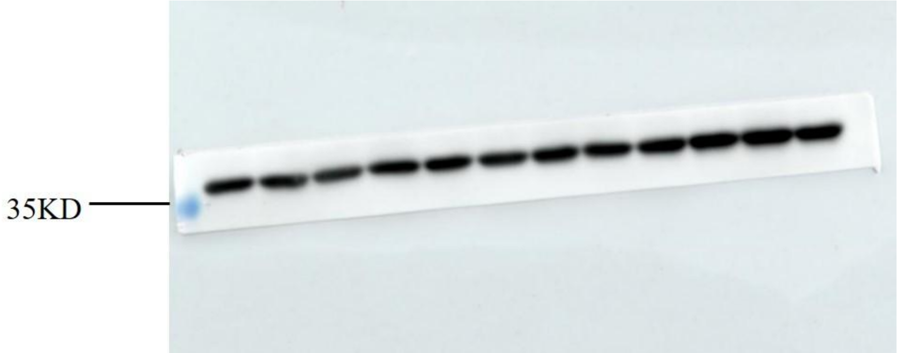

Membrane 32, Slice 2, probed with antibodies to **FtL**

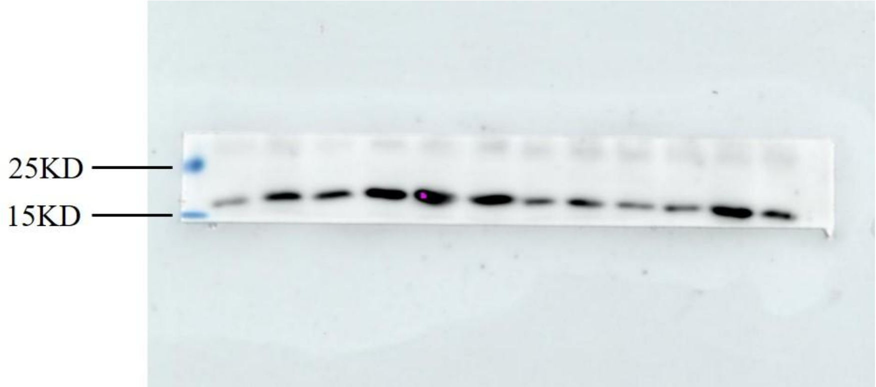

From left to right: $Fpn1^{flox/flox}$ Con 1, $Fpn1^{flox/flox}$ Con 2, $Fpn1^{flox/flox}$ Con 3, $Fpn1^{flox/flox}$ Ips 1, $Fpn1^{flox/flox}$ Ips 2, $Fpn1^{flox/flox}$ Ips 3, $Fpn1^{cdh5}$–CKO Con 1, $Fpn1^{cdh5}$–CKO Con 2, $Fpn1^{cdh5}$–CKO Con 3, $Fpn1^{cdh5}$–CKO Ips 1, $Fpn1^{cdh5}$–CKO Ips 2, $Fpn1^{cdh5}$–CKO Ips 3

Membrane 33, Slice 1, probed with antibodies to **GAPDH**

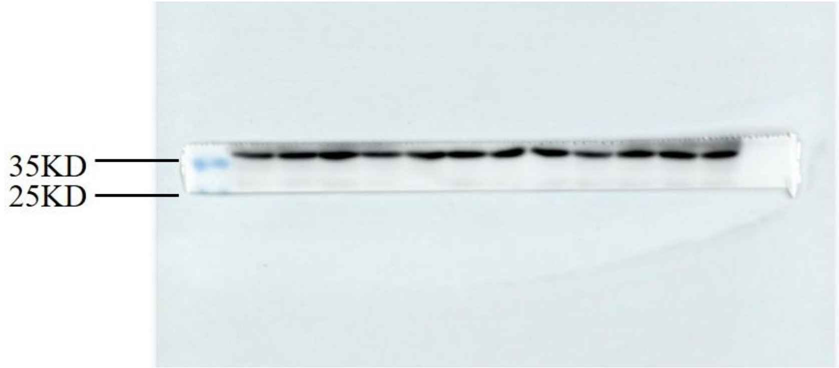

Membrane 33, Slice 2, probed with antibodies to **FtH**

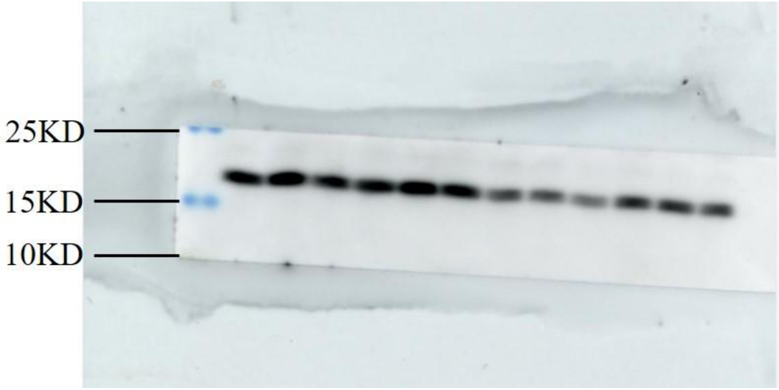

From left to right: *Fpn1*$^{flox/flox}$ Con 4, *Fpn1*$^{flox/flox}$ Con 5, *Fpn1*$^{flox/flox}$ Con 6, *Fpn1*$^{flox/flox}$ Ips 4, *Fpn1*$^{flox/flox}$ Ips 5, *Fpn1*$^{flox/flox}$ Ips 6, *Fpn1*^{cdh5}–CKO Con 4, *Fpn1*^{cdh5}–CKO Con 5, *Fpn1*^{cdh5}–CKO Con 6, *Fpn1*^{cdh5}–CKO Ips 4, *Fpn1*^{cdh5}–CKO Ips 5, *Fpn1*^{cdh5}–CKO Ips 6

Membrane 34, Slice 1, probed with antibodies to **GAPDH**

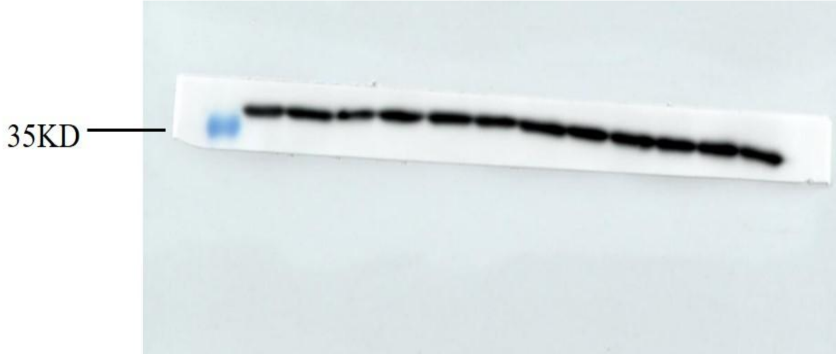

Membrane 34, Slice 2, probed with antibodies to **FtH**

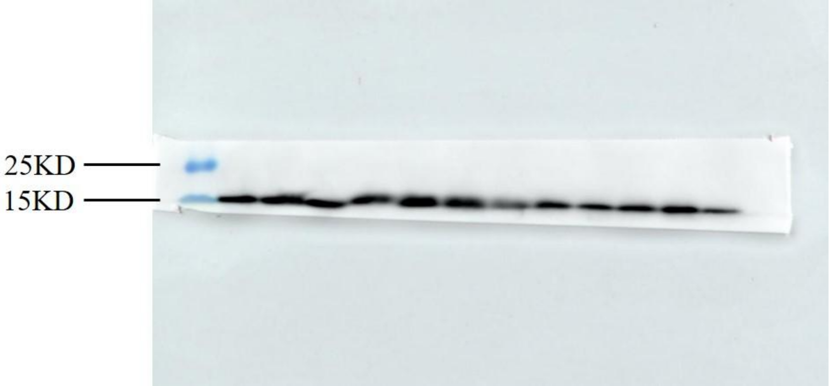

From left to right: *Fpn1*$^{flox/flox}$ Con 1, *Fpn1*$^{flox/flox}$ Con 2, *Fpn1*$^{flox/flox}$ Con 3, *Fpn1*$^{flox/flox}$ Ips 1, *Fpn1*$^{flox/flox}$ Ips 2, *Fpn1*$^{flox/flox}$ Ips 3, *Fpn1*^{cdh5}–CKO Con 1, *Fpn1*^{cdh5}–CKO Con 2, *Fpn1*^{cdh5}–CKO Con3, *Fpn1*^{cdh5}–CKO Ips 1, *Fpn1*^{cdh5}–CKO Ips 2, *Fpn1*^{cdh5}–CKO Ips 3

Membrane 35, Slice 1, probed with antibodies to **TfR1**

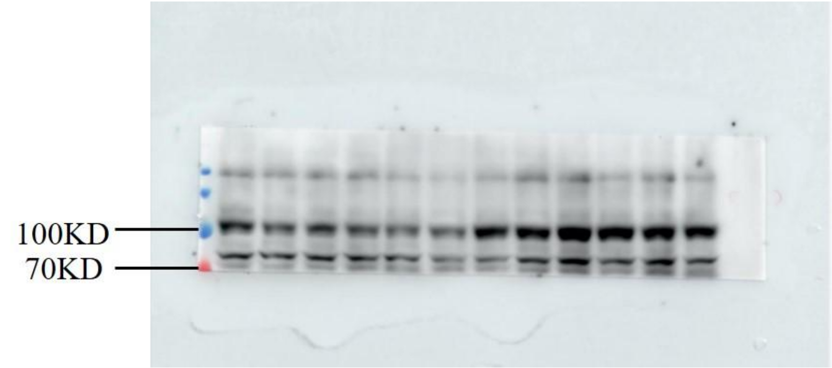

Membrane 35, Slice 2, probed with antibodies to **FPN1**

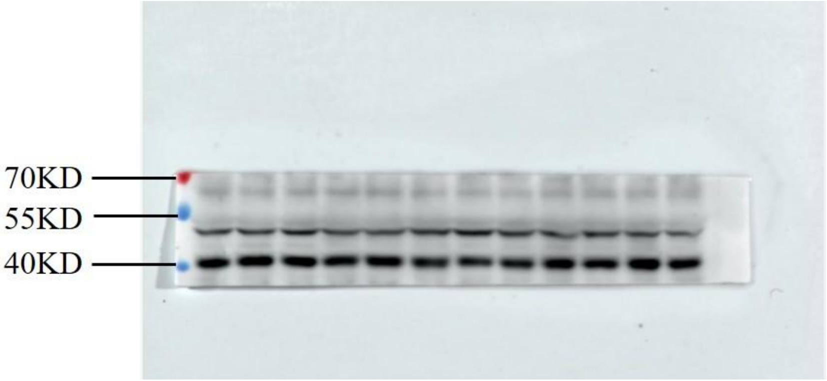

Membrane 35, Slice 3, probed with antibodies to **GAPDH**

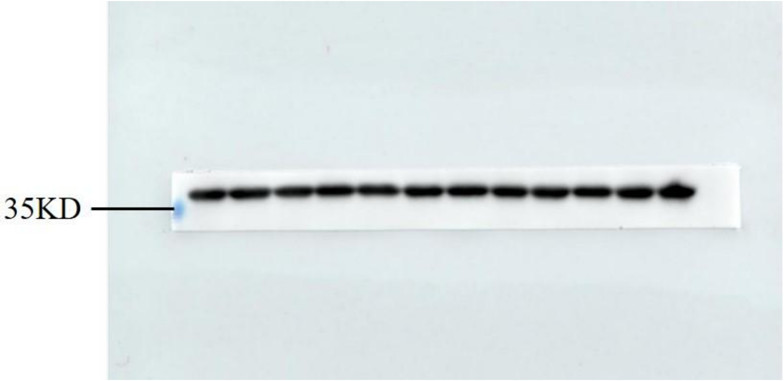

Membrane 35, **Slice 4**, probed with antibodies to **FtH**

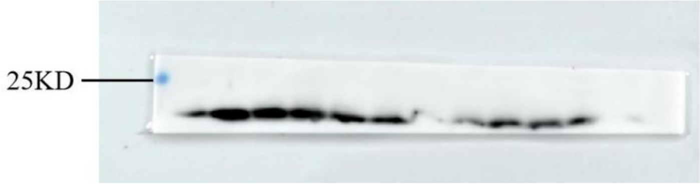

From left to right: *Fpn1*$^{flox/flox}$ Con 4, *Fpn1*$^{flox/flox}$ Con 5, *Fpn1*$^{flox/flox}$ Con 6, *Fpn1*$^{flox/flox}$ Ips 4, *Fpn1*$^{flox/flox}$ Ips 5, *Fpn1*$^{flox/flox}$ Ips 6, *Fpn1*^{cdh5}-CKO Con 4, *Fpn1*^{cdh5}-CKO Con 5, *Fpn1*^{cdh5}-CKO Con 6, *Fpn1*^{cdh5}-CKO Ips 4, *Fpn1*^{cdh5}-CKO Ips 5, *Fpn1*^{cdh5}-CKO Ips 6

Membrane 36, Slice 1, probed with antibodies to **TfR1**

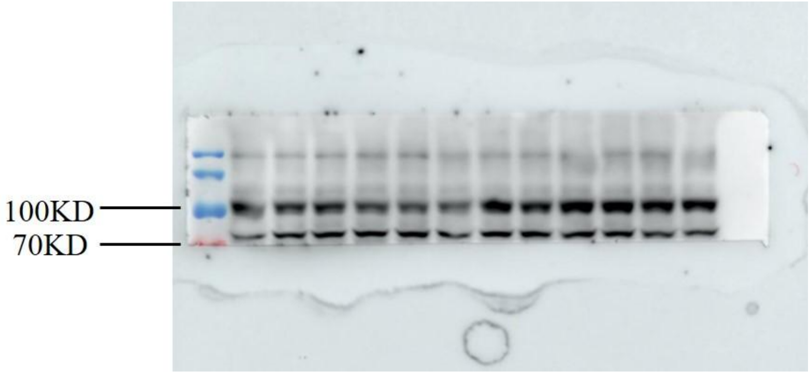

Membrane 36, Slice 2, probed with antibodies to **GAPDH**

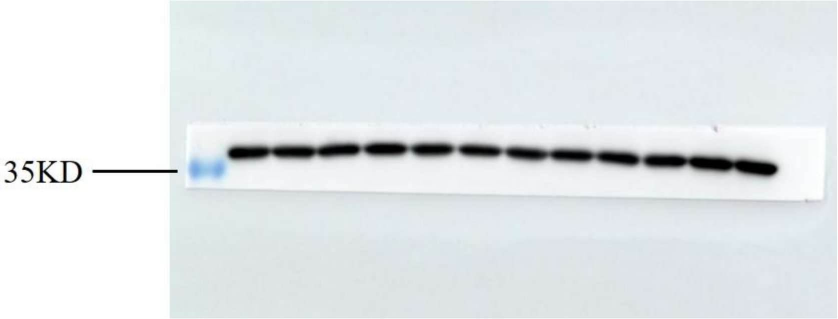

From left to right: *Fpn1*$^{flox/flox}$ Con 4, *Fpn1*$^{flox/flox}$ Con 5, *Fpn1*$^{flox/flox}$ Con 6, *Fpn1*$^{flox/flox}$ Ips 4, *Fpn1*$^{flox/flox}$ Ips 5, *Fpn1*$^{flox/flox}$ Ips 6, *Fpn1*^{cdh5}-CKO Con 4, *Fpn1*^{cdh5}-CKO Con 5, *Fpn1*^{cdh5}-CKO Con 6, *Fpn1*^{cdh5}-CKO Ips 4, *Fpn1*^{cdh5}-CKO Ips 5, *Fpn1*^{cdh5}-CKO Ips 6

Membrane 37, Slice 1, probed with antibodies to **FPN1**

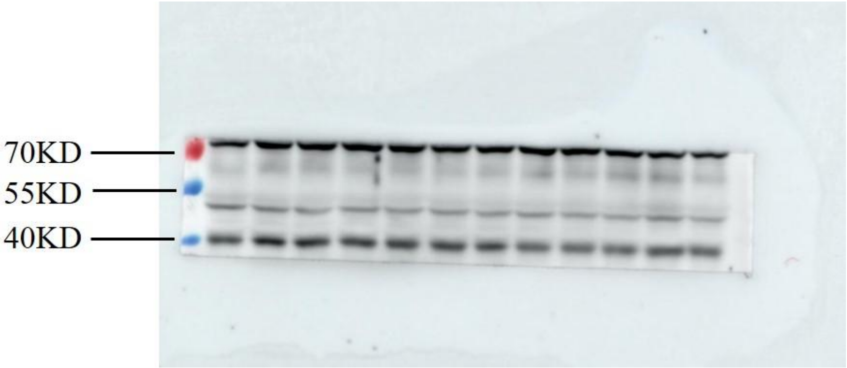

Membrane 37, Slice 2, probed with antibodies to **GAPDH**

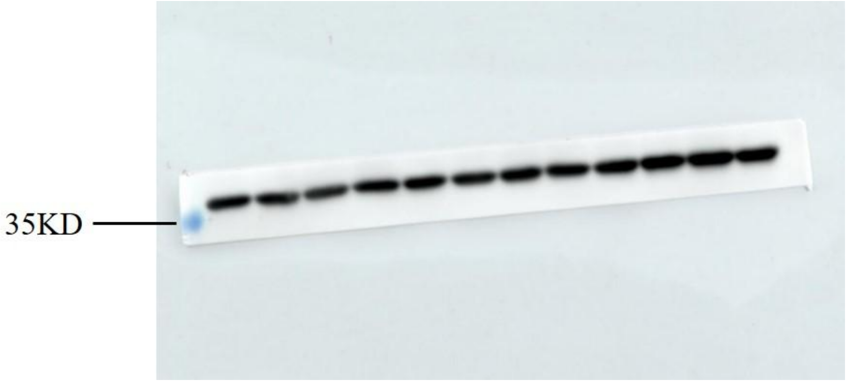

Supplement: Supplementary file 6 — Original Western Blots bands of Figure 7 [file 41419_2023_5688_MOESM6_ESM.pdf]
