# Supplementary material for: Cdh5-mediated Fpn1 deletion exerts neuroprotective effects during the acute phase and inhibitory effects during the recovery phase of ischemic stroke
Source: Cell Death Dis. 2023 Feb 25;14(2):161. doi: 10.1038/s41419-023-05688-1 (PMC9968354; doi:10.1038/s41419-023-05688-1)

Supplementary materials: full image scans of western blot membranes

Membrane 1, From left to right: *Fpn1*$^{flox/flox}$ Con 1, *Fpn1*$^{flox/flox}$ Con 2, *Fpn1*$^{flox/flox}$ Con 3, *Fpn1*$^{flox/flox}$ Ips 1, *Fpn1*$^{flox/flox}$ Ips 2, *Fpn1*$^{flox/flox}$ Ips 3, *Fpn1*^{cdh5}–CKO Con 1, *Fpn1*^{cdh5}–CKO Con 2, *Fpn1*^{cdh5}–CKO Con3, *Fpn1*^{cdh5}–CKO Ips 1, *Fpn1*^{cdh5}–CKO Ips 2, *Fpn1*^{cdh5}–CKO Ips 3

Slice 1, probed with antibodies to **TfR1** (Figure 2D in Submission CDDIS-22-2860R, and Figure 2A in revised manuscript)

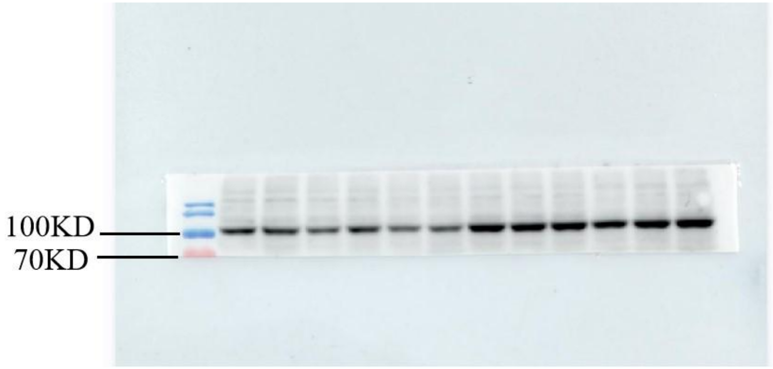

Slice 2, probed with antibodies to **β-actin** (Figures 2A and 2D in Submission CDDIS-22-2860R, and Figure 2A in revised manuscript)

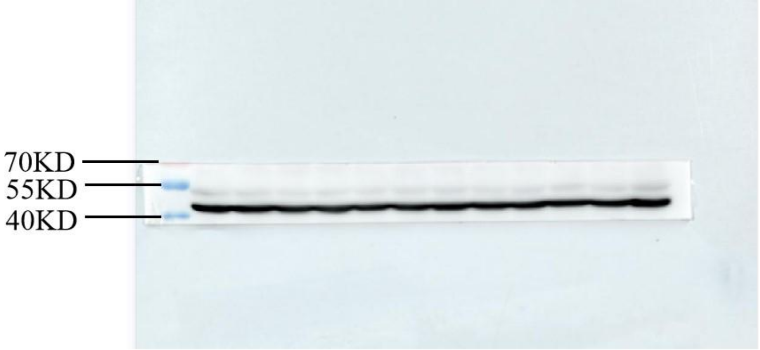

Slice 3, probed with antibodies to **GAPDH** (not provided in manuscript)

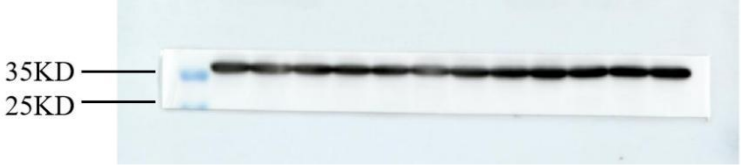

Slice 4, probed with antibodies to **FtL (**Figure 2A in Submission CDDIS-22-2860R, and Figure 2A in revised manuscript)

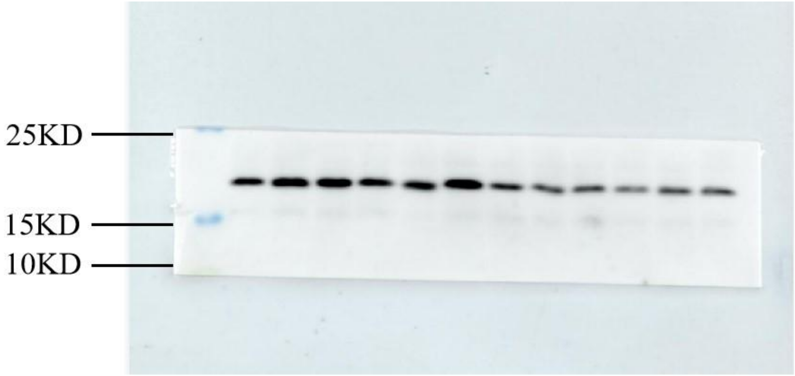

Membrane 5, From left to right: *Fpn1*$^{flox/flox}$ Con 1, *Fpn1*$^{flox/flox}$ Con 2, *Fpn1*$^{flox/flox}$ Con 3, *Fpn1*$^{flox/flox}$ Ips 1, *Fpn1*$^{flox/flox}$ Ips 2, *Fpn1*$^{flox/flox}$ Ips 3, *Fpn1*^{cdh5}-CKO Con 1, *Fpn1*^{cdh5}-CKO Con 2, *Fpn1*^{cdh5}-CKO Con3, *Fpn1*^{cdh5}-CKO Ips 1, *Fpn1*^{cdh5}-CKO Ips 2, *Fpn1*^{cdh5}-CKO Ips 3

Slice 1, probed with antibodies to **4HNE** (not provided in manuscript)

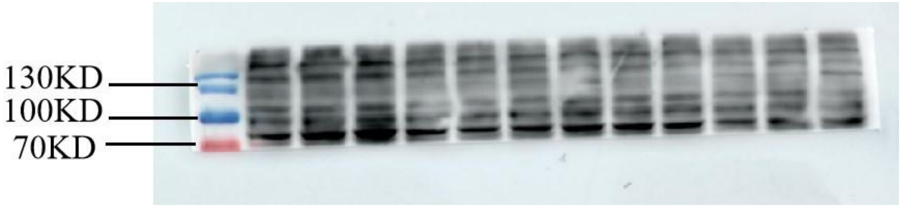

Slice 2, probed with antibodies to **FPN1** (Figure 2D in Submission CDDIS-22-2860R, and Figure 2A in revised manuscript)

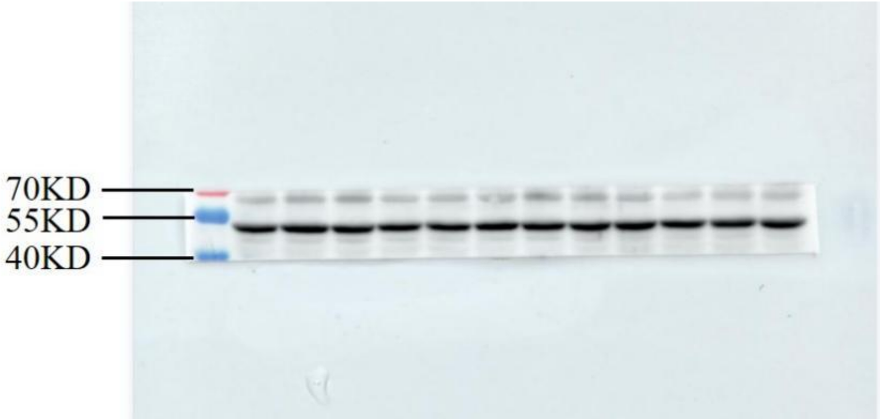

Slice 2, After eluting slice 2 with primary and secondary antibody (Sevenbio, beijing, China), the slice was blocked in 5% skim milk. The slice was then reprobed with antibodies to **β-actin** (Figures 2D and 4D in Submission CDDIS-22-2860R, and Figure 2A in revised manuscript)

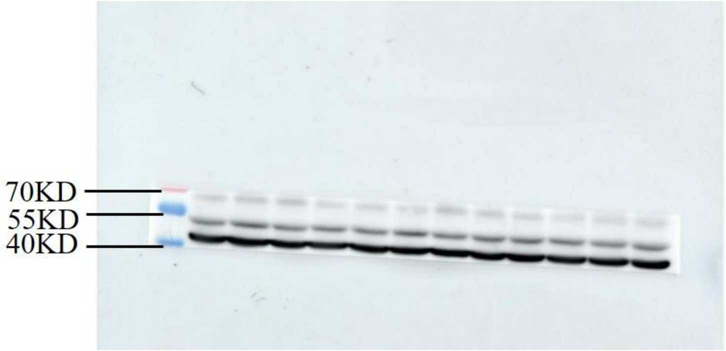

Slice 3, probed with antibodies to **GAPDH** (not provided in submission CDDIS-22-2860R, and present in Figure 2E as a loading control of Bcl2 in revised manuscript)

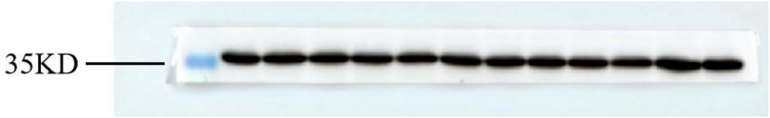

Slice 4, probed with antibodies to **Bcl2** (Figure 4D in Submission CDDIS-22-2860R, and Figure2E in revised manuscript)

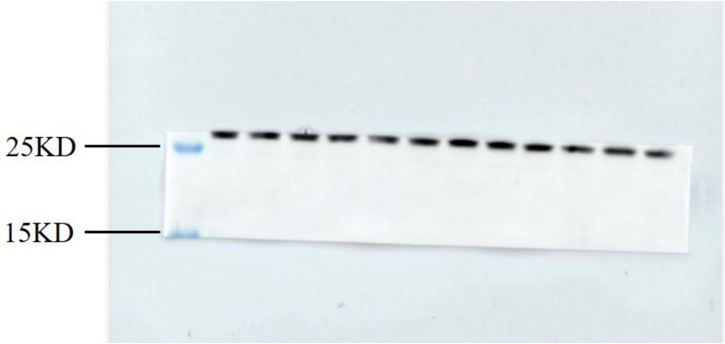

Membrane 14, From left to right: *Fpn1*$^{flox/flox}$ Con 1, *Fpn1*$^{flox/flox}$ Con 2, *Fpn1*$^{flox/flox}$ Con 3, *Fpn1*$^{flox/flox}$ Ips 1, *Fpn1*$^{flox/flox}$ Ips 2, *Fpn1*$^{flox/flox}$ Ips 3, *Fpn1*^{cdh5}-CKO Con 1, *Fpn1*^{cdh5}-CKO Con 2, *Fpn1*^{cdh5}-CKO Con3, *Fpn1*^{cdh5}-CKO Ips 1, *Fpn1*^{cdh5}-CKO Ips 2, *Fpn1*^{cdh5}-CKO Ips 3

Slice 1, probed with antibodies to **Total Nrf2** (Figure 3A in Submission CDDIS-22-2860R, and Figure 3A in revised manuscript)

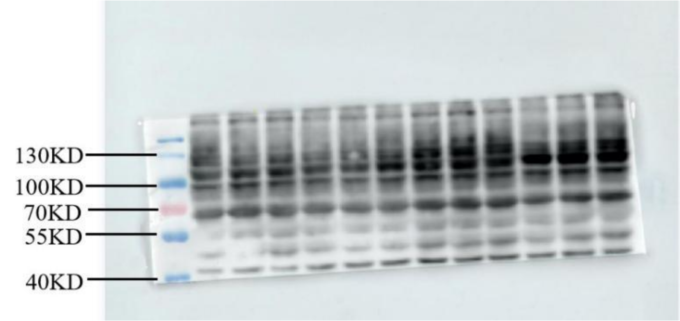

Slice 2, probed with antibodies to **HO1** (Figure 3E in Submission CDDIS-22-2860R, and Figure 3A in revised manuscript)

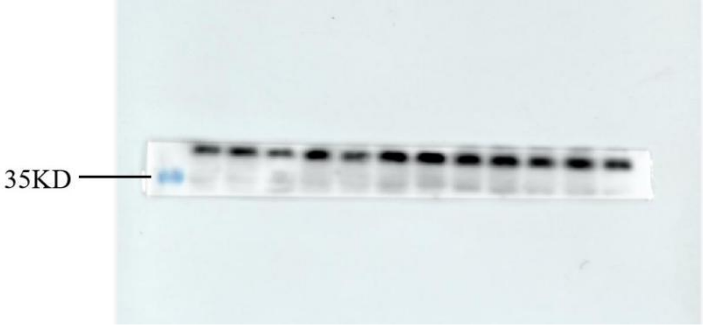

Slice 2, After eluting slice 2 with primary and secondary antibody eluent (Sevenbio, beijing, China) , the slice was blocked in 5% skim milk. The slice was then reprobed with antibodies to **GAPDH** (Figures 3A, E and 4A in Submission CDDIS-22-2860R, and Figure 3A in revised manuscript).

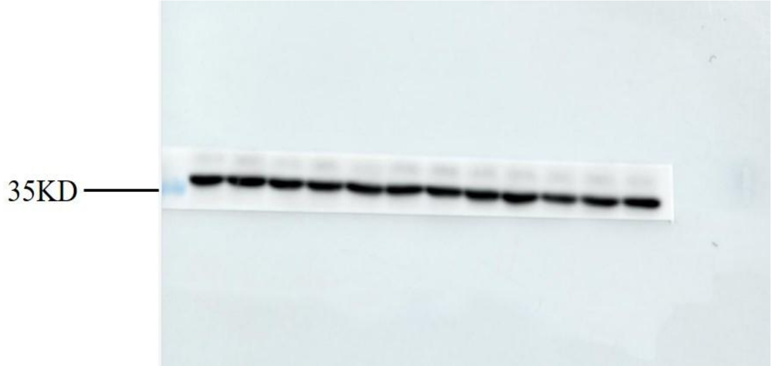

Slice 3, **GPX4** (Figure 4A in Submission CDDIS-22-2860R, and Figure 3A in revised manuscript).

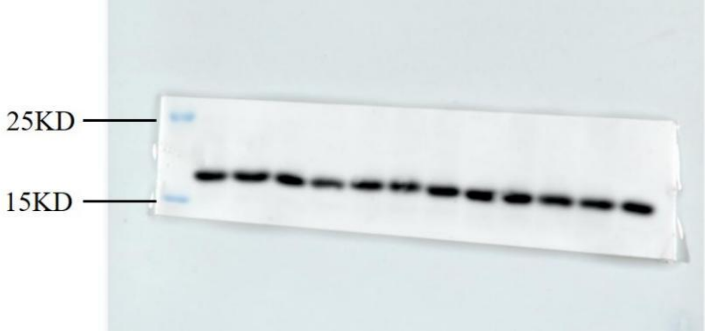

Membrane 26, From left to right: *Fpn1*$^{flox/flox}$ Con 1, *Fpn1*$^{flox/flox}$ Con 2, *Fpn1*$^{flox/flox}$ Con 3, *Fpn1*$^{flox/flox}$ Ips 1, *Fpn1*$^{flox/flox}$ Ips 2, *Fpn1*$^{flox/flox}$ Ips 3, *Fpn1*^{cdh5}-CKO Con 1, *Fpn1*^{cdh5}-CKO Con 2, *Fpn1*^{cdh5}-CKO Con3, *Fpn1*^{cdh5}-CKO Ips 1, *Fpn1*^{cdh5}-CKO Ips 2, *Fpn1*^{cdh5}-CKO Ips 3

Slice 1, probed with antibodies to **TfR1** (Figure 6B in Submission CDDIS-22-2860R, and Figure 5B in revised manuscript)

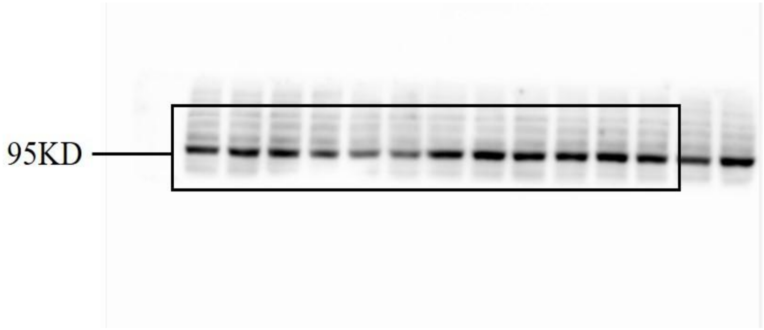

Slice 2, probed with antibodies to **β-actin** (Figure 6B in Submission CDDIS-22-2860R, and Figure 5B in revised manuscript)

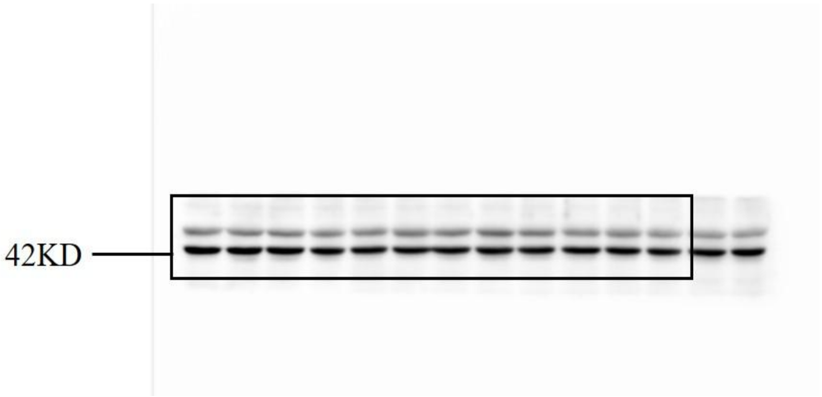

Slice 3, probed with antibodies to **pre-hepcidin** (not provided in manuscript)

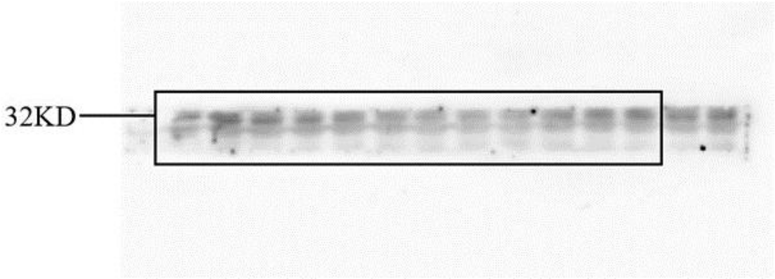

Slice 4, probed with antibodies to **FtH** (Figure 6B in Submission CDDIS-22-2860R, and Figure 5B in revised manuscript)

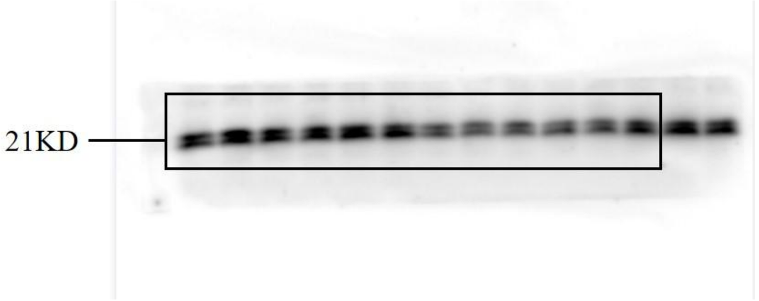

Membrane 30, From left to right: *Fpn1*$^{flox/flox}$ Con 1, *Fpn1*$^{flox/flox}$ Con 2, *Fpn1*$^{flox/flox}$ Con 3, *Fpn1*$^{flox/flox}$ Ips 1, *Fpn1*$^{flox/flox}$ Ips 2, *Fpn1*$^{flox/flox}$ Ips 3, *Fpn1*^{cdh5}-CKO Con 1, *Fpn1*^{cdh5}-CKO Con 2, *Fpn1*^{cdh5}-CKO Con3, *Fpn1*^{cdh5}-CKO Ips 1, *Fpn1*^{cdh5}-CKO Ips 2, *Fpn1*^{cdh5}-CKO Ips 3

Slice 1, probed with antibodies to **Ki67** (not provided in Manuscript)

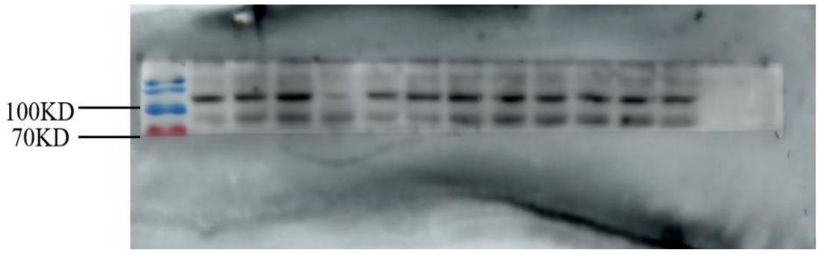

Slice 2, probed with antibodies to **GFAP** (Figure 9A in Submission CDDIS-22-2860R, and Figure 7B in revised manuscript)

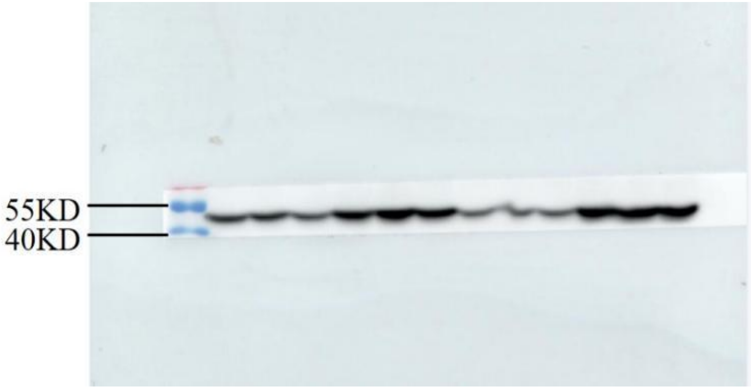

Slice 3, probed with antibodies to **GAPDH** (Figures 8B and 9A in Submission CDDIS-22-2860R, and Figure 7B in revised manuscript)

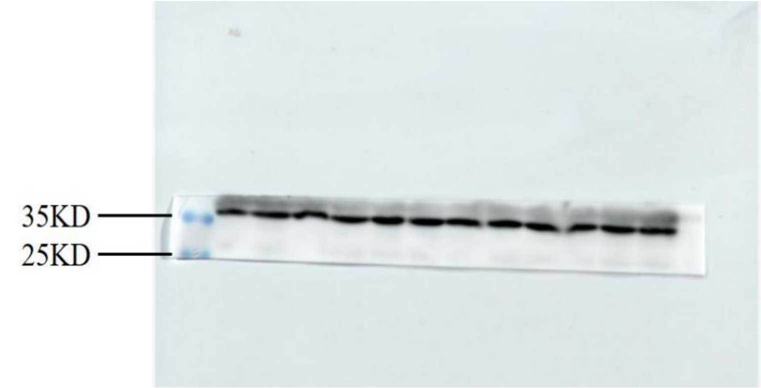

Slice 4, probed with antibodies to **FtL** (Figure 8B in Submission CDDIS-22-2860R, and Figure 7B in revised manuscript)

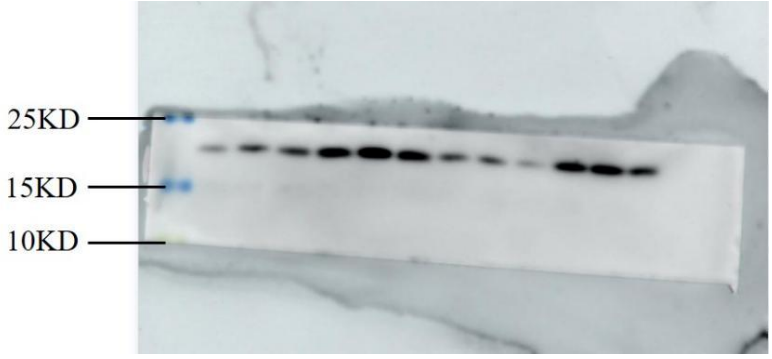

Membrane 35, From left to right: *Fpn1*$^{\mathrm{flox/flox}}$ Con 1, *Fpn1*$^{\mathrm{flox/flox}}$ Con 2, *Fpn1*$^{\mathrm{flox/flox}}$ Con 3, *Fpn1*$^{\mathrm{flox/flox}}$ Ips 1, *Fpn1*$^{\mathrm{flox/flox}}$ Ips 2, *Fpn1*$^{\mathrm{flox/flox}}$ Ips 3, *Fpn1*$^{\mathrm{cdh5}}$-CKO Con 1, *Fpn1*$^{\mathrm{cdh5}}$-CKO Con 2, *Fpn1*$^{\mathrm{cdh5}}$-CKO Con3, *Fpn1*$^{\mathrm{cdh5}}$-CKO Ips 1, *Fpn1*$^{\mathrm{cdh5}}$-CKO Ips 2, *Fpn1*$^{\mathrm{cdh5}}$-CKO Ips 3

Slice 1, probed with antibodies to **TfR1** (Figure 8B in Submission CDDIS-22-2860R, and Figure 7B in revised manuscript)

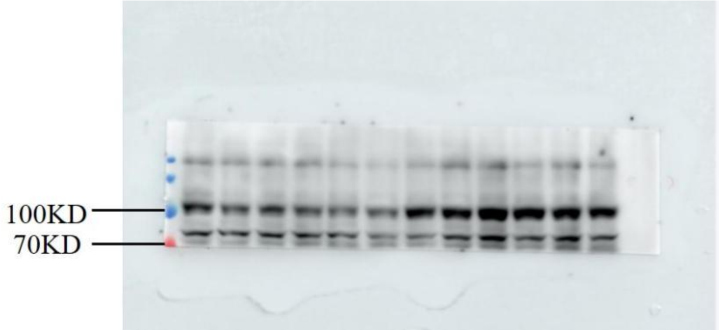

Slice 2, probed with antibodies to **FPN1** (Figure 8B in Submission CDDIS-22-2860R, and Figure 7B in revised manuscript)

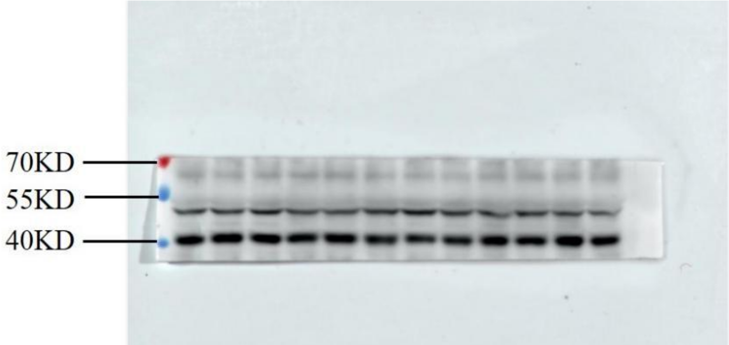

Slice 3, probed with antibodies to **GAPDH** (Figure 8B in Submission CDDIS-22-2860R, and Figure 7B in revised manuscript)

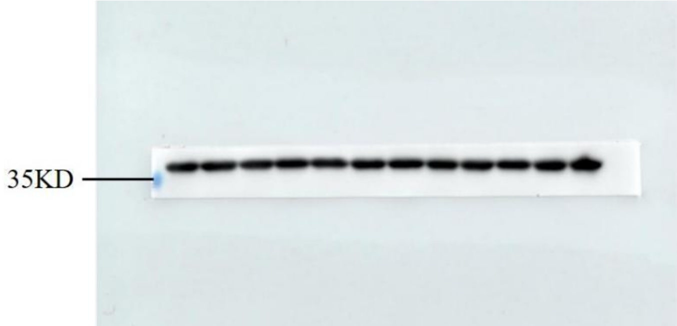

Slice 4, probed with antibodies to **FtH** (not provided in manuscript)

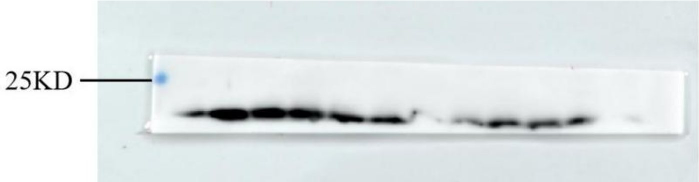

Supplement: Supplementary file 7 — Supplementary materials of all full scan the western blots [file 41419_2023_5688_MOESM7_ESM.pdf]
